# Tailoring Phosphonium Ionic Liquids for a Liquid–Liquid Phase Transition

**DOI:** 10.1021/acs.jpclett.3c00099

**Published:** 2023-03-20

**Authors:** Beibei Yao, Marian Paluch, Mateusz Dulski, Courtney Quinn, Shannon McLaughlin, Anne McGrogan, Malgorzata Swadzba-Kwasny, Zaneta Wojnarowska

**Affiliations:** † Faculty of Science and Technology, Institute of Physics, University of Silesia in Katowice, 75 Pułku Piechoty 1A, 41−500 Chorzów, Poland; ‡ Faculty of Science and Technology, Institute of Materials Science, the University of Silesia in Katowice, 75 Pułku Piechoty 1A, 41−500 Chorzów, Poland; § The QUILL Research Centre, School of Chemistry and Chemical Engineering, The Queen’s University of Belfast, David Keir Building, Stranmillis Road, BT9 5AG Belfast, Northern Ireland, U.K.

## Abstract

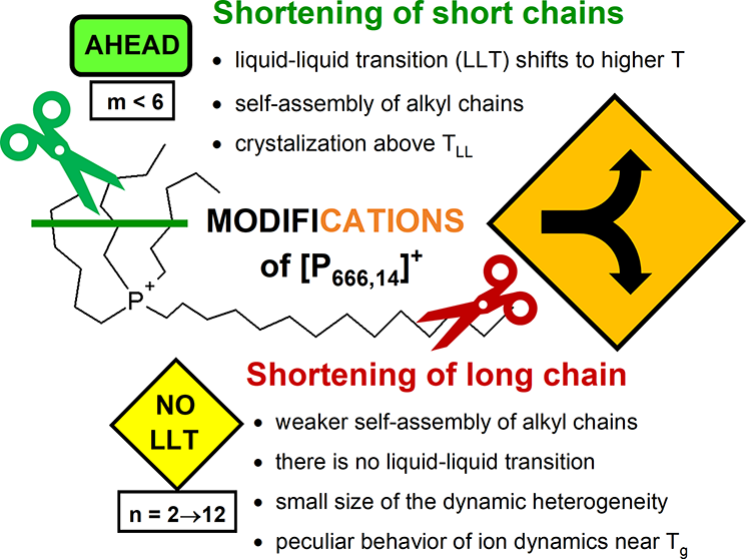

The existence of more than one liquid state in a single-component system remains the most intriguing physical phenomenon. Herein, we explore the effect of cation self-assembly on ion dynamics in the vicinity of liquid–liquid and liquid-glass transition of tetraalkyl phosphonium ([P_*mmm*,*n*_]^+^, *m* = 4, 6; *n* = 2–14) ionic liquids. We found that nonpolar local domains formed by 14-carbon alkyl chains are crucial in obtaining two supercooled states of different dynamics within a single ionic liquid. Although the nano-ordering, confirmed by Raman spectroscopy, still occurs for shorter alkyl chains (*m* = 6, *n* < 14), it does not bring calorimetric evidence of LLT. Instead, it results in peculiar behavior of ion dynamics near the liquid-glass transition and 20-times smaller size of the dynamic heterogeneity compared to imidazolium ionic liquids. These results represent a crucial step toward understanding the nature of the LLT phenomenon and offer insight into the design of efficient electrolytes based on ionic liquids revealing self-assembly behavior.

Within 100 years since the discovery of ethylammonium nitrate by Paul Walden,^1^ ionic liquids (ILs) have emerged as an exceptional class of molten salts with unique physical, chemical, and biological properties.^2,3^ The numerous possibilities for mixing, matching and incorporating different atoms or functional groups into ILs provide a unique opportunity to fine-tune their physicochemical properties for many industrial applications. Among others, ILs meet the needs of electrochemistry, energy storage, catalysis, engineering, and pharmacy.^4−6^

A key advantage of ILs over molecular fluids is their excellent ability to exhibit vitrification. Under cooling, low-viscosity liquid becomes a supercooled equilibrium fluid that transforms to a nonequilibrium amorphous solid at the glass transition temperature *T*_*g*_.^7^ Among the factors controlling the glass-forming ability of ILs, one can mention the symmetry of ionic species, charge delocalization, size of ions, and competing for interionic interactions (van der Waals vs Coulomb forces and H-bonding).^8,9^ For example, incorporating a small chloride anion into the IL structure increases *T*_*g*_.^10,11^ The same is achieved by introducing strong H-bonding interactions or elongating the alkyl chain in the cation.^12−14^ However, the latter can also lead to various short-range ordering structures, e.g., clusters or mesoscopic agglomerates,^15−17^ which might dramatically influence the properties of ILs.^18−20^ Therefore, understanding the molecular-level interactions within ILs is crucial for their industrial applications.

Recently, the self-organization of ions in quaternary phosphopnium-based ILs has been considered an origin of the first-order liquid–liquid phase transition (LLT).^21,22^ Specifically, on cooling, [P_666,14_][BH_4_] was transformed from one supercooled liquid state to another of different local structures, static dielectric permittivity, and thermodynamic properties. Compared to nonionic systems, including water,^23,24^ atomic elements (Si, Ge),^25,26^ triphenyl phosphite (TPP),^27,28^ water–trehalose mixtures,^29^ or metallic glasses,^30^ the LLT in [P_666,14_][BH_4_] can be classified as a genuine transition where both supercooled liquids can flow. It has also been shown that the exchange of borohydride anion to [TFSI]^−^, [TCM]^−^, [SCN]^−^, or taurine keeps both liquid phases and does not affect the temperature of LLT substantially. Furthermore, independently on anion, LLT was observed at a similar time scale of ionic motions (from 0.3 to 13 ms).^22^ All these facts suggest that the tendency of amphiphilic phosphonium cations to form local structures is responsible for LLT. In this context, the question naturally arises, *what is the critical length of alkyl chains to create nonpolar local domains and consequently to observe two different supercooled states within a single-component system?* In general, longer alkyl chains favor nano-organization. However, it has been reported that increasing the volume fraction of alkyl groups in the phosphonium ILs does not necessarily promote mesoscale aggregation.^31^ A detailed analysis of [P_222,12_][TFSI] and [P_444,12_][TFSI] has shown disruption of the mesoscale aggregates with increasing lengths of the shorter alkyl chains. At the same time, the LLT has not been observed in these materials. Therefore, a thorough understanding of the formation of heterogeneous microstructures by phosphonium ILs characterized by different combinations of alkyl chain lengths seems essential for the precise control of LLT.

To address this issue, we have synthesized two different sets of phosphonium ILs (see Supporting Information for details). The former contains ILs with trialkyltetradecyl phosphonium cation [P_*nnn*,14_]^+^, specifically [TFSI]^−^ and chloride salts of [P_444,14_] and [P_666,14_], while the ILs from the second group are characterized by trihexyl alkylphosphonium cations, [P_666,*n*_]^+^ (*n* = 2, 6, 8, 12), combined again with [Cl]^−^ and [TFSI]^−^ anions. The chemical structures of investigated ILs are shown in Figure 1, parts a and b. To verify whether the chosen systems undergo LLT, differential scanning calorimetry (DSC) is employed. Later on, the effect of cation self-assembly on the ion dynamics is examined by means of dielectric spectroscopy. Using the Raman measurements, we investigate a local organization of ILs over a broad temperature range.

**Figure 1:**
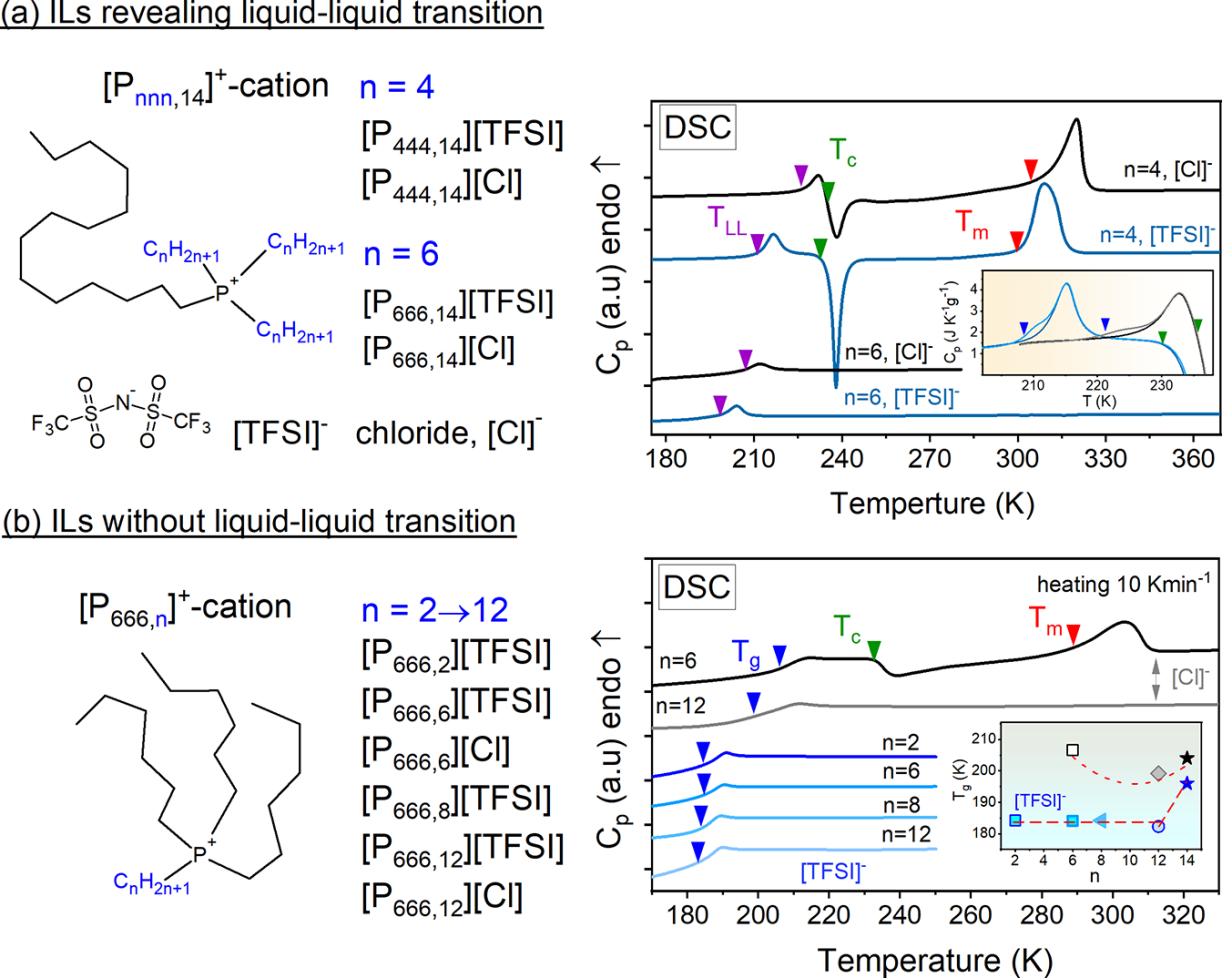
Chemical structures and DSC thermograms of ILs with (a) and without (b) LLT. The inset in DSC panel (a) is the comparison between DSC traces obtained during standard heating and after the aging process at 203.15 and 193.15 K for [P_444,14_][Cl] (gray) and [P_444,14_][TFSI] (light blue), respectively. The arrows indicate the characteristic temperatures of studied ILs: onset of LLT (violet arrows); onset of crystallization (green arrows); onset of melting (red arrows); glass transition temperature (blue arrows). The bottom inset presents the glass transition temperature as a function of alkyl chain length for examined [P_666,*n*_]^+^-based ILs.

The DSC technique has been used to provide thermal characteristics of [P_*nnn*,14_]^+^ and [P_666,*n*_]^+^-based ILs. The thermograms obtained on heating with the standard rate of 10 K/min are presented in Figures 1, parts a and b.

Calorimetric experiments show that all studied ILs could be vitrified on cooling; however, they reveal different behaviors during the heating process. In particular, tributyl- and trihexyl tetradecyl phosphonium ILs show well-resolved endothermic peaks identified with the liquid–liquid phase transition. At the same time, the DSC traces of studied [P_666,*n*_]^+^-based ILs (*n* = 2–12) exhibit a clear signature of liquid-glass transition and no signs of the LLT, even if *n* = 12. Hence, the 14-carbon chain seems necessary to induce a transition between two supercooled states. At the same time, modification of three other substituents while keeping the −[CH_2_]_13_–CH_3_ structure of the fourth one brings substantial differences in temperature and enthalpy of LLT, as well as affects the crystallization tendency of ILs. In particular, the shortening of three cation side chains from hexyl to butyl increases the enthalpy of LLT (especially for IL with [TFSI]^−^ anion *ΔH* increases twice) and shifts its onset to higher temperatures. *T*_*LL*_ rises by 11 K for ILs with [TFSI]^−^ anion and 20 K for chloride salts. The values of *T*_*LL*_ and *ΔH* are listed in Table S1. High enthalpy of LLT found for [P_444,14_]-ILs indicates that the alkyl chains self-assembly causing LLT is better constituted compared to [P_666,14_]-ILs, and therefore, they crystallize above *T*_*LL*_. This is visible as a sharp exotherm followed by an endotherm, revealing a subsequent melting process on DSC thermograms. Due to the sterical hindrance provided by the [TFSI]^−^ anion, liquid 1 of [P_444,14_][TFSI] shows a slightly weaker tendency to crystallize than it does for [P_444,14_][Cl].

As mentioned above, the shortening of a single alkyl chain in [P_666,14_]^+^ cation by the CH_2_–CH_2_– group, i.e., from 14 to 12 carbons, inhibits the ability of IL to undergo LLT and markedly decreases the temperature of the liquid-glass transition. Note that the *T*_*g*_ of ILs with LLT was identified on DSC scans by the time-dependent annealing (aging) experiments performed at *T* < *T*_*g*_. Then the liquid-glass transition becomes visible as a step-like change of heat capacity at *T* < *T*_*LL*_ (see the inset to Figure 1a and Table S1 for *T*_*g*_ values). Interestingly, a further shortening of alkyl chain length in [P_666,*n*_][TFSI] systems from *n* = 12 to *n* = 2 does not change the thermal properties. Namely, *T*_*g*_ remains constant, and none of them shows a crystallization tendency. That is at odds with the behavior of imidazolium-based ILs, where *T*_*g*_ was found to decrease for material with a shorter alkyl substituent.^12,13^ At the same time, in the case of [P_666,*n*_][Cl], *T*_*g*_ plotted as a function of alkyl chain length reveals a nonmonotonic behavior; i.e., it decreases with the elongation of the alkyl chain from 6 to 12 carbons and gets higher for C_14_. Furthermore, among all studied [P_666,*n*_] ^+^-systems, only [P_666,6_][Cl] reveals the crystallization tendency above *T*_*g*_.

A closer inspection of thermograms obtained for [P_666,*n*_]^+^-containing ILs (*n* = 2–12) reveals a substantial broadening of the glass transition step compared to classical ILs. Such a result is commonly identified with a broad distribution of structural relaxation times (larger dynamic heterogeneities) within the material and characterizes rather polymerized ionic liquids and multicomponent systems than simple low-molecular ILs.^32−34^ Therefore, one might expect the formation of some heterogeneous microstructures in [P_666,*n*_]^+^-based IL, although there is no evidence of LLT. To explore this issue thoroughly, the Donth^35,36^ approach defining the number of dynamically correlated particles, *N*_*α*_^*D*^, in the *T*_*g*_ region has been employed for [P_666,*n*_]^+^-ILs, (see Supporting Information for details). Interestingly, the obtained values of *N*_*α*_^*D*^(*T*_*g*_) are exceptionally low (less than ten) for all examined here [P_666,*n*_]^+^-ILs (see Table S1). It means that only several particles move cooperatively close to the glassy state. This indicates the existence of some aggregates with strong van der Waals interactions and short intermolecular distances between the alkyl chains of [P_666,*n*_]^+^-ILs. Such a conclusion is supported by recent reports where *N*_*α*_^*D*^(*T*_*g*_) was strongly correlated with the length of alkyl chain attached to the cation (more −CH_2_– groups, lower *N*_*α*_^*D*^).^11^ However, so far, the lowest reported value of *N*_*α*_^*D*^(*T*_*g*_) was equal to 20, and interestingly, it was found for IL with the phosphate anion, i.e., [C_1_C_2_Im][DBP]. Further dielectric studies were performed to verify whether such exceptionally small regions of dynamic heterogeneity affect the ion dynamics in [P_666,*n*_]^+^-ILs and how much their relaxation behavior is different from [P_*nnn*,14_]^+^-ILs that reveal a clear LLT.

To avoid cold crystallization and maintain the same thermal history, all examined herein ILs were initially quenched to the glass state, and the dielectric data were recorded during the heating scan. Parts a and b of Figure 2 show the dielectric modulus spectra *M″*(*f*) of representative ILs with and without LLT on DSC thermograms, that is, for [P_444,14_][TFSI] and [P_666,6_][TFSI].

**Figure 2:**
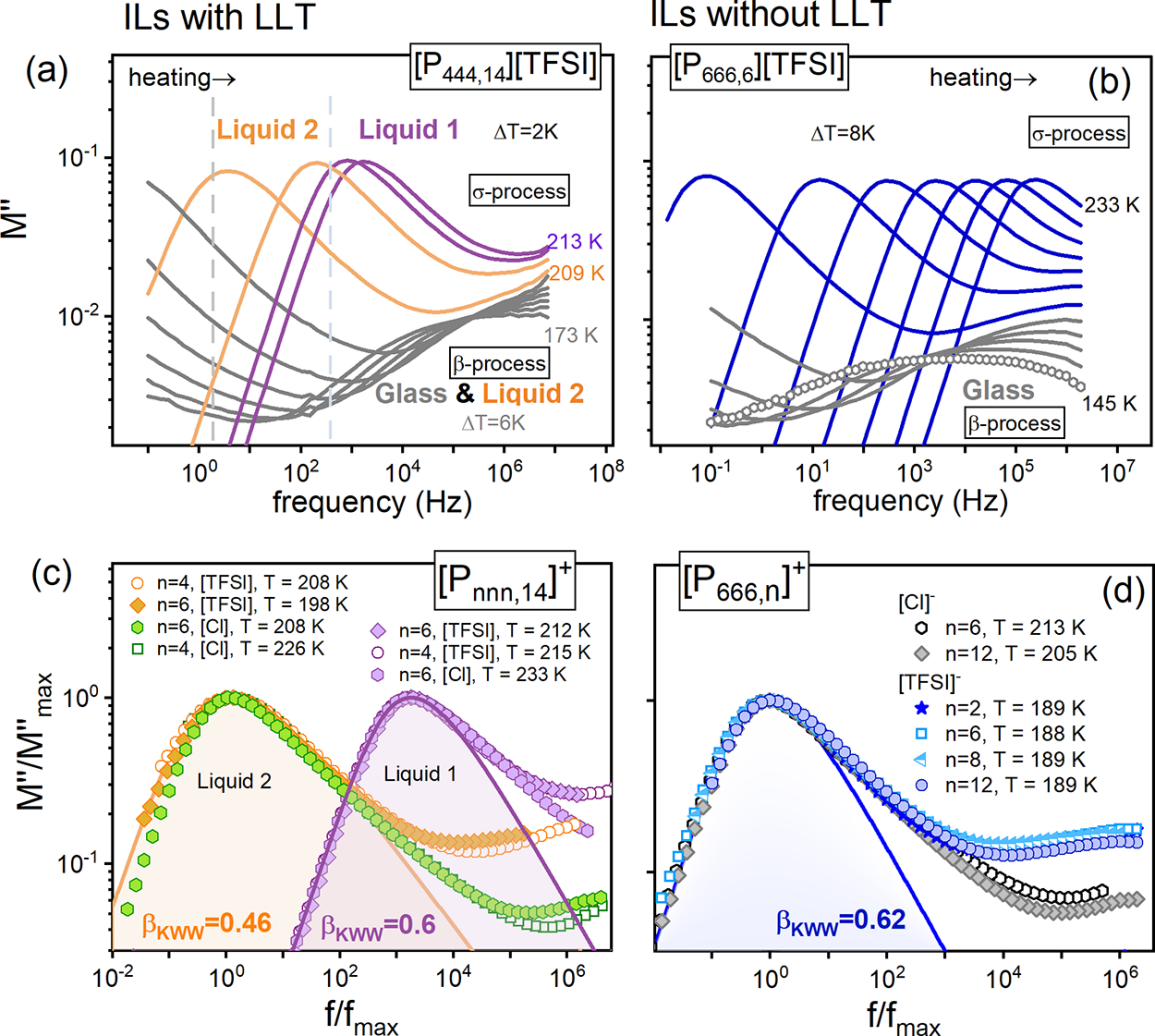
Representative electric loss modulus spectra of ILs with and without LLT, i.e., [P_444,14_][TFSI] (a) and [P_666,6_][TFSI] (b), recorded at various temperatures in the liquid and glassy states. The dashed lines in panel (a) separate the glass from liquid 2, and liquid 1. Panels c and d show the normalized *M″* spectra recorded for various ILs and various temperatures, however, with a similar frequency of the modulus peak maximum. The solid lines denote the fits of the Kohlrausch function, ϕ(*t*) = exp [−(*t*/*τ_σ_*)^*β*_*KWW*_^].

A single secondary relaxation (labeled as β-process) is visible in the glassy state of all studied ILs. The only difference is in the secondary process’s amplitude, which is more pronounced in ILs with [TFSI]^−^ anion. As the temperature increases, another relaxation mode, the so-called conductivity relaxation process (σ-process), related to the translational mobility of ions, becomes the main feature in the dielectric spectra. Similarly to other ionic glass formers, the *M″*-peak of each [P_666,*n*_]^+^ and [P_*nnn*,14_]^+^-based ILs shifts toward higher frequencies on heating. However, in the latter cases, the temperature sensitivity of σ-mode changes substantially from liquid 2 to liquid 1. Furthermore, from the data normalization presented in panels c and d of Figure 2, it is evident that the *M″*(*f*) spectra are getting narrower when the temperature increases above *T*_*LL*_^DSC^. Meanwhile, the *M″*(*f*) peak of [P_666,*n*_]^+^-ILs keeps the same shape at various *T* values over the supercooled state and for different lengths of fourth alkyl chain (*n* = 2–12) (see Figure 2, parts c and d, and Table S1).

In the next step, we have constructed the relaxation maps to highlight the effect of IL morphology on ion dynamics above and below *T*_*g*_. The characteristic of local dynamics (β-processes) is presented in Figure S2 in the Supporting Information. One can note that the glassy dynamics of [P_666,*n*_]^+^-ILs and [P_*nnn*,14_]^+^-ILs are very similar. Namely, there is a single β-process with the activation energy oscillating around 35 ± 2 kJ/mol for ILs with [TFSI]^−^ anion and *E*_*a*_ = 28 ± 3 kJ/mol for chloride salts of tetraalkyl phosphonium liquids. However, when we consider a given *T* < *T*_*g*_, the dependence between the alkyl chain length and the time scale of β-relaxation occurs. In particular, the local dynamics slows down with the elongation of alkyl substituent from 2 to 14 carbons; however, at the same time, it is insensitive to structural changes arising from LLT. Considering the relatively low energy consumption of β-relaxation, some intramolecular motions within phosphate cation are expected for the occurrence of this mode in the *M*″(*f*) spectra.

Figure 3a shows the temperature evolution of conductivity relaxation times (*τ*_*σ*_ = 1/2π*f*_max_) determined for ILs containing [P_444,14_]^+^ and [P_666,14_]^+^ cations. As can be seen, the dynamics of both examined [P_666,14_]^+^-systems follows the Vogel–Fulcher–Tammann (VFT) in liquid 1 state and markedly changes the behavior at *T*_*LL*_^DSC^. At the same time, the *τ*_*σ*_(*T*) data of [P_444,14_]^+^-ILs are unavailable above their *T*_*LL*_ due to the strong crystallization tendency. Therefore, the temperature dependence of dc-conductivity log *σ*_*dc*_ (*T*^–1^) determined in the vicinity of *T*_*m*_ has been employed to characterize their ion dynamics in the liquid 1 state. As can be seen, around calorimetric LLT, the VFT fit, representing the behavior of *τ*_*σ*_ in liquid 1, meets the experimentally determined *τ*_*σ*_ (*T*^–1^) in liquid 2 state. This procedure enables us to estimate the time scale of conductivity relaxation at *T*_*LL*_ for [P_444,14_][TFSI] and [P_444,14_][Cl]. From a direct comparison of *τ*_*σ*_ (*T*^–1^) dependences for both examined [Cl]^−^ salts, one can note that *τ*_*σ*_ (*T*_*LL*_) is almost the same (∼0.1 s). However, an exchange of [Cl]^−^ anion to [TFSI]^−^ shifts *τ*_*σ*_(LLT) almost three decades toward shorter relaxation times ([P_444,14_][TFSI], 0.8 ms; [P_666,14_][TFSI], 5 ms). Interestingly, the time scale of charge transport at *T*_*g*_ also differs for [P_*nnn*,14_]^+^-based ILs, and it is much shorter than 100 s (log *τ*_*σ*_(*T*_*g*_) = 2) commonly identified with the freezing of ions mobility at *T*_*g*_. Specifically, log *τ*_*σ*_(*T*_*g*_) = 0.5 and −1.5 for [P_444,14_][Cl] and [P_444,14_][TFSI], respectively. It means that the charge transport still occurs when the structural dynamics become arrested. Such a decoupling between charge transport and structural relaxation in aprotic ionic liquids needs further examination. An exchange to [P_666,14_] makes the ILs more coupled (log *τ*_*σ*_(*T*_*g*_) = 1.5 and 0 for [P_666,14_][Cl] and [P_666,14_][TFSI]) compared to [P_444,14_]^+^-ILs. The liquid–liquid and liquid-glass transitions are also clearly detectable when the Stickel analysis (d[log_10_*τ*_*σ*_]/d[1000/*T*])^−0.5^ is performed. For conventional ILs with single VFT behavior, the Stickel operator transforms the VFT function into a linear dependence. On the other hand, when two VFT equations are required to parametrize the experimental data, two linear regions intersect at certain temperatures, usually called *T*_b_. In the latter case, the Stickel plot indicates a fragility change when passing the crossover temperature. As seen in the bottom panel of Figure 3a, the data deviate from the linear behavior around *T*_*LL*_ and reveal a minimum at *T*_*g*_. Note that at T_LL_, the slope of (d[log_10_*τ*_*σ*_-/d[1000/*T*])^−0.5^ dependence is getting larger, which is in contrast to the Stickel graph of any other glass-forming liquid,^37^ and therefore, it can be treated as a dynamic signature of LLT.

**Figure 3:**
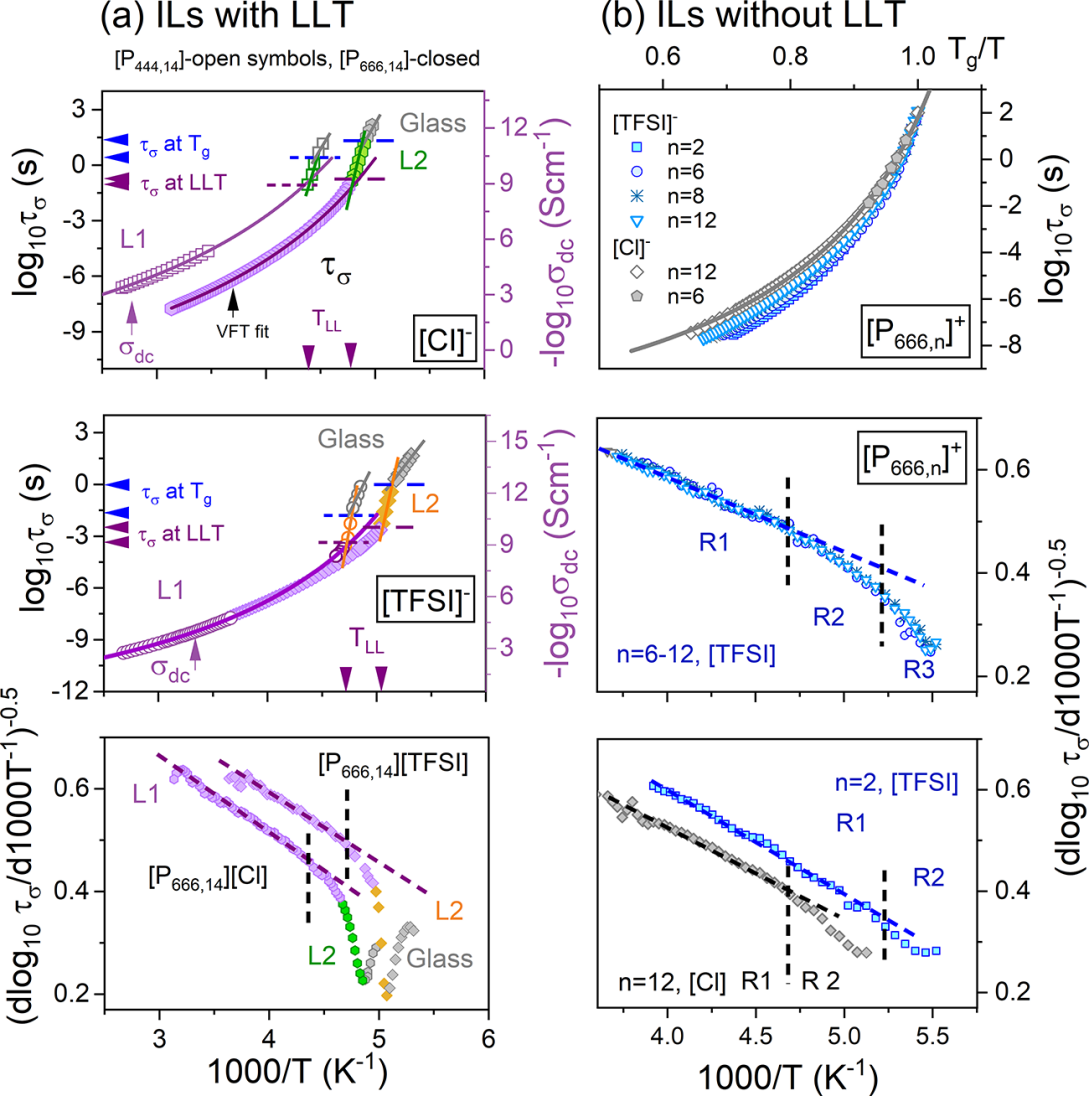
Temperature dependence of *τ*_*σ*_ and dc-conductivity *σ*_*dc*_ for chloride and TFSI salts of [P_444,14_]^+^ and [P_666,14_] ^+^ (a) and [P_666,*n*_]^+^-based ILs (b). Solid lines indicate the fits of the VFT function to dc-conductivity data recorded in liquid 1 state. Violet arrows denote the *τ*_*σ*_ and *T*_*LL*_ at LLT, while blue arrows denote the *τ*_*σ*_ at *T*_*g*_. Temperature evolution of *τ*_*σ*_ of [P_666,*n*_][TFSI] and [P_666,*n*_][Cl] is presented in the *T*_*g*_/*T* scale. The bottom panels present the Stickel plots. The dashed lines denote the intersection of distinct linear regions (R).

At first sight, the temperature dependence of conductivity relaxation times determined for [P_666,*n*=2–12_]^+^-ILs resembles classical ILs with the single VFT-type temperature dependence of *τ*_*σ*_ and *T*_*g*_ = *T* (*τ*_*σ*_ = 100 s). However, a derivative analysis reveals again a unique curvature of the Stickel plot, similar to that found for [P_666,14_]^+^-ILs undergoing LLT. Namely, at a specific temperature (d[log_10_*τ*_*σ*_]/d[1000/*T*])^−0.5^ data of [P_666,*n*_]^+^-ILs (*n* = 2–12) depart from the high-temperature linear regime (R1) with a deviation degree being to some extent dependent on anion and alkyl-chain length. Precisely, the slope change of Stickel plot is almost negligible for [P_666,2_][TFSI] and [P_666,12_][Cl], while three regions of different grades can be detected for [P_666,*n*_][TFSI], *n* = 6–12 (see Figure 3b). Raman spectroscopy has been employed to reveal the molecular origin behind this observation.

As a structurally sensitive technique, Raman measurements have been performed for [P_666,12_][TFSI], [P_666,2_][TFSI], [P_666,14_][TFSI], and [P_444,14_][TFSI], the latter two as representative ILs with LLT. The obtained results are depicted in Figure 4. The attention has been focused on the analysis of vibrational modes of the alkyl chains, including (i) stretching mode within the chain (1025–1125 cm^–1^) and (ii) deformational modes of CH_*x*_ (*x* = 2,3) (1275–1525 cm^–1^) as well as (iii) the stretching vibration of CH_*x*_ (2800–3050 cm^–1^).^38^ The first region is sensitive to the conformational order, the second corresponds to the degree of coupling, and the latter provides information about the ordering of alkyl chains. As can be seen in Figure 4d, cooling of [P_444,14_][TFSI] from liquid 1 to liquid 2 brings a slight upshift (∼3 cm^–1^) of ν(C–C)-related bands (at 1053 and 1098 cm^–1^, and τ(CH_2_)-modes at 1306 cm^–1^) and downshifting of CH_*x*_-bands (∼3 cm^–1^) within the 2800–3050 cm^–1^ range. All these spectral changes point to a molecular reorganization, which in analogy to data described recently for [P_666.14_][BH_4_], confirmed chain coupling and a more ordered arrangement of alkyl tails in liquid 2 of [P_444,14_][TFSI]. Note that the Raman spectra recorded for supercooled 1 and 2 states markedly differ from that of crystalline [P_444.14_][TFSI] (see a gray line in Figure 4d). Specifically, crystallization was observed as increased intensity and narrowing of bands corresponding to SO_2_ vibrations in [TFSI]^−^ anion and ν(C–C), τ(CH_2_), and δ(CH_3_) in the [P_444,14_]^+^ cation. Furthermore, the stretching-related modes, including *v*_s_(CH_2_) and ν_as_(CH_2_) are shifted to 2853 and 2883 cm^–1^, respectively. Such changes result from an increase in molecular packing related to crystal-like ordering.^39,40^

**Figure 4:**
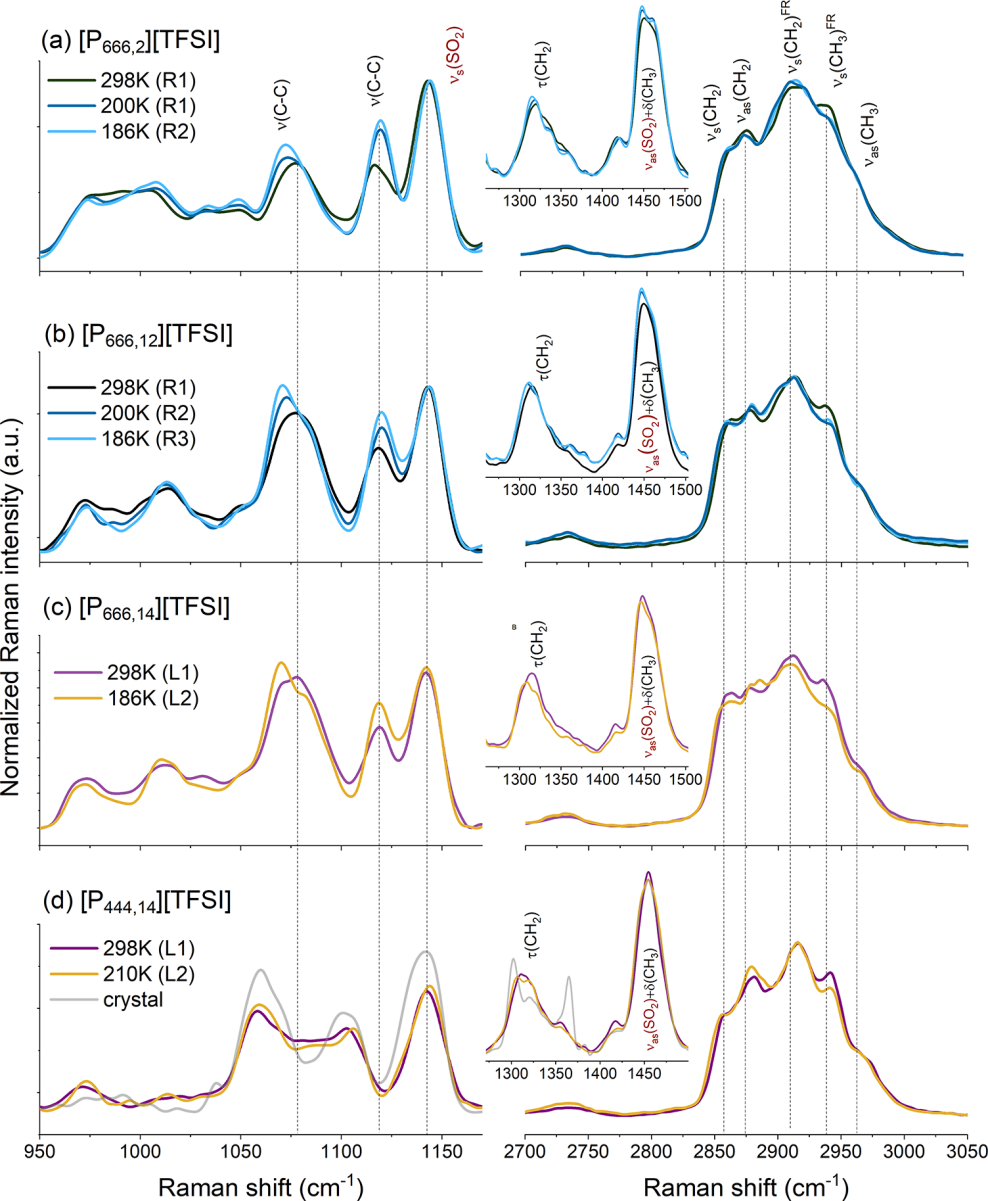
Raman measurements of selected ILs recorded at three different temperatures corresponding to liquid 1 (L1) and liquid 2 (L2) states of [P_444,14_][TFSI] and [P_666,14_][TFSI] as well as different regions (R1, R2, R3) marked on the Stickel plot for [P_666,12_][TFSI] and [P_666,2_][TFSI]. The comparison between the spectral lines recorded at room temperature of [P_444,14_][TFSI], [P_666,12_][TFSI], and [P_666,2_][TFSI] is presented in the Supporting Information.

Parts a and b of Figure 4 show the Raman data recorded for [P_666,12_][TFSI] and [P_666,2_][TFSI]. In analogy to [P_666__,14_][TFSI], cooling brings higher intensity and downshift (∼2–3 cm^–1^) of ν(C–C) (from 1071 cm^–1^) and τ(C–C) (at 1310 cm^–1^) bands. At the same time, these related to the stretching of ν(CH_2_) and ν(CH_3_) are oppositely shifted (to 2858 and 2941 cm^–1^, respectively). Additionally, a slight shift of the δ(CH_2_) up to 1447 cm^–1^ suggests a slowing down alkyl chains’ local motions. All these facts are clear evidence of aliphatic chain coupling due to their mutual ordering, which is similar to that accompanying LLT (see Figure 4c for comparison). Interestingly, decreasing temperature from 200 to 186 K brings only an intensity increase of the LLT-sensitive bands without their shift, evidencing further molecular nanosegregation of [P_666,12_][TFSI] and [P_666,2_][TFSI]. These changes are more pronounced for IL with a 12-carbon long aliphatic chain than for [P_666,2_]^+^, suggesting weaker nano-ordering of the latter.

In summary, our experimental Raman studies revealed the local arrangements of alkyl chains in all examined herein tetraalkyl phosphonium ILs; however, only [P_444,14_]^+^- and [P_666,14_]^+^-based systems undergo a liquid–liquid phase transition. The LLT has been disclosed by an endotherm peak on the DSC heating scan and the substantial departure of ion dynamics (*τ*_*σ*_, *σ*_*dc*_) from VFT behavior. High enthalpy of LLT found for [P_444,14_]^+^-ILs compared to [P_666,14_]^+^-ILs indicates that the alkyl chains self-assembly is better constituted in the former case, and therefore brings spontaneous crystallization just above *T*_*LL*_. On the other hand, for [P_666,*n*_]^+^-based ILs, there is no sign of LLT shown in thermodynamic and dynamic properties. However, an analysis of dielectric data in terms of the Stickel operator gave the results to some extent similar to that observed for ILs with LLT; that is, negative deviation from the high-temperature linear regime. Thus, shortening the aliphatic chain reduces the possibility of LLT but keeps the potential of ILs for partial nanorganization. In turn, the latter brings peculiar ion dynamics behavior near *T*_*g*_ and exceptionally small regions of dynamic heterogeneity *N*_*α*_(*T*_*g*_) (below 10) determined from Donth analysis of calorimetric measurements.

## Supporting Information Available

Experimental protocols, the calculation of dynamically correlated particles, and collected tables for thermodynamic and dynamic properties of studied ILs (PDF)
