# Supplementary material for: Tailoring Phosphonium Ionic Liquids for a Liquid–Liquid Phase Transition
Source: J Phys Chem Lett. 2023 Mar 20;14(12):2958–64. doi: 10.1021/acs.jpclett.3c00099 (PMC10068824; doi:10.1021/acs.jpclett.3c00099)
Supplement: Supplementary file 1 — jz3c00099_si_001.pdf [file jz3c00099_si_001.pdf]

Supplementary information

Tailoring Phosphonium Ionic Liquids for Liquid-Liquid Phase Transition

B. Yao[1], M. Paluch[1], M. Dulski[2], C. Quinn[3], S. McLaughlin[3], A. McGrogan,[3] M. Swadzba-Kwasny[3], Z. Wojnarowska[1*]

[1]*Faculty of Science and Technology, Institute of Physics, University of Silesia in Katowice, 75 Pułku Piechoty 1A, 41–500 Chorzów, Poland*

[2]*Faculty of Science and Technology, Institute of Materials Science, the University of Silesia in Katowice, 75 Pułku Piechoty 1A, 41–500 Chorzów, Poland*

[3]*The QUILL Research Centre, School of Chemistry and Chemical Engineering, The Queen's University of Belfast, David Keir Building, Stranmillis Rd, BT9 5AG Belfast, NI, UK.*

**corresponding author* zaneta.wojnarowska@smcebi.edu.pl

Experimental Methods

Differential Scanning Calorimetry (DSC)

Calorimetric experiments of studied ILs were carried out by a Mettler Toledo DSC1STAR System equipped with a liquid nitrogen cooling accessory and an HSS8 ceramic sensor (a heat flux sensor with 120 thermocouples). The gas nitrogen keeps flowing at 60 mL min^{-1} during the whole experiment. The DSC device was calibrated for enthalpy and temperature using indium and zinc standards, as well as *n*-heptane (182.15 K, 140.5 Jg^{-1}) at different scanning rates (0.7, 1, 5, and 10 Kmin^{-1}) for low-temperature verification. The DSC curves were exported by a dedicated software Mettler Toledo DSC1STAR, allowing various calculations (heat capacity, onset, normalized enthalpy, etc.) for the original heat flow curves. The baseline was constructed as a straight line from the onset to the endpoint. All DSC measurements were performed from 373 K cooling to 123 K and then heating to 373 K with a rate of 10 K min^{-1}. The 6-hour aging experiments were performed at 203.15 K and 193.15 K for [$P_{444,14}$][Cl] and [$P_{444,14}$][TFSI], respectively. The liquid-liquid transition temperature and melting point were obtained at the onset of the peak, while the glass transition temperature was determined as the midpoint of the heat capacity increment.

Based on the fluctuation-dissipation theorem, Donth proposed that the volume of one average cooperatively rearranging region (CRR) at T_g can be calculated by the following equation:

$$V_\alpha(T_g) = \frac{k_B T_g{}^2\left(\frac{1}{C_p{}^{glass}} - \frac{1}{C_p{}^{liquid}}\right)}{\rho \delta T^2}\Bigg|_{T_g} \quad (1)$$

where $k_B = 1.38\times10^{-23}$ J K^{-1} is the Boltzmann constant, ρ is the density of the bulk material. $c_p{}^{liquid}$ and $c_p{}^{glass}$ represent the isobaric heat capacities of liquid and glass at T_g. δT denotes the average temperature fluctuation of a CRR related to the dynamic glass transition. $\delta T = \Delta T/2.5$ is the average temperature fluctuation calculated as the temperature interval ΔT where the heat capacity changes from 16 to 84% of the total heat capacity step ΔC_p at T_g (see Figure S1). N_α is the number of particles in one average CRR with volume V_α. Thus N_α can be calculated by:

$$N_\alpha(T_g) = \frac{V_\alpha(T_g)\rho N_A}{M} = \frac{k_B T_g^2}{M(\delta T)^2} N_A\left(\frac{1}{c_p^{glass}} - \frac{1}{c_p^{liquid}}\right) \quad (2)$$

where M is the molar mass, $N_A = 6.02\times10^{23}$ mol^{-1} is the Avogadro constant.

The scheme for the determination of the $N_\alpha{}^D(T_g)$ is presented in Figure S1, taking [$P_{666,8}$][TFSI] as the reference. Other quantities are collected in Table S1.

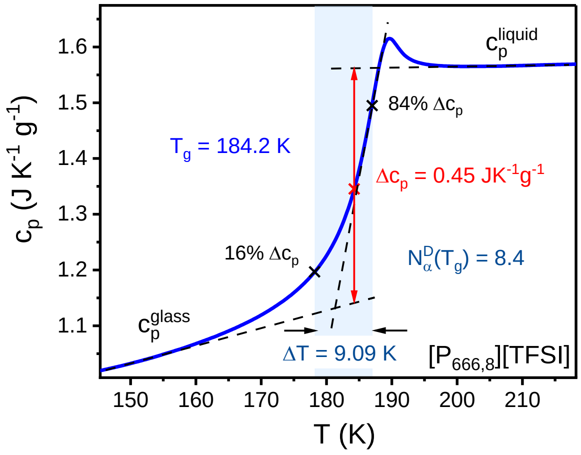

Figure S1 The scheme for the determination of the $N_\alpha{}^D(T_g)$ using Donth method (eq 2) for [$P_{666,8}$][TFSI].

Broadband Dielectric Spectroscopy (BDS).

Dielectric measurements were performed by means of a Novo-Control GMBH Alpha impedance analyzer over a frequency range from 10^{-2} Hz to 10^{7} Hz at different temperatures corresponding to the supercooled liquid state of studied ILs. During the measurements, the sample was placed between two stainless steel electrodes (diameter = 15 mm) with a fixed distance (0.1 mm) provided

by the quartz ring, and the temperature was controlled by a Quatro system using a nitrogen gas cryostat with an accuracy of 0.1 K. Additionally, $[P_{444,14}][Cl]$ and $[P_{666,6}][Cl]$ were heated up above melting temperature with the capacitor plate before measurements.

When the studied ILs supercooled from liquid to glassy state, the localized motions have been found in modulus spectra. These local motions are known as secondary (β-) relaxation processes. In order to analyze and interpret the thermal behavior of β-relaxation, we performed a numerical fitting analysis of *M''(f)* data below T_g, in terms of the Cole-Cole function,

$$M^*(\omega) = 1/\varepsilon^*(\omega) = \left(\varepsilon_\infty + \frac{\Delta\varepsilon}{[1+(i\omega\tau_{CC})^{\alpha_{CC}}]^1}\right)^{-1} \quad (3)$$

where $\Delta\varepsilon$ is the dielectric strength, ε_∞ is the high-frequency limit permittivity, τ_{CC} defines the characteristic relaxation time and α_{CC} is shape parameter. The characteristic relaxation times of β-process can be calculated according to the fitting parameters:

$$\tau_\beta = \tau_{CC}\left[sin\left(\frac{\alpha_{CC}\cdot\pi}{2+2}\right)\right]^{-1/\alpha_{CC}}\left[sin\left(\frac{\alpha_{CC}\cdot 1\cdot\pi}{2+2}\right)\right]^{1/\alpha_{CC}} \quad (4)$$

The obtained $\log_{10}\tau_\beta$ as function of 1000/*T* have been plotted in Figure S2. As can be seen, all the secondary processes obey the Arrhenius law:

$$\tau_\beta = \tau_\infty exp(\frac{E_a}{RT}) \quad (5)$$

where τ_∞ is the pre-exponential factor, *R* is the gas constant, and E_a denotes the energy barrier. The values of E_a are listed in Table S1 for all studied ILs.

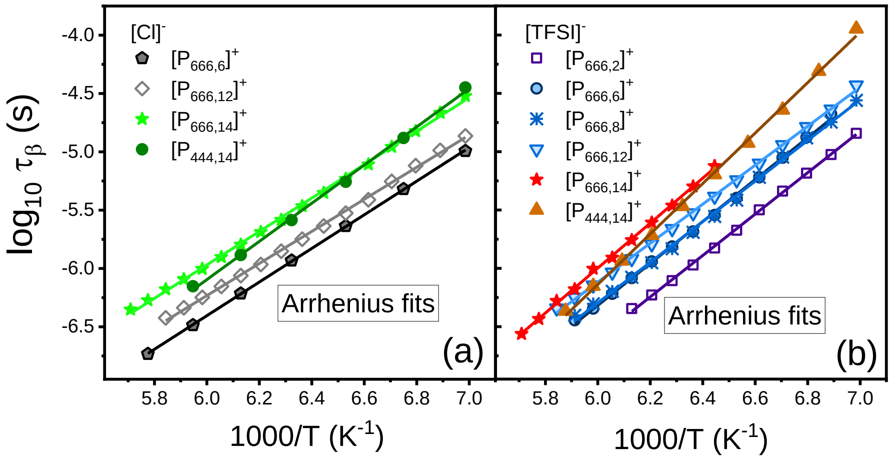

Figure S2 Relaxation map of secondary relaxations of all studied ILs with anion $[Cl]^-$ (a), and $[TFSI]^-$ (b). Solid lines are fits of the Arrhenius law to experimental data.

Raman measurements

Raman measurements were performed using a WITec confocal Raman microscope (CRM) alpha 300R with a solid-state laser (λ = 532 nm) coupled into the microscope through a polarization-maintaining single-mode optical fiber with a 50 μm diameter. The laser radiation was focused onto the sample via a long-distance Olympus MPLAN (50x/0.76NA) objective, while then the scattered light passed through a multi-mode fiber (50 μm diameter). The spectrometer monochromator working with a 600 line/mm grating has been checked before the measurements using a silicon plate (520.7 cm^{-1}). The sample, put on the cover glass as a tiny droplet, was next mounted on the THMS600 Linkam stage. Raman spectra of TFSI salts of [$P_{666,12}$], [$P_{666,2}$] and [$P_{444,14}$] at room temperature were gathered at 20 mW on the sample using ten scans, an integration time of 20 s, and a resolution of 3 cm^{-1}. Then, individual samples were cooled down to 200 K, 186 K ($P_{666,12}$, $P_{666,2}$), and 210 K ($P_{444,14}$) at 100 K/min colling rate with 0.1 K stabilization temperature. At each interested temperature, Raman spectra were accumulated using ten scans, an integration time of 20 s, and a resolution of 3 cm^{-1}. All collected spectra were subjected to a post-processing analysis, including cosmic ray removal and baseline correction, using WITec Project Five Plus software. Finally, Raman spectra were normalized to the most intense band (e.g., 745 cm^{-1}), which high-intensity results from a large polarizability change due to the expansion and contraction of the whole $TFSI^-$ anion [J. Raman Spectrosc. 2005; 36: 762–770]. A proposed normalization stems from the comparable amount of the $TFSI^-$ anion in all samples and their insensitivity to conformational changes due to temperature [Herstedt M, Smirnov M, Johansson P, Chami M, Grondin J, Servant L, Lassegues JC. J. Raman Spectrosc. 2005; 36: 762; 10.1021/acsami.2c14057].

As the first, spectra of [$P_{666,12}$], [$P_{666,2}$], [$P_{666,14}$] and [$P_{444,14}$] obtained at room temperature showed a similar number of the bands insensitively to the changeable CH_2 content but with lower intensity depending on the aliphatic length. Differences in the bands' intensity depending on the ILs are observed by comparing individual spectra in the CH_x-related ranges (Fig. 3S panel a). In turn, the varied proportion between short and long chains correlates only with the shift of aliphatic-related bands without altering the position of the CH_x-related bands (Fig. S3 panel a). Thus, the most crucial, from the analytical point of view, aliphatic ν(C-C)-related bands are around 1056/1076 cm^{-1} and 1101/1117 cm^{-1} ([$P_{444,14}$]/[$P_{666,12}$], [$P_{666,2}$]), twisting τ(CH_2)-modes appear at 1310/1315/1318 cm^{-1} ([$P_{444,14}$]/[$P_{666,12}$]/[$P_{666,2}$]) and stretching ν(CH_x, x=2,3)-related bands occur at 2856/2880/2941 cm^{-1} ($\nu_s CH_2/\nu_{as} CH_2/\nu_s CH_3$ for [$P_{444,14}$]), 2860/2877/2938 cm^{-1} ($\nu_s CH_2/\nu_{as} CH_2/\nu_s CH_3$ for [$P_{666,12}$]), 2865/2878/2943 cm^{-1} ($\nu_s CH_2/\nu_{as} CH_2/\nu_s CH_3$ for [$P_{666,2}$]). Importantly, their position turned out to be slightly shifted concerning other literature-available data (Fig. 3S panel b) due to the type of chains and the mutual interaction between cation and $TFSI^-$ anion. Other δ(CH_x, x=2,3)-related bands with maxima around 1455/1447cm^{-1} ([$P_{444,14}$]/[$P_{666,12}$], [$P_{666,2}$]) turned out less sensitive to the structural reorganization associated with the LLT.

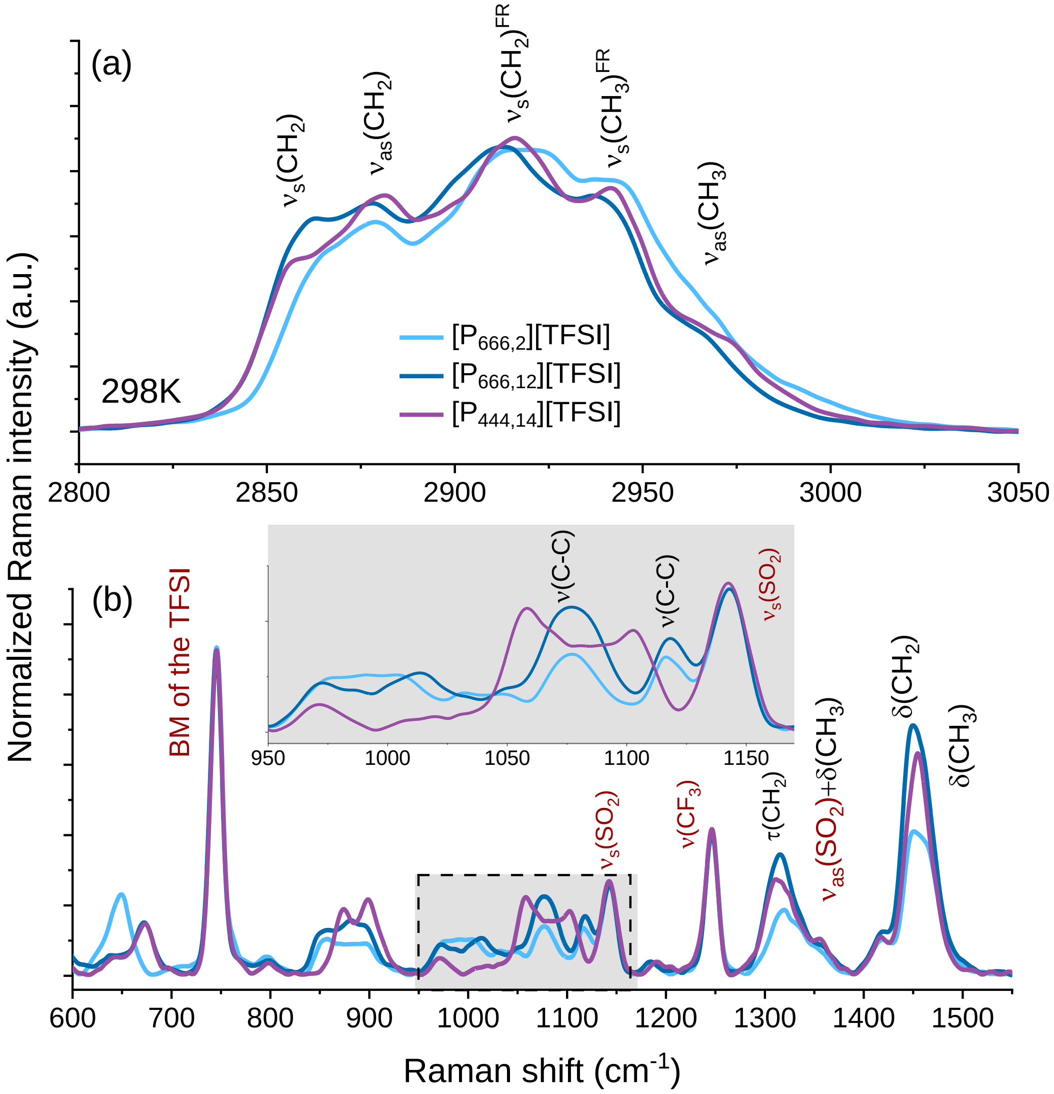

Figure S3 Raman measurements of examined ILs at RT.

Table S1 Thermodynamic and dynamic properties of studied ILs. Molar masses, M; glass transition temperature, T_g; liquid-liquid transition temperature, T_{LL}; crystallization temperature, T_c; and melting temperature, T_m. ΔH_{LL} denotes enthalpy of liquid-liquid transition; ΔH_c enthalpy of crystallization; and ΔH_m enthalpy of melting, all determined during the heating scan. The isobaric heat capacities at T_g for liquid, c_p^{liquid}, and glass, c_p^{glass}. Temperature interval ΔT determined from the $c_p(T)$ curve between 16% and 84% of the Δc_p step. Number of dynamically correlated molecules, $N_\alpha^D(T_g)$. *wt* water content. β_{KWW} stretching parameter. m_P dynamic fragility. E_a activation energy of secondary dynamics.

ILs	$[P_{666,14}]$ [Cl]	$[P_{666,14}]$ [TFSI]	$[P_{444,14}]$ [Cl]	$[P_{444,14}]$ [TFSI]	$[P_{666,6}]$ [Cl]	$[P_{666,12}]$ [Cl]	$[P_{666,2}]$ [TFSI]	$[P_{666,6}]$ [TFSI]	$[P_{666,8}]$ [TFSI]	$[P_{666,12}]$ [TFSI]
M (g/mol)	519.31	763.24	404.17	648.87	376.12	460.28	564.71	620.81	648.87	704.97
T_g (K)	197.9* 201**	195.9* 194.2**	221.2* 223**	208.5* 209**	206.6* 201.3**	199.1* 195.2**	184.2* 181.3**	184.1* 181.5**	184.2* 180.6**	182.3* 181.2**
T_{LL} (K)	205.9* 210**	200.1* 201.2**	227.1* 228**	211.5* 212**	-	-	-	-	-	-
ΔH_{LL} (J/g)	5.23	6.6	5.73	11.14	-	-	-	-	-	-
T_c (K)	-	-	233.8	236.1	232.6	-	-	-	-	-
$-\Delta H_c$ (J/g)	-	-	63.52	66.97	8.45	-	-	-	-	-
T_m (K)	-	-	309.8	301.7	288.9	-	-	-	-	-
ΔH_m (J/g)	-	-	62.78	65.26	8.65	-	-	-	-	-
c_p^{liquid} (J/K g)	-	-	-	-	1.95	1.31	1.42	1.35	1.57	1.54
c_p^{glass} (J/K g)	-	-	-	-	1.55	0.81	0.98	0.94	1.12	0.87
ΔT (K)	-	-	-	-	9.8	17.2	11.1	10.8	9.1	14
N_α^D (T_g)	-	-	-	-	8.2	7.1	8.1	7.9	8.4	6.5
wt (ppm)	899	683	1238	80	1553	2096	200	600	120	680
β_{KWW}	0.6 L1 0.58 L2	0.6 L1 0.52 L2	0.6 L1 0.48 L2	0.6 L1 0.46 L2	0.62	0.62	0.62	0.62	0.62	0.62
m_p (T_g)	-	-	-	-	68	73	80	101	90	89
E_a^β (kJ/mol)	27.4	36.9	31.3	41.4	27.6	26.4	34	34.4	33.4	32

*The glass transition temperature and LLT temperature were determined from DSC measurements.
**The glass transition temperature and LLT were determined from BDS.

Samples synthesis and characterization

Lithium bis(trifluoromethanesulfonyl) imide was bought from 3M and used as received. Trihexylphosphine was kindly provided by Solvay (Cytec). All other chemicals were sourced from Sigma-Aldrich and used as received, unless otherwise stated.

XRF analysis was performed on a Rigaku NEX QC+ QuantEZ High-Resolution Energy Dispersive X-ray Fluorescence (EDXRF) Spectrometer. NMR spectra were recorded on a Bruker Avance III 400 MHz spectrometer in acetonitrile-d_3. CHNS analysis was conducted on a Perkin Elmer 2400 Series II in a quartz combustion tube using acetanilide and cysteine as standards. TOF Mass spectrometry was performed using a Waters Xevo G2-XS Q-Tof. All samples were dissolved in acetonitrile for CHNS and TOF-MS analysis.

[$P_{666,14}$]Cl. Trihexyl(tetradecyl)phosphonium chloride, was provided by Ionic Technologies and was used as received.

[$P_{666,14}$][TFSI]. Trihexyl(tetradecyl)phosphonium chloride, [$P_{666,14}$]Cl (0.010 mol eq.) and lithium bis(trifluoromethanesulfon)imide Li[TFSI] (0.013 mol eq.) were separately dissolved in 25 cm^3 deionised water (18.2 MΩ.cm) (total 50 cm^3) and then combined in a round-bottomed flask (250 cm^3), resulting in the formation of a biphasic liquid system; the mixture was left to react (1 h, room temperature, 600 rpm). The aqueous layer was separated, and the organic layer was collected and washed, firstly with deionized water (18.2 MΩ.cm) (10 cm^3) and then dichloromethane, DCM (10 cm^3). Subsequent washes were performed with solution of Li[TFSI] in deionised water (18.2 MΩ.cm). Final three washes were performed with deionised water (18.2 MΩ.cm) until no chloride could be detected with silver nitrate solution. Subsequently, DCM was removed *via* rotary evaporation (30 min, 308.15 K) and the ionic liquid was dried under high vacuum (12h, 343.15 K, 10^{-2} mbar). XRF analysis confirmed chloride content was below the detectable limit. ^{1}H, ^{13}C, ^{19}F and ^{31}P NMR spectra of the IL were recorded in d_6-DMSO.

^{1}H NMR (400.13 MHz, d_6 -DMSO) δ: 0.83-0.92 (m, 12H), 1.21-1.27 (m, 18H), 1.28-1.35 (m, 14H), 1.35-1.43 (m, 8H), 1.43-1.55 (m, 8H), 2.11-2.23 (m, 8H).

^{13}C{^{1}H} NMR (100.61 MHz, d_6 -DMSO) δ: 13.53 (s, P-$(CH_2)_5$-***CH_3***), 13.64 (s, P-$(CH_2)_{13}$-***CH_3***), 17.45 (d, $1J_{C/P}$ = 188 Hz, P-***CH_2***-$(CH_2)_4$-CH_3), 17.52 (d, $1J_{C/P}$ = 192 Hz, P-***CH_2***-$(CH_2)_{12}$-CH_3), 20.46 (s,), 20.50 (s,), 20.54 (s,), 21.75 (s,), 22.07 (s,), 28.81 (d, $2J_{C/P}$ = 116 Hz, P-CH_2-***CH_2***-$(CH_2)_3$-CH_3), 28.90 (d, $2J_{C/P}$ = 128 Hz, P-CH_2-***CH_2***-$(CH_2)_{11}$-CH_3), 29.04 (s, P-$(CH_2)_4$-***CH_2***-CH_3), 29.09 (s,), 29.70 (d, $3J_{C/P}$ = 60 Hz, P-$(CH_2)_2$-***CH_2***-$(CH_2)_{10}$-CH_3), 29.95 (d, $3J_{C/P}$ = 60 Hz, P-$(CH_2)_2$-***CH_2***-$(CH_2)_2$-CH_3), 30.34 (s, P-$(CH_2)_3$-***CH2***-$(CH_2)_9$-CH_3), 31.32 (s, P-$(CH_2)_3$-***CH_2***-CH_2-CH_3), 119.50 (q, $1J_{C/F}$ = 1280 Hz CF_3).

^{19}F NMR (376.50 MHz, d_6 -DMSO) δ: -79.00.

^{31}P{^{1}H} NMR (161.98 MHz, d_6 -DMSO) δ: 33.55.

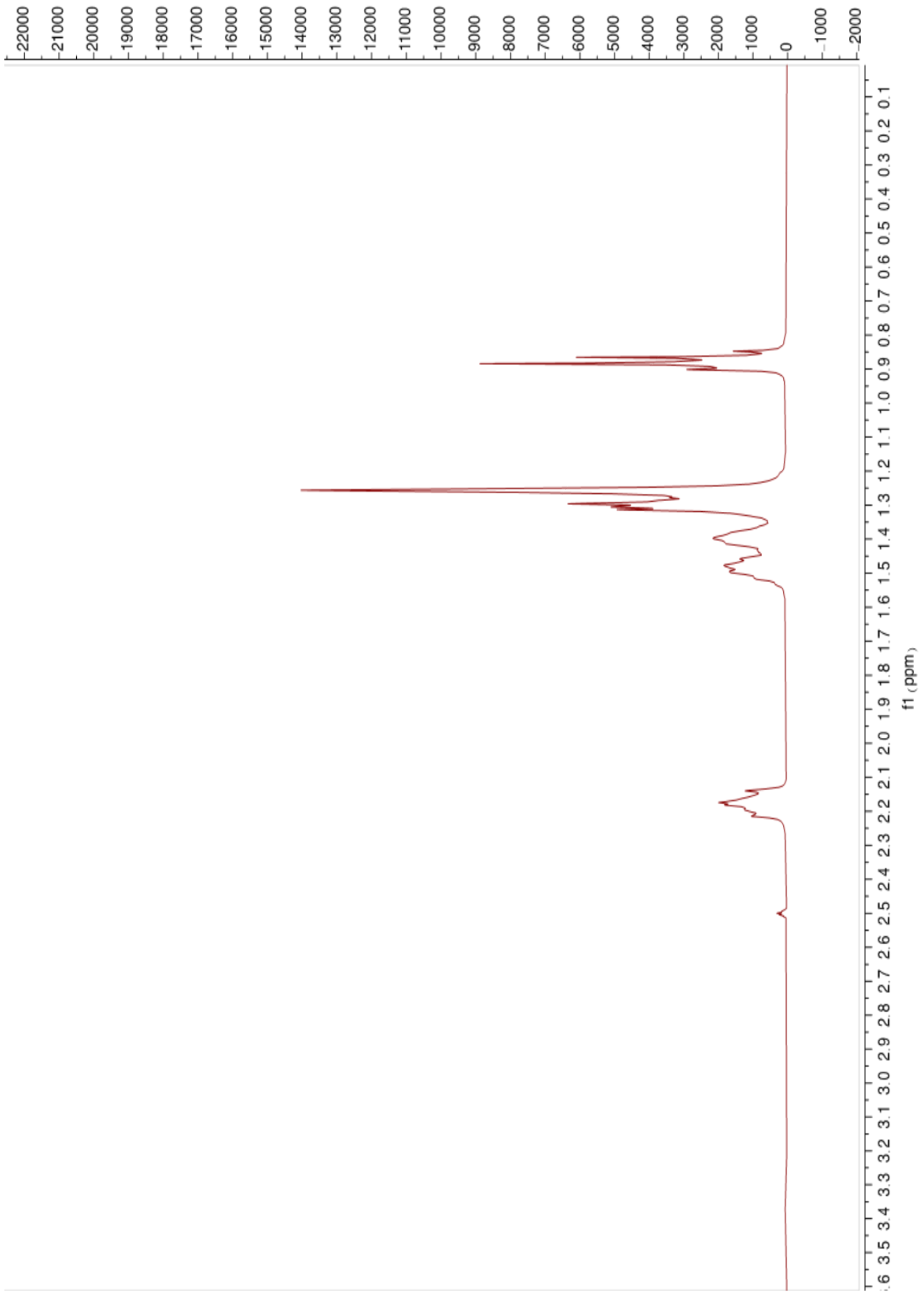

Figure S4. ^{1}H NMR spectrum (DMSO-d_6, 400.13 MHz, 298.15 K) of [$P_{666,14}$][TFSI].

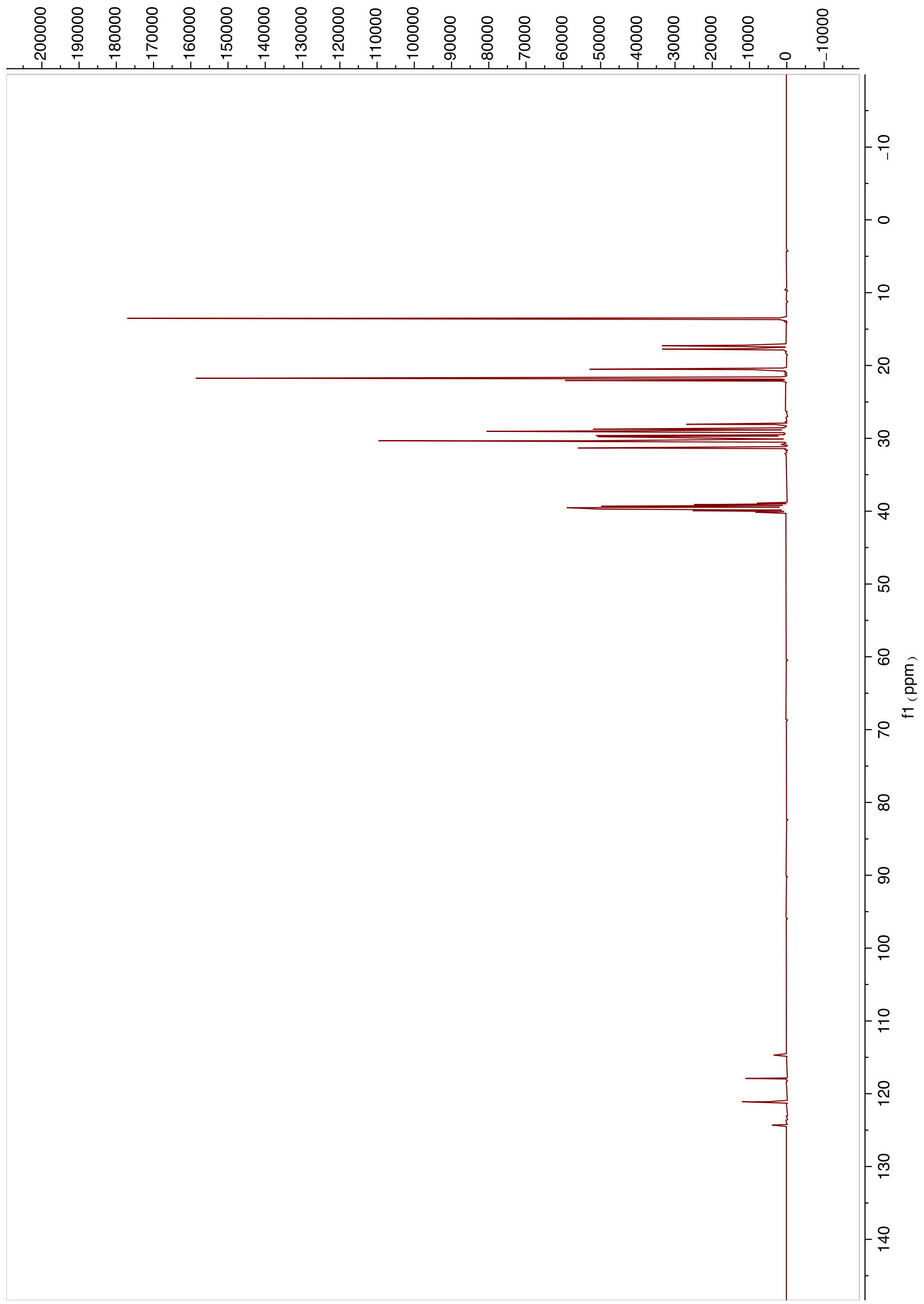

Figure S5. ^{13}C NMR spectrum (DMSO-d_6, 100.61 MHz, 298.15 K) of [$P_{666,14}$][TFSI].

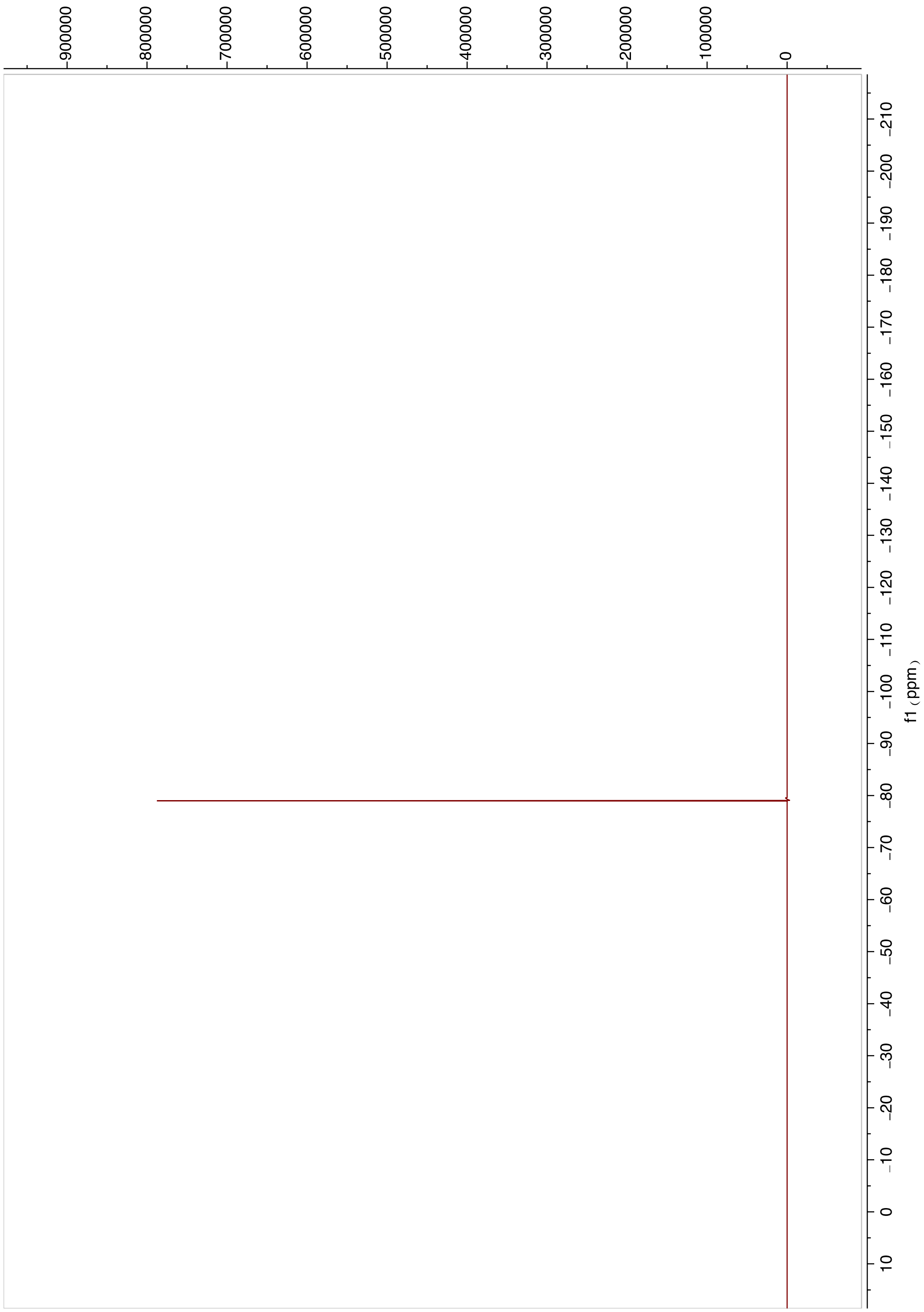

Figure S6. ^{19}F NMR spectrum (DMSO-d_6, 376.50 MHz, 298.15 K) of [$P_{666,14}$][TFSI].

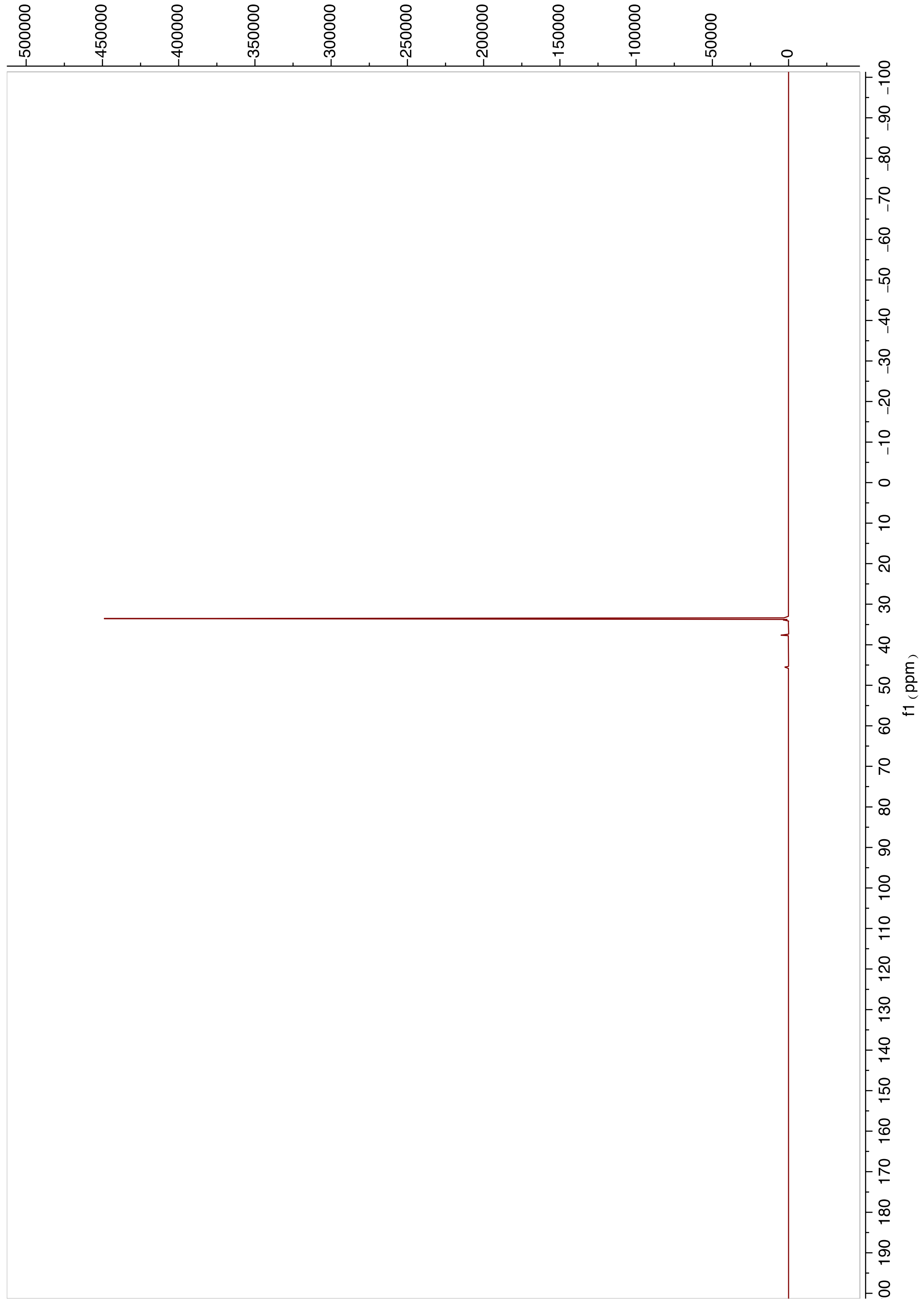

Figure S7. ^{31}P NMR spectrum of (DMSO-d_6, 161.98 MHz, 298.15 K) [$P_{666,14}$][TFSI].

[$P_{444,14}$]Cl . Tributyl(tetradecyl)phosphonium chloride, was provided by Solvay.

^{1}H NMR: δ: 0.85 (t, 3H), 0.92 (t, 9H), 1.25 (m, 20H), 1.45 (m, 16H), 2.07 (m, 8H).

^{13}C NMR: δ: 13.60 (P-(CH_2)$_3$-***CH_3***), 14.41 (P-(CH_2)$_{13}$-***CH_3***), 18.98 (d, 1$J_{C\text{-}P}$ = 47.26 Hz) (P-***CH_2***-(CH_2)$_2$-CH_3), 19.16 (d, 1$J_{C\text{-}P}$ = 47.26 Hz) (P-***CH_2***-(CH_2)$_{12}$-CH_3), 21.87 (d, 2$J_{C\text{-}P}$ = 5.03 Hz) (P-CH_2-***CH_2***-CH_2-CH_3), 23.37 (P-(CH_2)$_{12}$-***CH_2***-CH_3), 23.93 (d, 2$J_{C\text{-}P}$ = 5.03 Hz) (P-CH_2-***CH_2***-(CH_2)$_{11}$-CH_3), 24.49 (d, 3$J_{C\text{-}P}$ = 15.08 Hz) (P-(CH_2)$_2$-***CH_2***-(CH_2)$_{10}$-CH_3), 29.38 (P-(CH_2)$_3$-***CH_2***-(CH_2)$_9$-CH_3), 29.91 (P-(CH_2)$_4$-***CH_2***-(CH_2)$_8$-CH_3), 30.03 (P-(CH_2)$_5$-***CH_2***-(CH_2)$_7$-CH_3), 30.22 (P-(CH_2)$_6$-***CH_2***-(CH_2)$_6$-CH_3), 30.31 (P-(CH_2)$_7$-***CH_2***-(CH_2)$_5$-CH_3), 30.33 (P-(CH_2)$_8$-***CH_2***-(CH_2)$_4$-CH_3 and P-(CH_2)$_9$-***CH_2***-(CH_2)$_3$-CH_3), 30.37 (P-(CH_2)$_{10}$-***CH_2***-(CH_2)$_2$-CH_3 and P-(CH_2)$_{11}$-***CH_2***-CH_2-CH_3), 31.20 (d, 3$J_{C\text{-}P}$ = 15.08 Hz) (P-(CH_2)$_2$-***CH_2***-CH_3), 32.62 (P-(CH_2)$_{12}$-***CH_2***-CH_3).

^{31}P{^{1}H} NMR: δ: 33.56

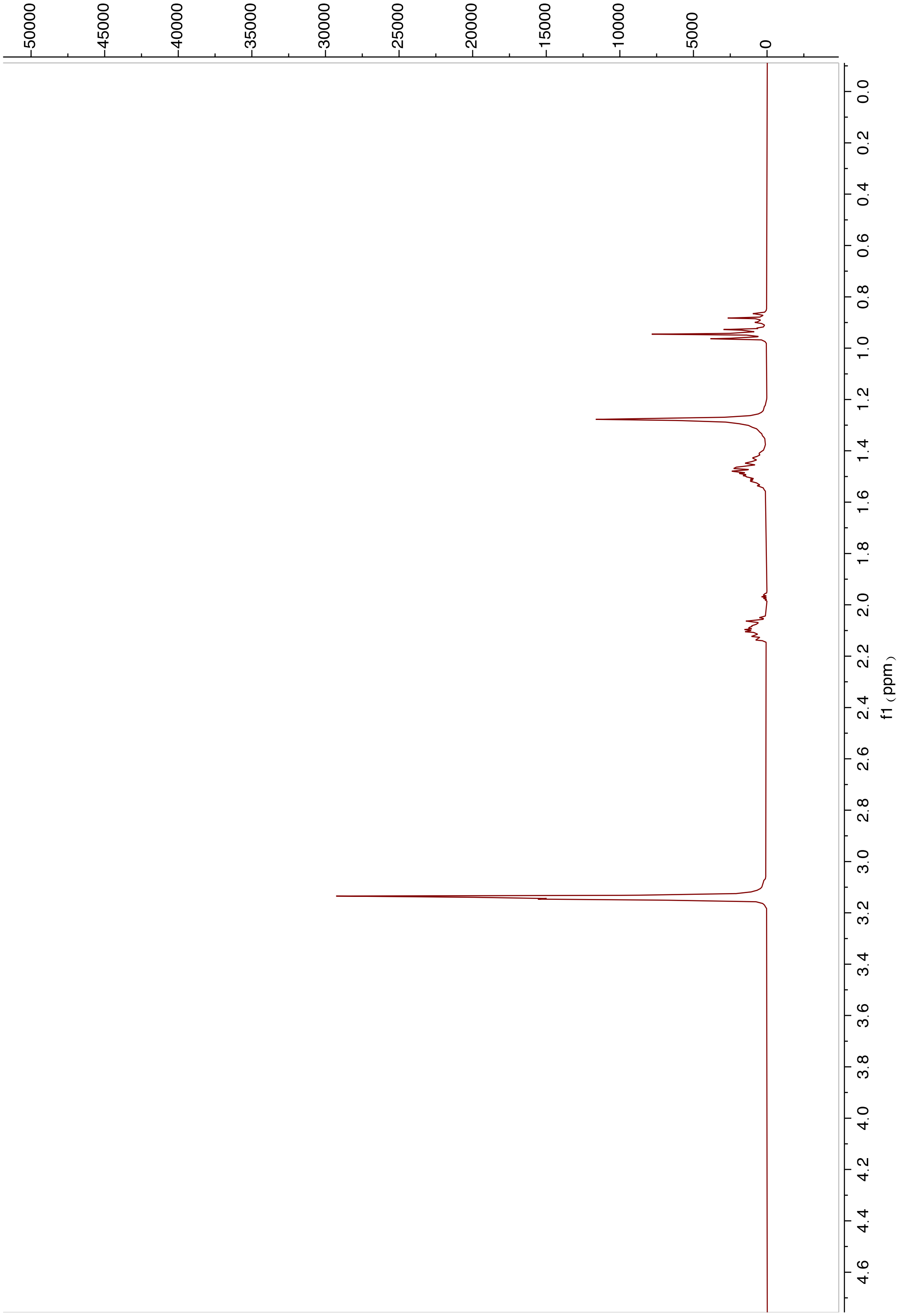

Figure S8. ^{1}H NMR spectrum (acetonitrile-d_3, 399.91 MHz, 300 K) of [$P_{444,14}$]Cl.

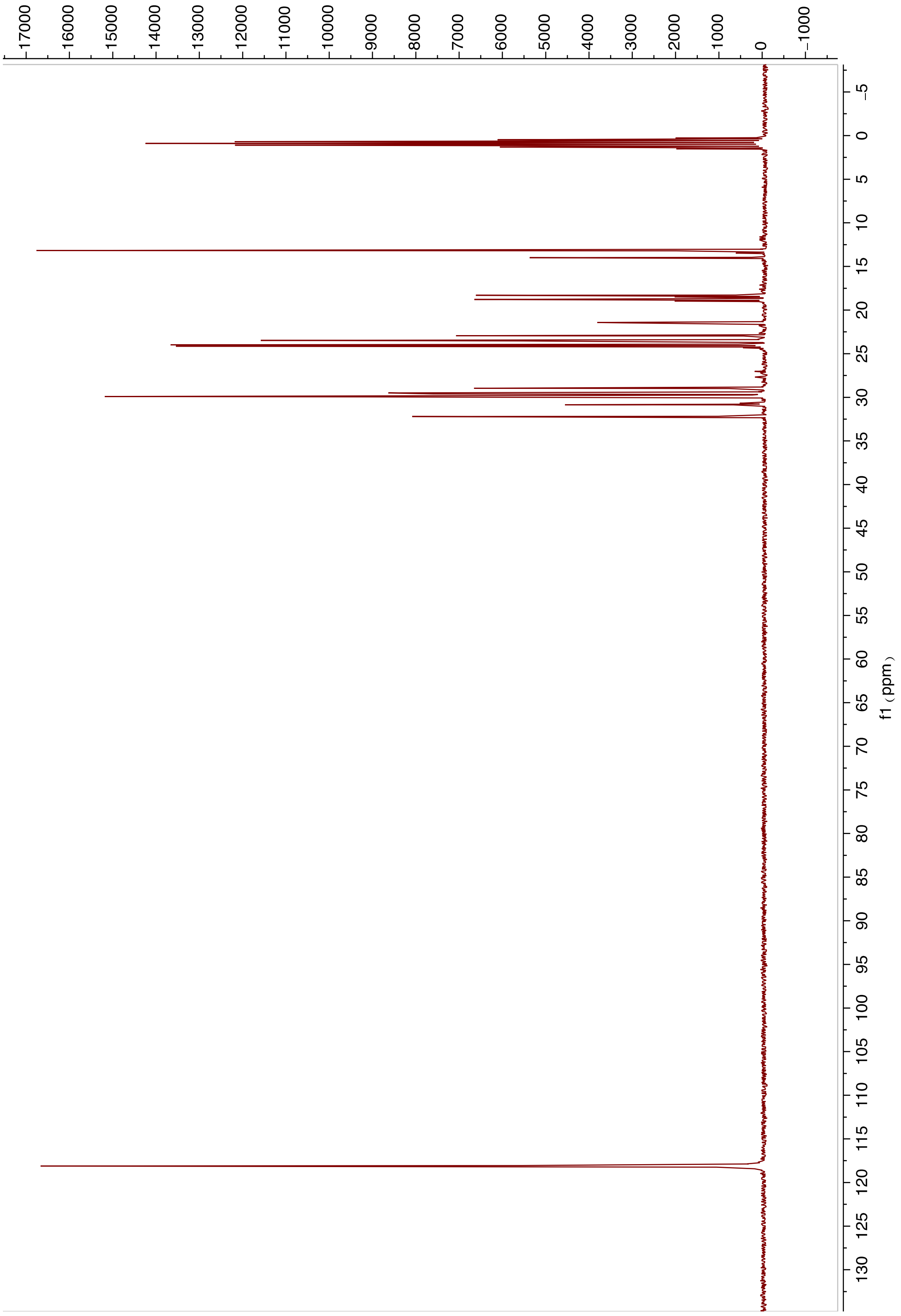

Figure S9. ^{13}C NMR spectrum (acetonitrile-d_3, 100.56 MHz, 301.4 K) of $[P_{444,14}]Cl$.

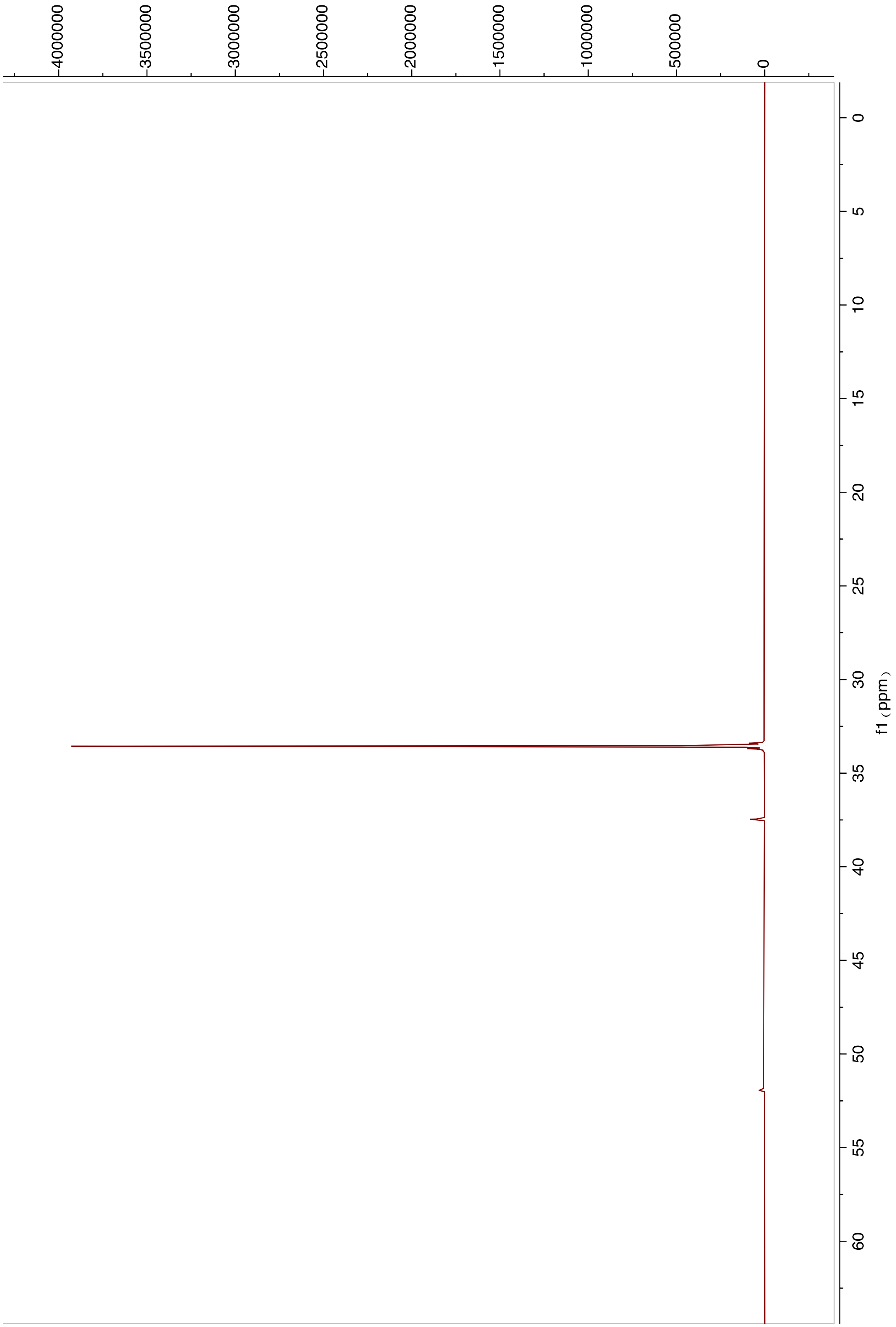

Figure S10. ^{31}P NMR spectrum (acetonitrile-*d*$_3$, 161.89 MHz, 300.1 K) of [$P_{444,14}$]Cl.

[$P_{444,14}$][TFSI]. Tributyl(tetradecyl)phosphonium chloride, [$P_{444,14}$]Cl (0.088 mol eq.) and lithium bis(trifluoromethanesulfon)imide Li[TFSI] (0.115 mol eq.) were separately dissolved in 200 cm^3 deionised water (18.2 MΩ.cm) (total 400 cm^3) and then combined in a round-bottomed flask (500 cm^3), resulting in the formation of a biphasic liquid system; the mixture was left to react (1 h, room temperature, 600 rpm). The aqueous layer was separated, and the organic layer was collected and washed, firstly with deionized water (18.2 MΩ.cm) (100 cm^3) and then dichloromethane, DCM (100 cm^3). Subsequent washes were performed with solution of Li[TFSI] in deionised water (18.2 MΩ.cm). Final three washes were performed with deionised water (18.2 MΩ.cm). The organic layer was washed 12 times in total. Subsequently, DCM was removed *via* rotary evaporation (30 min, 303.15 K) and the ionic liquid was dried under high vacuum (12h, 343.15 K, 10^{-2} mbar). XRF analysis confirmed chloride content was below the detectable limit. ^{1}H, ^{13}C, ^{19}F and ^{31}P NMR spectra of the IL were recorded in acetonitrile-d_3.

^{1}H NMR: δ: 0.89 (t, 3H), 0.95 (t, 9H), 1.28 (m, 20H), 1.47 (m, 16H), 2.06 (m, 8H).

^{13}C NMR: δ: (TFSI peaks 125.81, 122.62, 119.43, 116.24 (q, $1J_{C-F}$ = 320.82 Hz)), 13.58 (P-$(CH_2)_3$-***CH_3***), 14.47 (P-$(CH_2)_{13}$-***CH_3***), 19.01 (d, $1J_{C-P}$ = 48.27 Hz) (P-***CH_2***-$(CH_2)_2$-CH_3), 19.21 (d, $1J_{C-P}$ = 48.27 Hz)
(P-***CH_2***-$(CH_2)_{12}$-CH_3), 21.91 (d, $2J_{C-P}$ = 4.02 Hz) (P-CH_2-***CH_2***-CH_2-CH_3), 23.46 (P-$(CH_2)_{12}$-***CH_2***-CH_3), 23.95 (d, $2J_{C-P}$ = 4.02 Hz) (P-CH_2-***CH_2***-$(CH_2)_{11}$-CH_3), 24.54 (3d, J_{C-P} = 15.09 Hz) (P-$(CH_2)_2$-***CH_2***-$(CH_2)_{10}$-CH_3), 29.45 (P-$(CH_2)_3$-***CH_2***-$(CH_2)_9$-CH_3), 30.00 (P-$(CH_2)_4$-***CH_2***-$(CH_2)_8$-CH_3), 30.14 (P-$(CH_2)_5$-***CH_2***-$(CH_2)_7$-CH_3), 30.30 (P-$(CH_2)_6$-***CH_2***-$(CH_2)_6$-CH_3), 30.40 (P-$(CH_2)_7$-***CH_2***-$(CH_2)_5$-CH_3), 30.43 (P-$(CH_2)_8$-***CH_2***-$(CH_2)_4$-CH_3 and P-$(CH_2)_9$-***CH_2***-$(CH_2)_3$-CH_3), 30.47 (P-$(CH_2)_{10}$-***CH_2***-$(CH_2)_2$-CH_3 and P-$(CH_2)_{11}$-***CH_2***-CH_2-CH_3), 31.27 (d, $3J_{C-P}$ = 15.09 Hz) (P-$(CH_2)_2$-***CH_2***-CH_3), 32.72 (P-$(CH_2)_{12}$-***CH_2***-CH_3).

^{19}F NMR: δ: -80.01

^{31}P{^{1}H} NMR: δ: 33.64

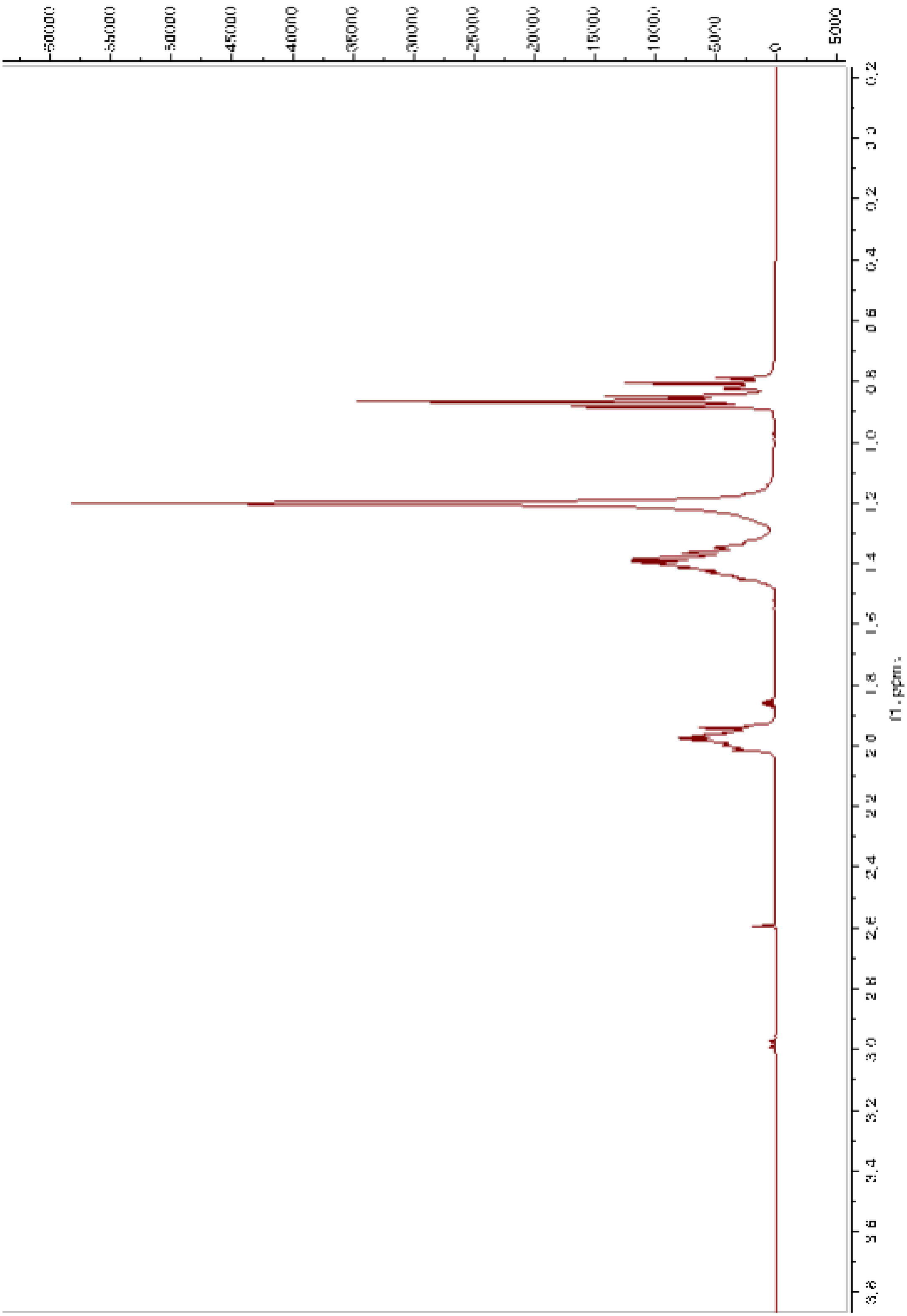

Figure S11. ^{1}H NMR spectrum (acetonitrile-d_3, 399.91 MHz, 299.9 K) of [$P_{444,14}$][TFSI].

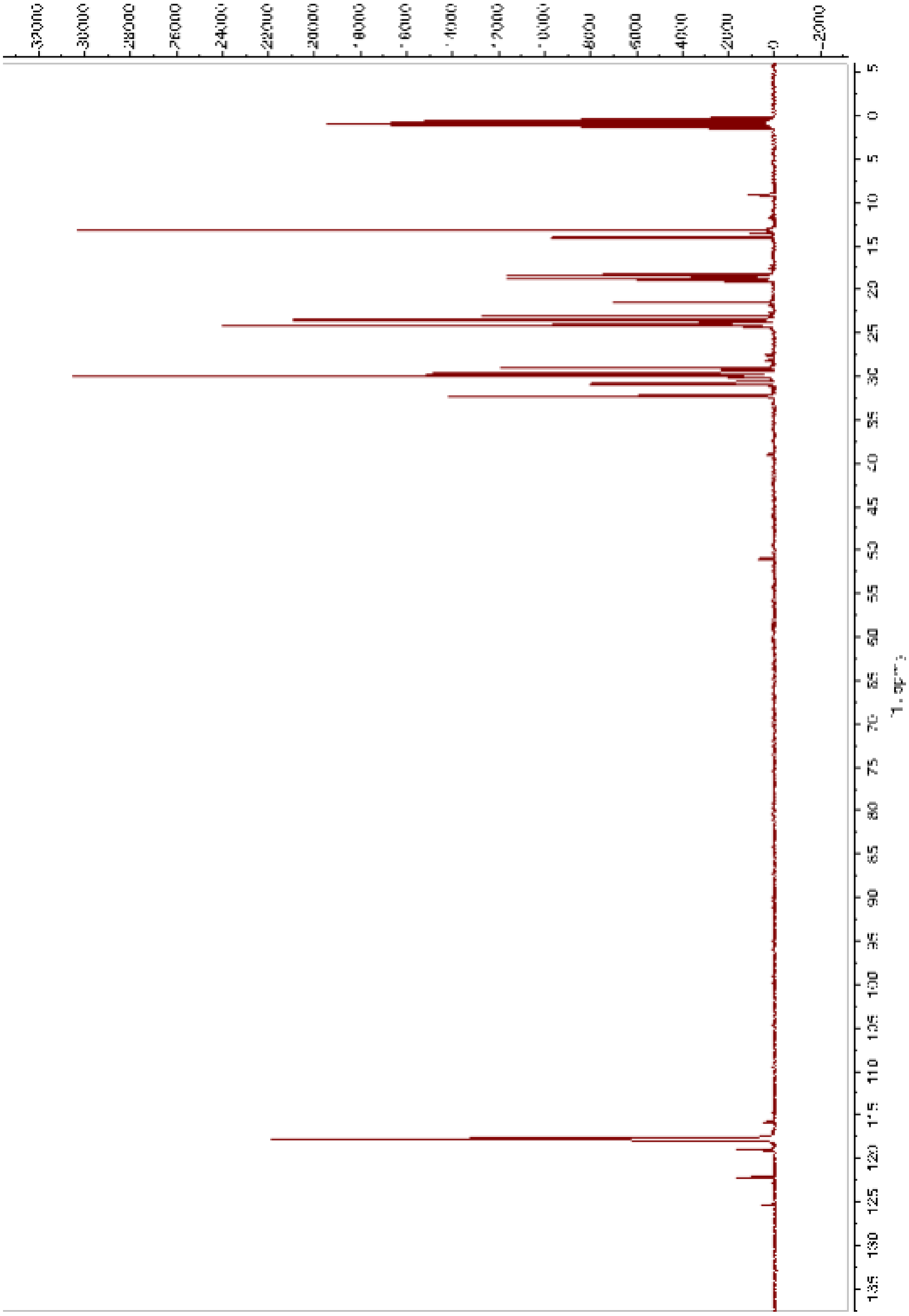

Figure S12. ^{13}C NMR spectrum (acetonitrile-d_3, 100.57 MHz, 299.9 K) of [$P_{444,14}$][TFSI].

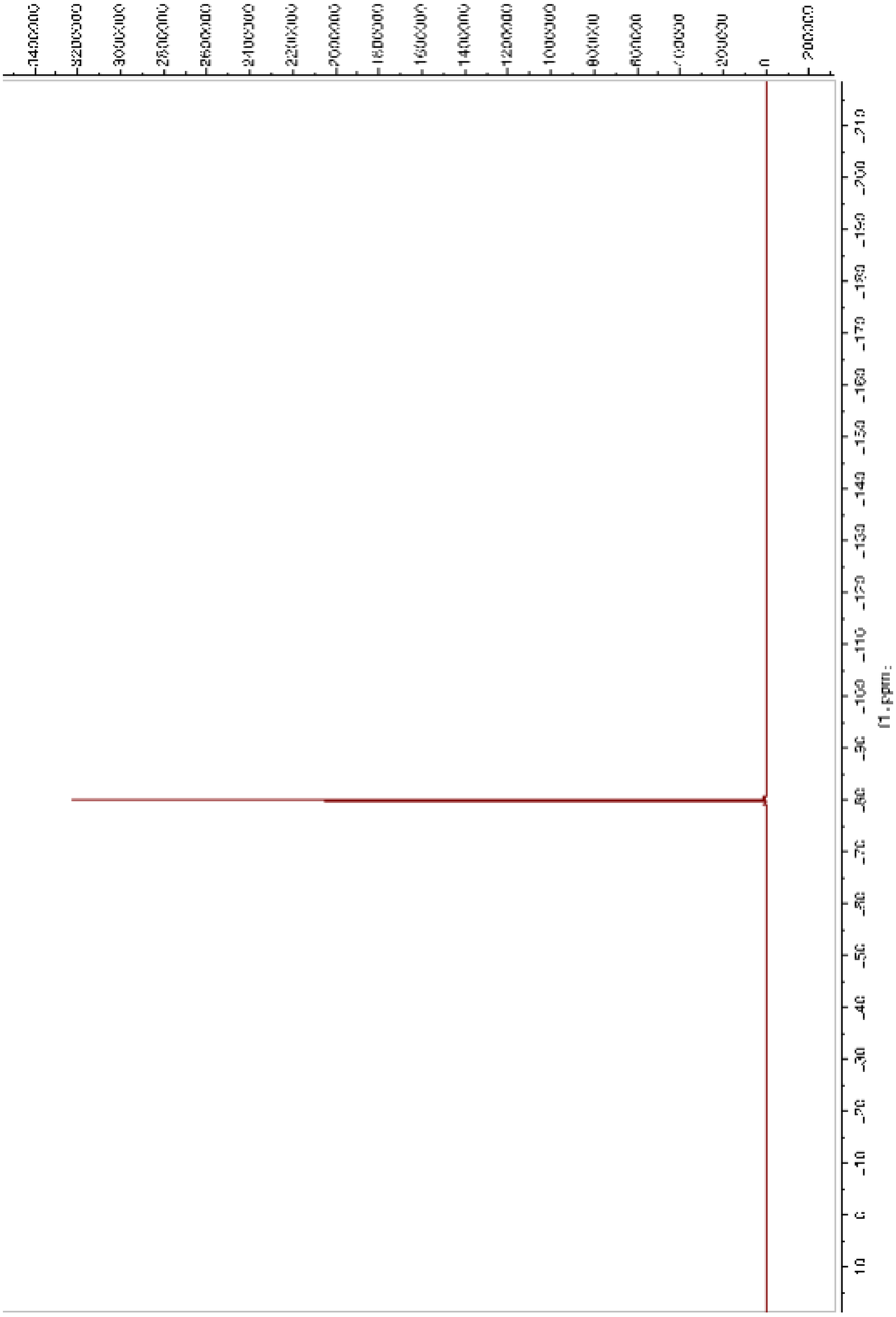

Figure S13. ^{19}F NMR spectrum (acetonitrile-d_3, 376.29 MHz, 299.2 K) of [$P_{444,14}$][TFSI].

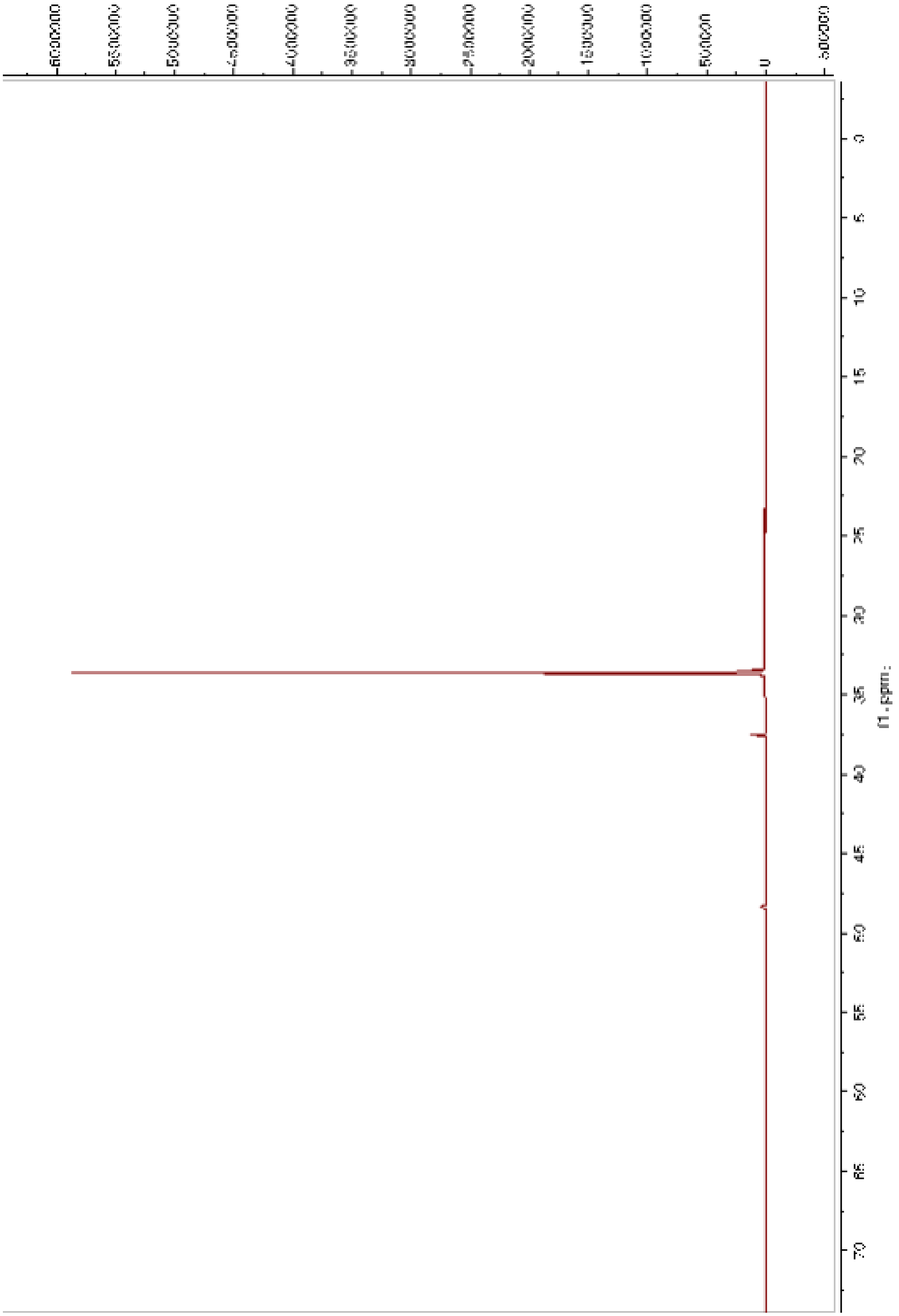

Figure S14. ^{31}P NMR spectrum (acetonitrile-d_3, 161.86 MHz, 300 K) of [$P_{444,14}$][TFSI].

[$P_{666,2}$]TFSI]. Trihexylphosphine (60g) was charged to an autoclave glass reactor under a dinitrogen atmosphere and placed into the stainless-steel cage holder, and quickly attached to the cover plate. A gas cylinder containing chloroethane was connected to the autoclave through the gas inlet tube. The gas and inlet valves were opened after the set up was leak tested and the gas flow was maintained at approximately 2 cm^3min^{-1}. The reaction mixture was slowly heated over several hours in an oil bath until the temperature inside of the reactor reached 418.15 K. The pressure in the reactor was built up by an excess of chloroalkane and did not exceed 4 bar with increasing temperature. The gas inlet was closed after approximately one hour and the reaction was maintained (4h, 418.15 K). After the reaction was cooled down to room temperature the pressure was released. The content of the reactor was the transferred to a round-bottomed flask (100 cm^3) and dried *in vacuo* (48h, 338.15 K). The synthesised trihexyl(ethyl)phosphonium chloride, [$P_{666,2}$]Cl (1.000 mol eq.) was dissolved in dichloromethane, DCM (100 cm^3) while stirring mildly in an Erlenmeyer flask (250 cm^3). Lithium bis(trifluoromethanesulfon)imide Li[TFSI] (1.020 mol eq.) was separately dissolved in 20 cm^3 deionised water (18.2 MΩ.cm) and then combined resulting in the formation of a biphasic liquid system; the mixture was left to react (1.5 h, room temperature). The aqueous layer was separated, and the organic layer was collected and washed, firstly with dichloromethane, DCM (3 x 30 cm^3). The combined washes were then washed with deionized water (18.2 MΩ.cm) (15 x 30 cm^3). The dichloromethane, DCM solution was dried by addition of anhydrous sodium sulfate (*ca.* 10 g), then isolated *via* vacuum filtration of the Na_2SO_4 (P3 glass sintered funnel with Celite). The filtrate was transferred to a round bottom flask, and dichloromethane, DCM was removed *in vacuo* by rotary evaporation at (303.15 K) and further under high vacuum (48h, 343.15 K, 10^{-2} mbar), leaving clear liquid product. XRF analysis confirmed chloride content was below the detectable limit. ^{1}H, ^{13}C, ^{19}F and ^{31}P NMR spectra of the IL were recorded in acetonitrile-d_3.

^{1}H NMR: δ: 0.91 (t, 9H), 1.15 (m, 3H), 1.32 (m, 12H), 1.44 (m, 12H), 2.06 (m, 8H).

^{13}C NMR: (TFSI peaks 125.84, 122.65, 119.45, 116.27 (q, $1J_{C-F}$ = 320.82 Hz)), 5.91 (d, $2J_{C-P}$ = 6.03 Hz) (P-CH_2-***CH_3***), 13.02 (d, $1J_{C-P}$ = 50.28 Hz) (P-***CH_2***-CH_3), 14.30 (P-$(CH_2)_5$-***CH_3***), 18.87 (d, $1J_{C-P}$ = 47.26 Hz) (P-***CH_2***-$(CH_2)_4$-CH_3), 21.91 (d, $2J_{C-P}$ = 5.03 Hz) (P-CH_2-***CH_2***-$(CH_2)_3$-CH_3), 23.07 (P-$(CH_2)_4$-***CH_2***-CH_3), 30.99 (d, $3J_{C-P}$ = 15.08 Hz) (P-$(CH_2)_2$-***CH_2***-$(CH_2)_2$-CH_3), 31.68 (P-$(CH_2)_3$-***CH_2***-CH_2-CH_3).

^{19}F NMR: δ: -80.01

^{31}P{^{1}H} NMR: δ: 35.30

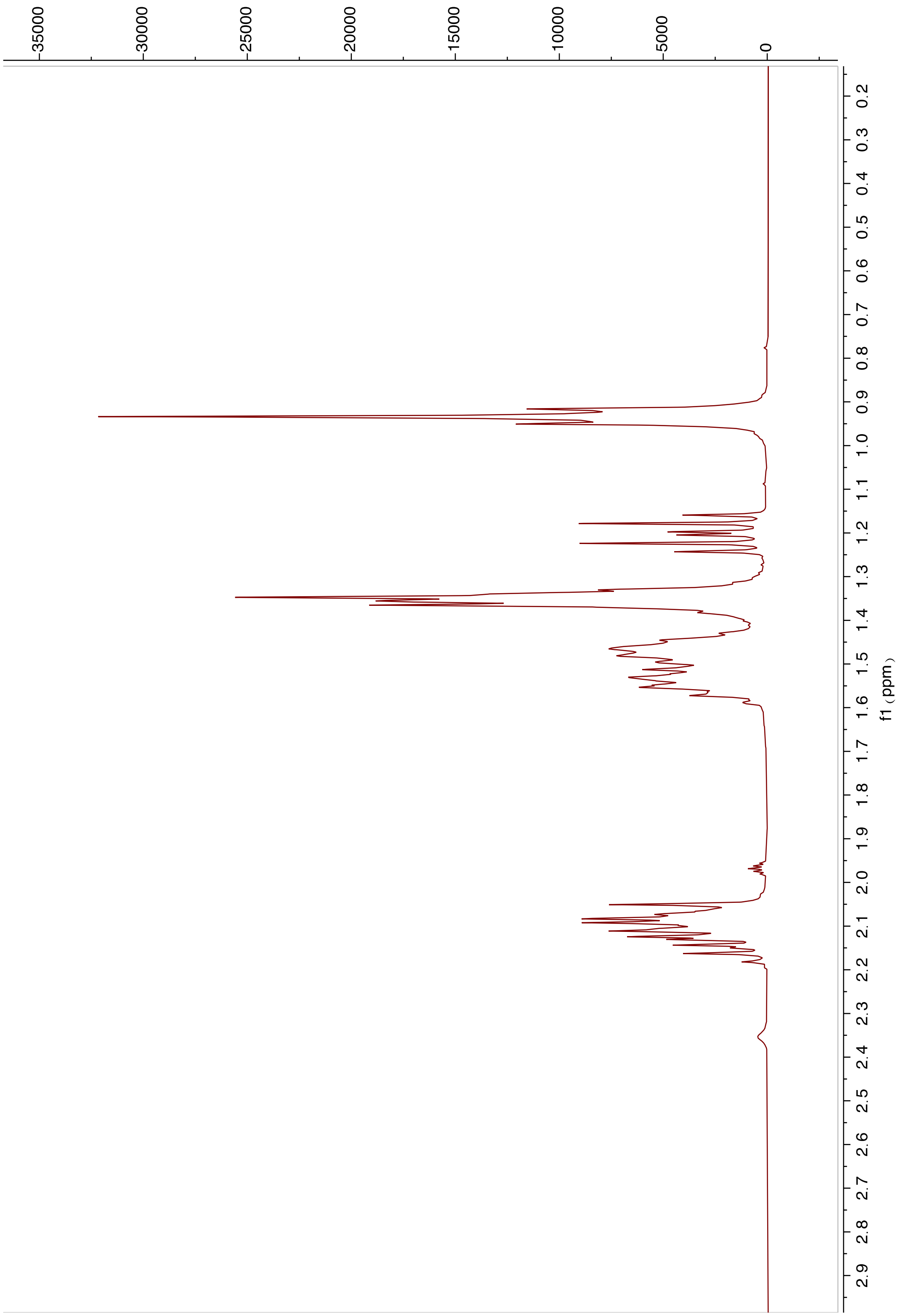

Figure S15. ^{1}H NMR spectrum (acetonitrile-d_3, 399.1 MHz, 300.5 K) of [$P_{666,2}$][TFSI].

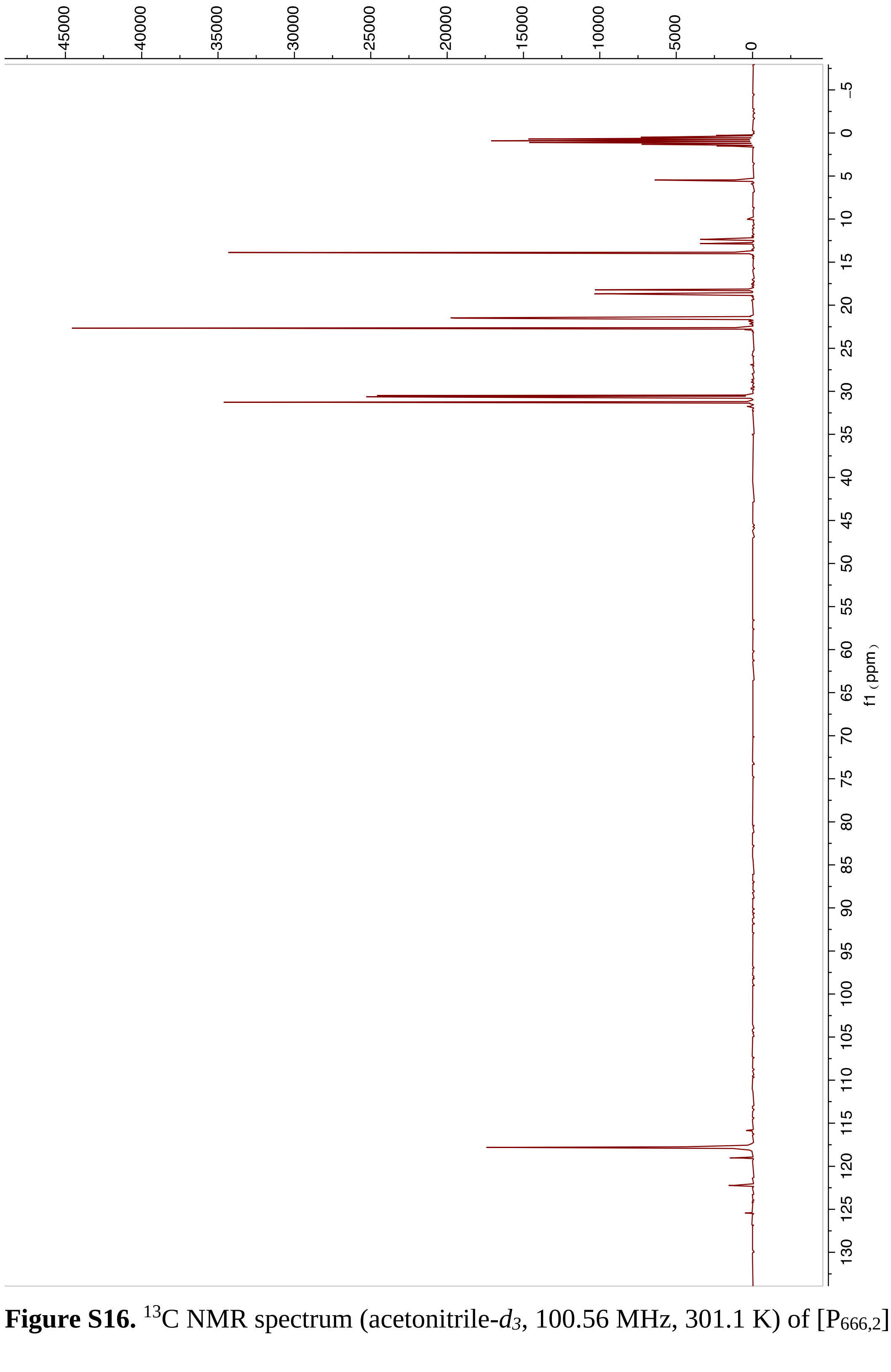

Figure S16. ^{13}C NMR spectrum (acetonitrile-d_3, 100.56 MHz, 301.1 K) of [$P_{666,2}$][TFSI].

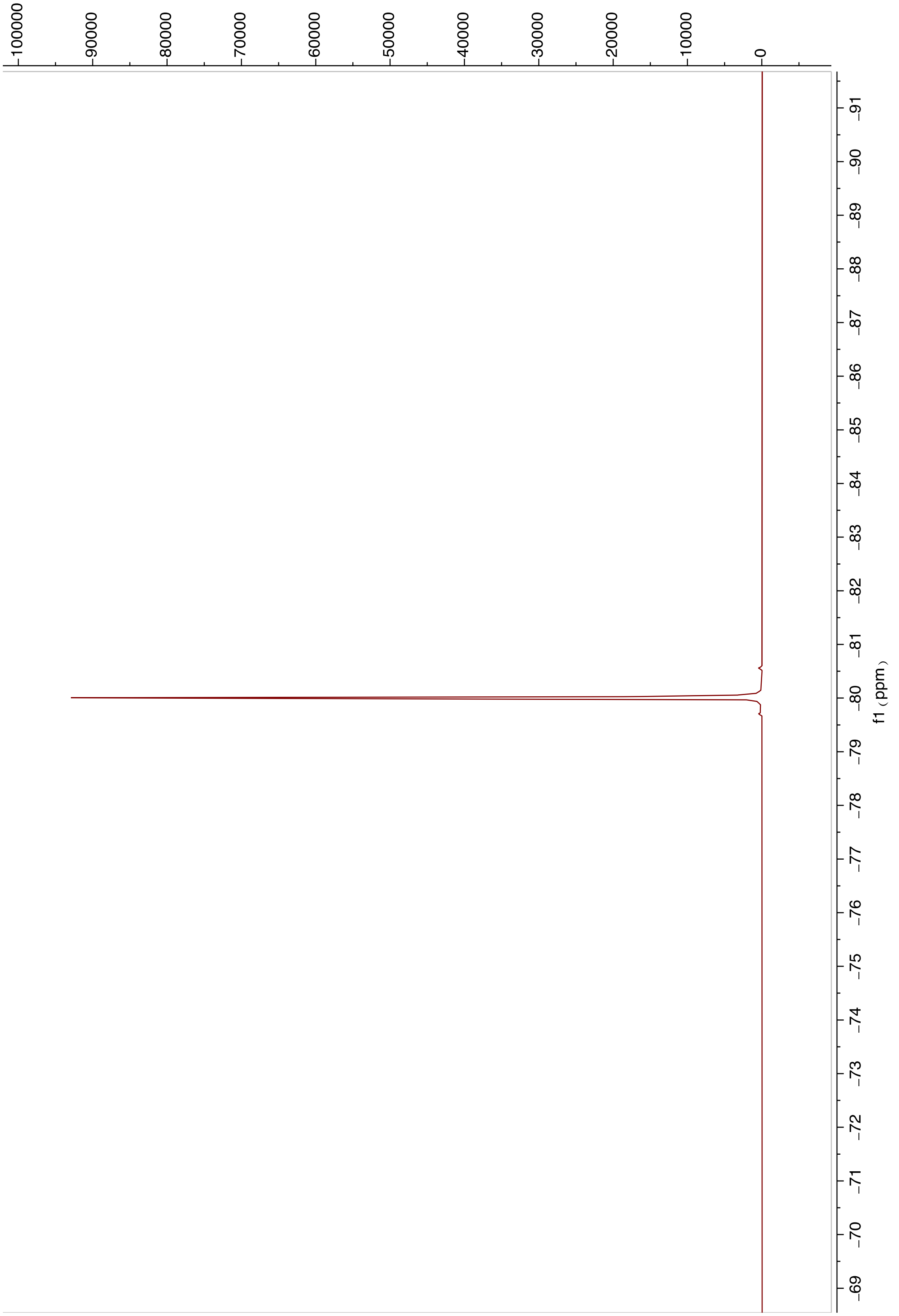

Figure S17. ^{19}F NMR spectrum (acetonitrile-d_3, 376.29 MHz, 300.2 K) of [$P_{666,2}$][TFSI].

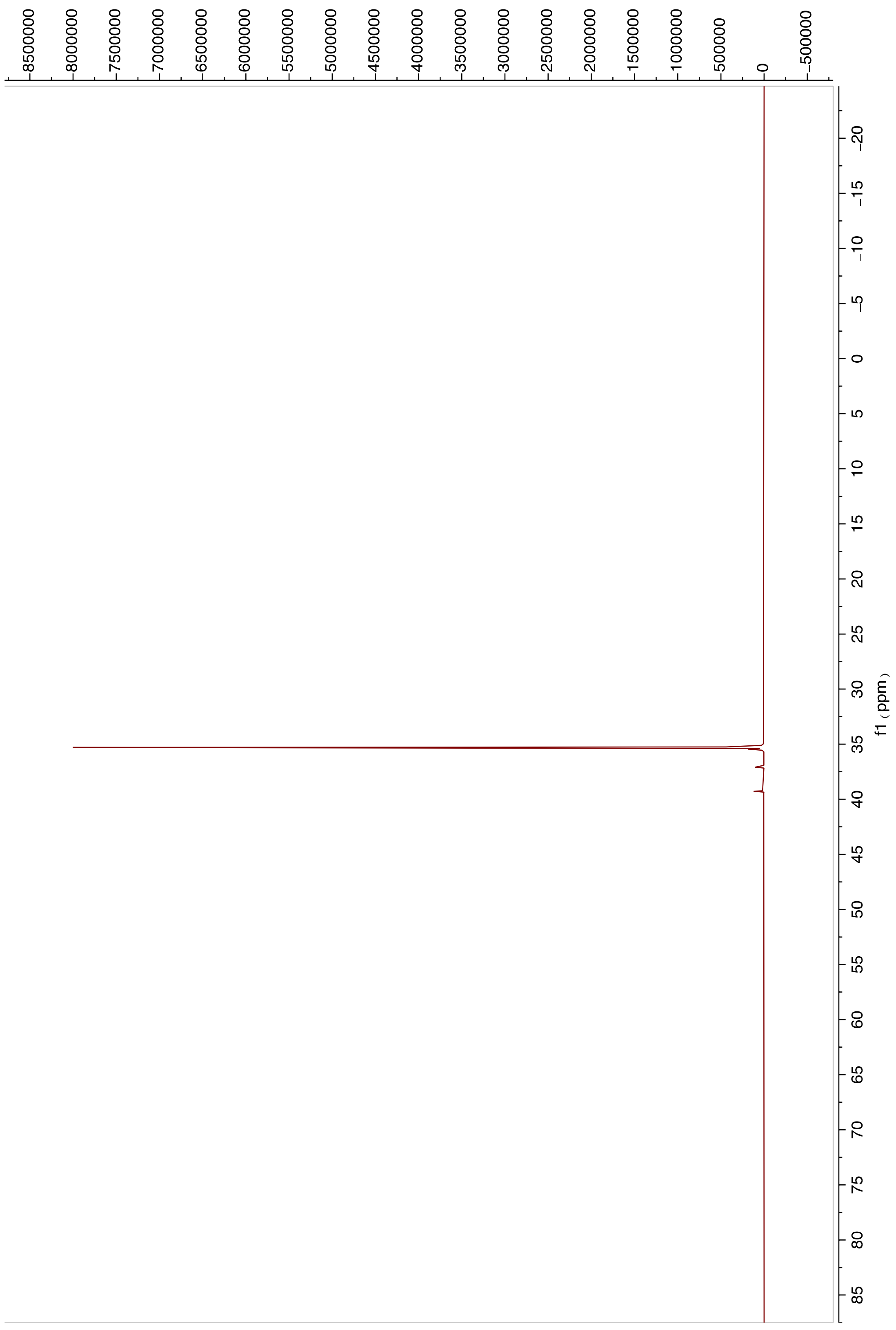

Figure S18. ^{31}P NMR spectrum (acetonitrile-d_3, 161.88 MHz, 300 K) of [$P_{666,2}$][TFSI].

[$P_{666,6}$]Cl. Trihexylphosphine (1.000 mol eq.) and 1-chlorohexane ($C_6H_{13}Cl$) (1.100 mol eq.) were placed in a round-bottomed flask (50 cm^3). The mixture was stirred and heated under a reflux condenser connected to a Schlenk line flushed with dinitrogen (12h, 418.15 K). In order to remove the excess of starting $C_6H_{13}Cl$, the purification step involved dissolution of the product in hexane, followed by extraction with water. The product was the dried at *in vacuo* (48 h, 338.15 K). ^{1}H, ^{13}C, and ^{31}P NMR spectra of the IL were recorded in acetonitrile-d_3.

^{1}H NMR: δ: 0.75 (t, 12H), 1.17 (m, 16H), 1.29 (m, 8H), 1.41 (m, 8H), 2.34 (m, 8H).

^{13}C NMR: δ: 14.01 (P-$(CH_2)_5$-***CH_3***), 19.05 (d, $1J_{C-P}$ = 47.26 Hz) (P-***CH_2***-$(CH_2)_4$-CH_3), 21.76 (d, $2J_{C-P}$ = 4.02 Hz) (P-CH_2-***CH_2***-$(CH_2)_3$-CH_3), 22.59 (P-$(CH_2)_4$-***CH_2***-CH_3), 30.67 (d, $3J_{C-P}$ = 15.08 Hz) (P-$(CH_2)_2$-***CH_2***-$(CH_2)_2$-CH_3), 31.27 (P-$(CH_2)_3$-***CH_2***-CH_2-CH_3).

^{31}P{^{1}H} NMR: δ: 33.04

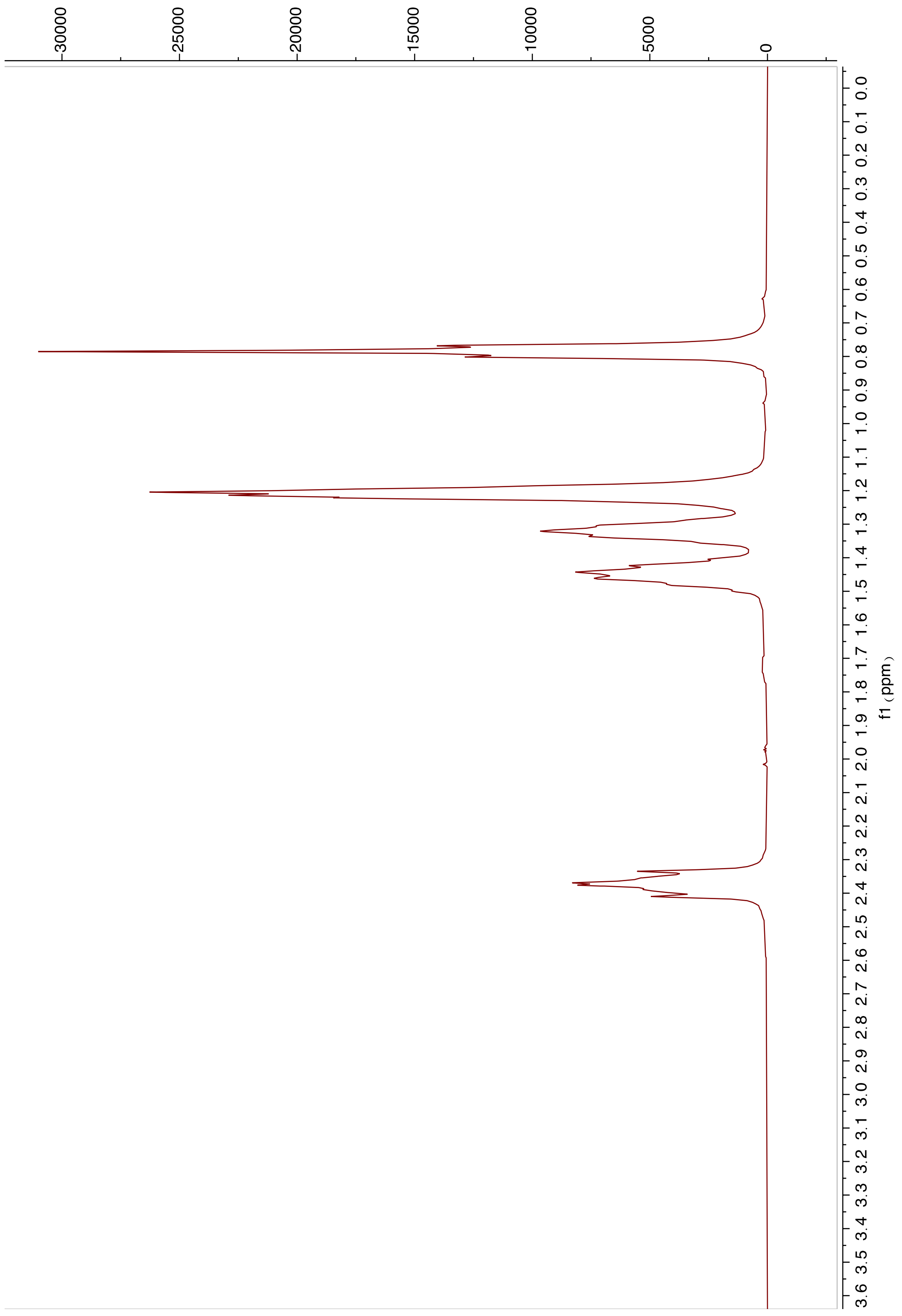

Figure S19. ^{1}H NMR spectrum (acetonitrile-d_3, 399.90 MHz, 300.2 K) of [$P_{666,6}$]Cl.

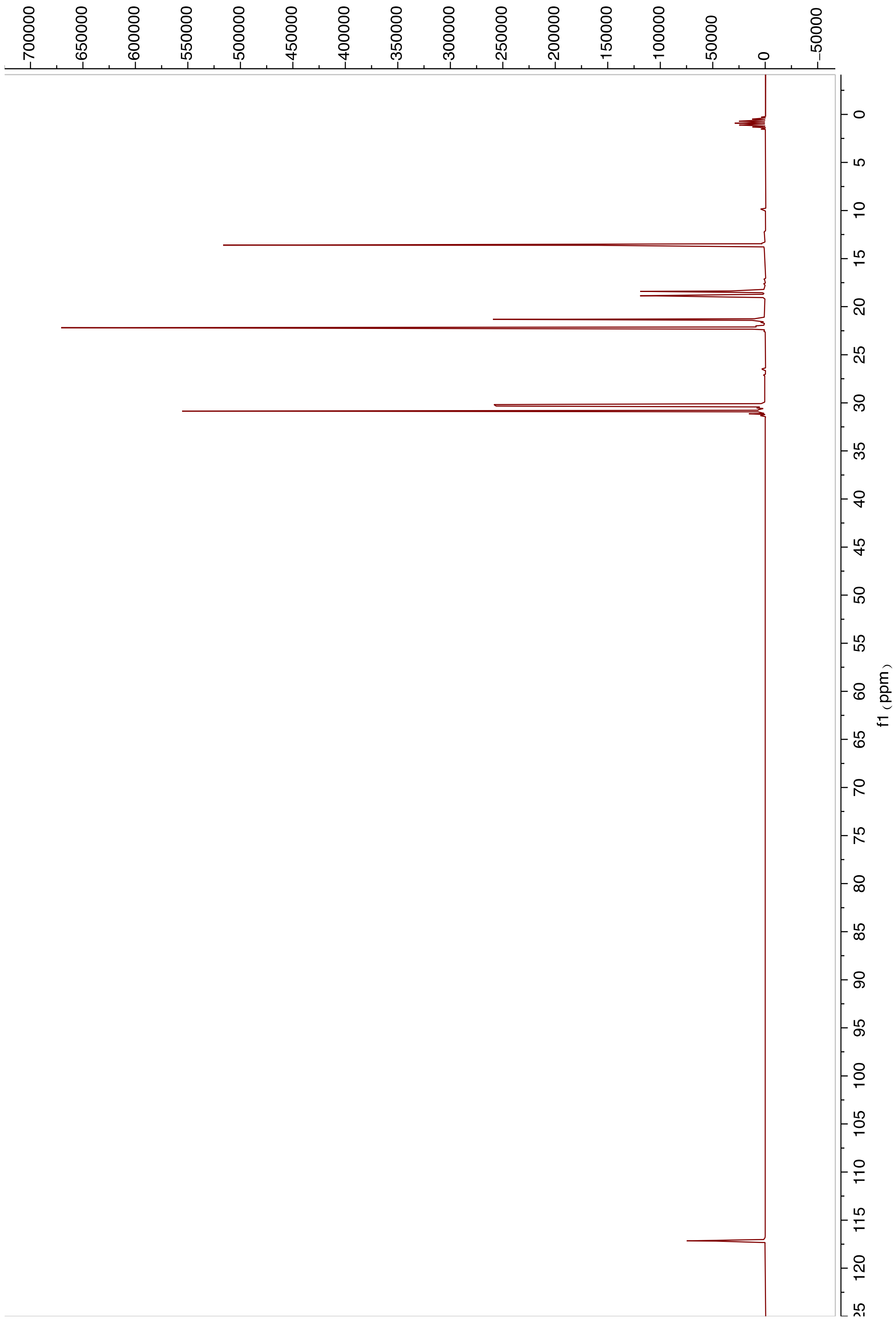

Figure S20. ^{13}C NMR spectrum (acetonitrile-d_3, 100.55 MHz, 301.1 K) of $[P_{666,6}]Cl$.

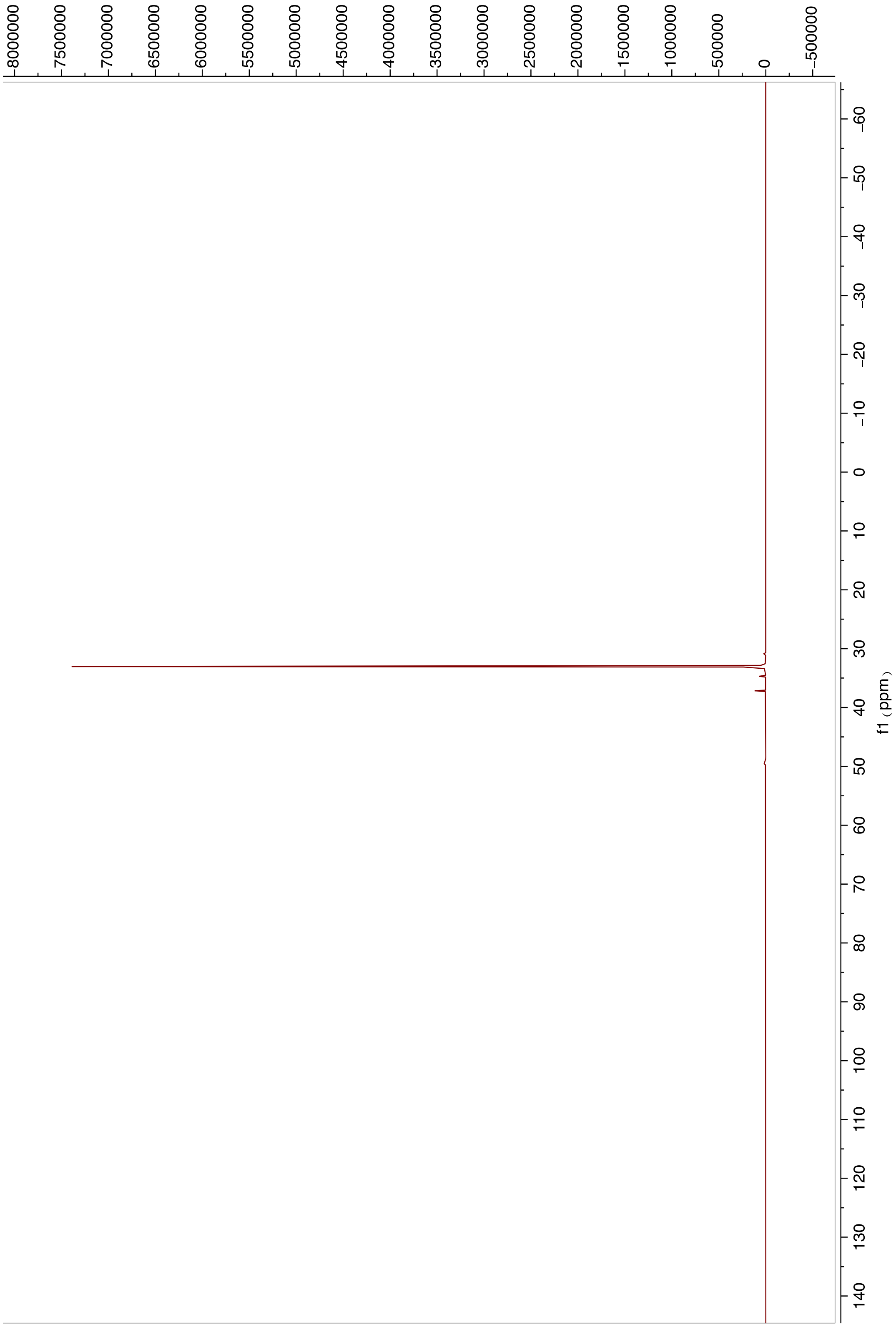

Figure S21. ^{31}P NMR spectrum (acetonitrile-d_3, 161.89 MHz, 300 K) of [$P_{666,6}$]Cl.

[$P_{666,6}$]TFSI]. Tetrahexylphosphonium chloride, [$P_{666,6}$]Cl (0.009 mol eq.) and lithium bis(trifluoromethanesulfon)imide Li[TFSI] (0.012 mol eq.) were separately dissolved in 25 cm^3 deionised water (18.2 MΩ.cm) (total 50 cm^3) and then combined in a round-bottomed flask (250 cm^3), resulting in the formation of a biphasic liquid system; the mixture was left to react (1 h, room temperature, 600 rpm). The aqueous layer was separated, and the organic layer was collected and washed, firstly with deionized water (18.2 MΩ.cm) (10 cm^3) and then dichloromethane, DCM (10 cm^3). Subsequent washes were performed with solution of Li[TFSI] in deionised water (18.2 MΩ.cm). Final three washes were performed with deionised water (18.2 MΩ.cm). The organic layer was washed 10 times in total. Subsequently, DCM was removed *via* rotary evaporation (30 min, 308.15 K) and the ionic liquid was dried under high vacuum (12h, 343.15 K, 10^{-2} mbar). XRF analysis confirmed chloride content was below the detectable limit. ^{1}H, ^{13}C, ^{19}F and ^{31}P NMR spectra of the IL were recorded in acetonitrile-d_3.

^{1}H NMR: δ: 0.91 (t, 12H), 1.32 (m, 16H), 1.43 (m, 16H), 2.05 (m, 8H).

^{13}C NMR: δ: (TFSI peaks 125.83, 122.64, 119.45, 116.26 (q, $1J_{C\text{-}F}$ = 320.82 Hz), 14.31 (P-$(CH_2)_5$-***CH_3***), 19.24 (d, $1J_{C\text{-}P}$ = 47.27 Hz) (P-***CH_2***-$(CH_2)_4$-CH_3), 21.95 (d, $2J_{C\text{-}P}$ = 5.03 Hz) (P-CH_2-***CH_2***-$(CH_2)_3$-CH_3), 23.07 (P-$(CH_2)_4$-***CH_2***-CH_3), 30.98 (d, $3J_{C\text{-}P}$ = 15.09 Hz) (P-$(CH_2)_2$-***CH_2***-$(CH_2)_2$-CH_3), 31.67 (P-$(CH_2)_3$-***CH_2***-CH_2-CH_3).

^{19}F NMR: δ: -79.97

^{31}P{^{1}H} NMR: δ: 33.50

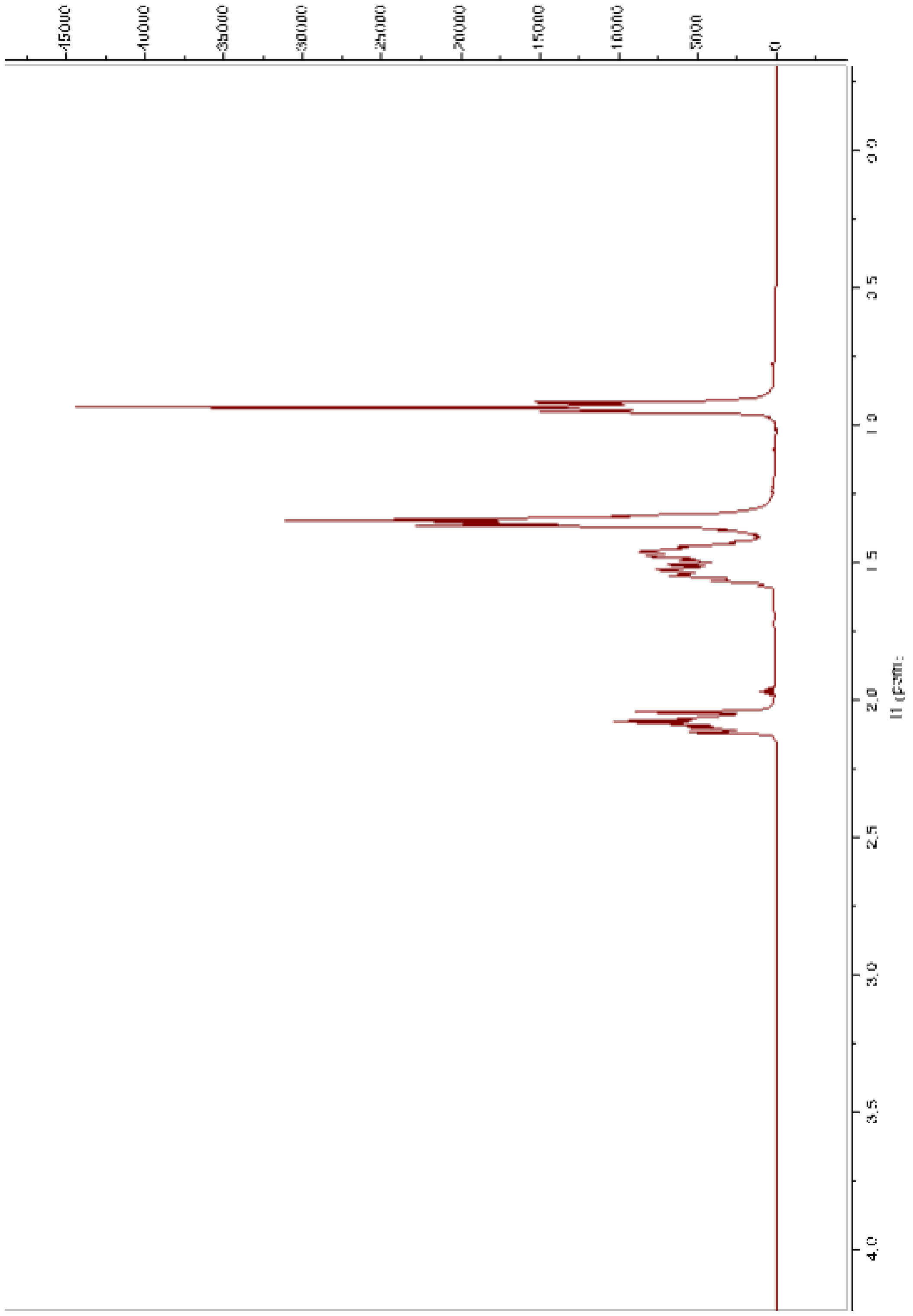

Figure S22. ^{1}H NMR spectrum (acetonitrile-d_3, 399.91 MHz, 300.9 K) of [$P_{666,6}$]TFSI].

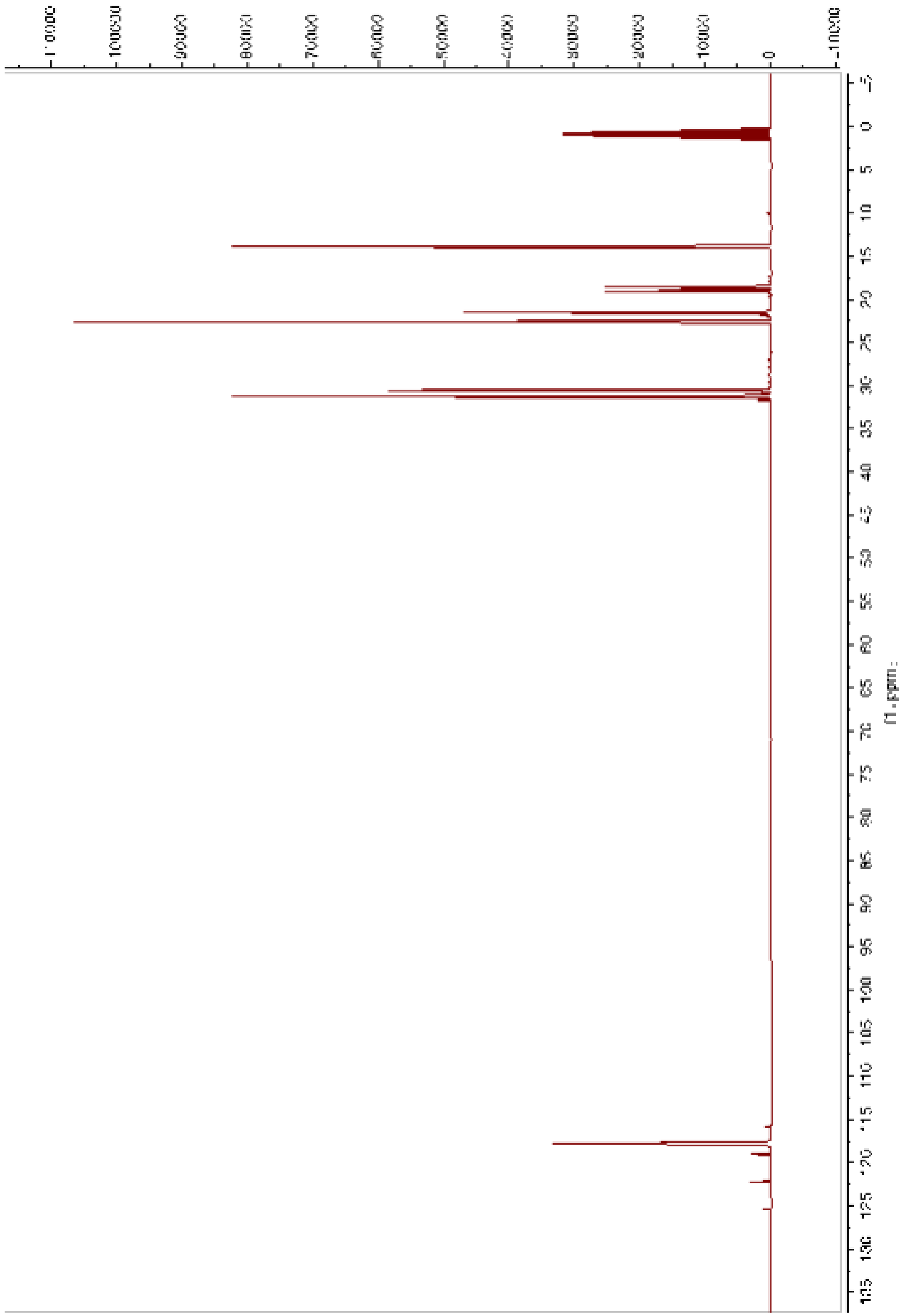

Figure S23. ^{13}C NMR spectrum (acetonitrile-d_3, 100.57 MHz, 300.5 K) of [$P_{666,6}$]TFSI].

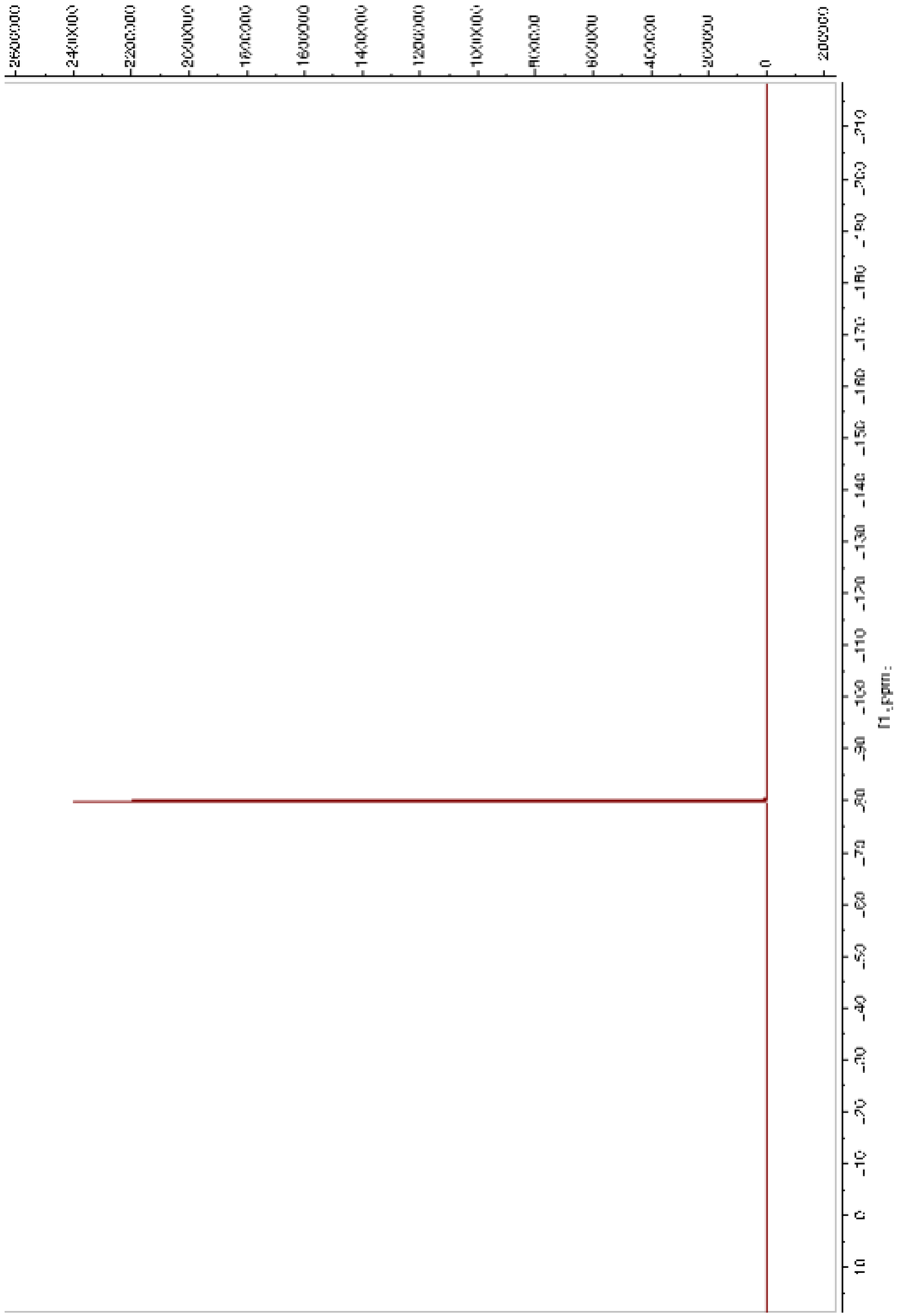

Figure S24. ^{19}F NMR spectrum (acetonitrile-*d*$_3$, 376.29 MHz, 299.7 K) of [$P_{666,6}$]TFSI].

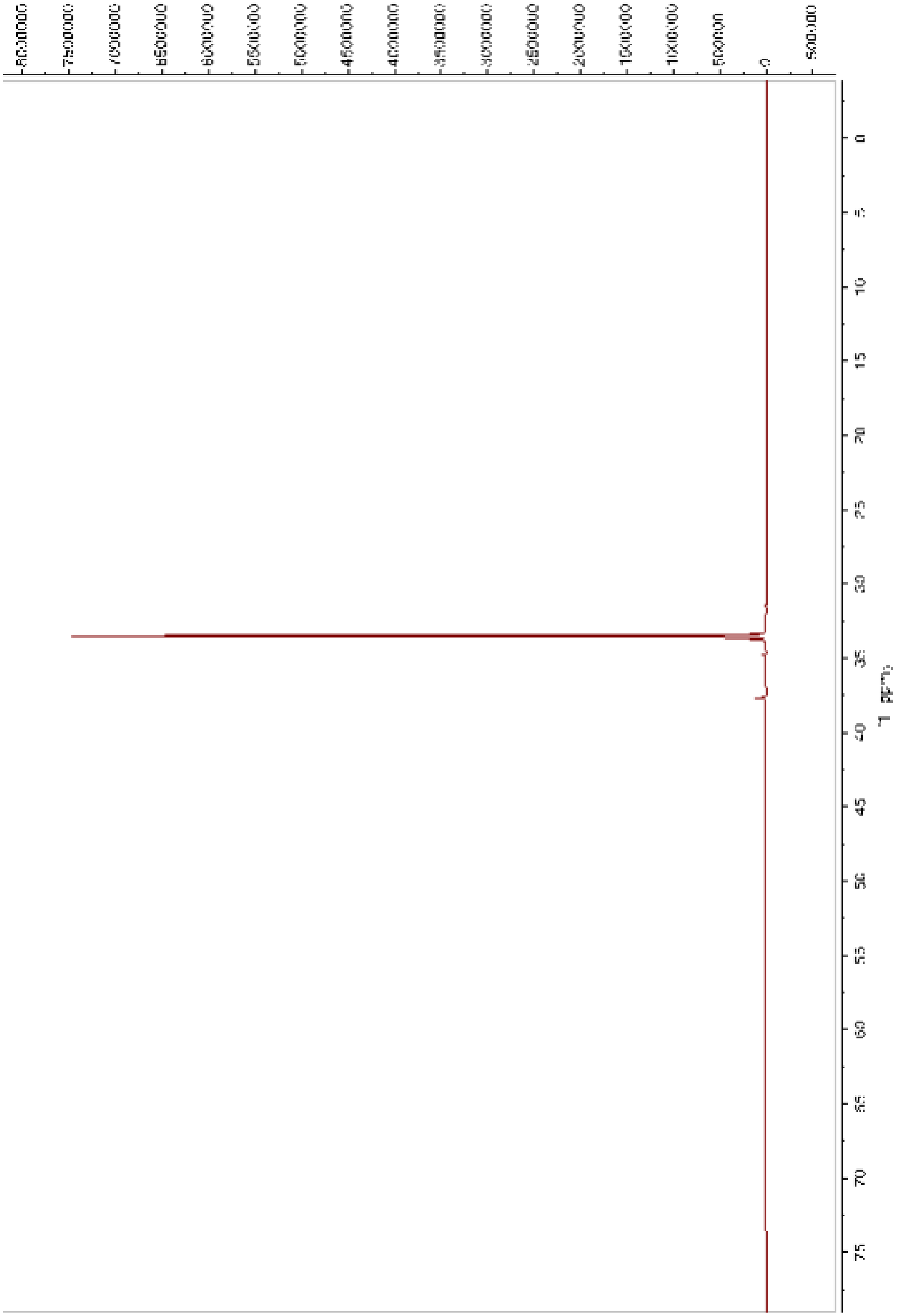

Figure S25. ^{31}P NMR spectrum (acetonitrile-d_3, 161.89 MHz, 300.2 K) of [$P_{666,6}$]TFSI].

[$P_{666,8}$]TFSI]. Trihexylphosphine (1.000 mol eq.) and 1-chlorooctane ($C_8H_{17}Cl$) (1.100 mol eq.) were placed in a round-bottomed flask (50 cm^3). The mixture was stirred and heated under a reflux condenser connected to a Schlenk line flushed with dinitrogen (14h, 418.15 K). In order to remove the excess of starting $C_8H_{17}Cl$, the purification step involved dissolution of the product in hexane, followed by extraction with water. The synthesised trihexyl(octyl)phosphonium chloride, [P_{6668}]Cl (1.000 mol eq.) was dissolved in dichloromethane, DCM (100 cm^3) while stirring mildly in an Erlenmeyer flask (250 cm^3). Lithium bis(trifluoromethanesulfon)imide Li[TFSI] (1.020 mol eq.) was separately dissolved in 20 cm^3 deionised water (18.2 MΩ.cm) and then combined resulting in the formation of a biphasic liquid system; the mixture was left to react (1.5 h, room temperature). The aqueous layer was separated, and the organic layer was collected and washed, firstly with dichloromethane, DCM (3 x 30 cm^3). The combined washes were then washed with deionized water (18.2 MΩ.cm) (15 x 30 cm^3). The dichloromethane, DCM solution was dried by addition of anhydrous sodium sulfate (*ca.* 10 g), then isolated *via* vacuum filtration of the Na_2SO_4 (P3 glass sintered funnel with Celite). The filtrate was transferred to a round bottom flask, and dichloromethane, DCM was removed *in vacuo* by rotary evaporation at (303.15 K) and further under high vacuum (48h, 343.15 K, 10^{-2} mbar), leaving clear liquid product. XRF analysis confirmed chloride content was below the detectable limit. ^1H, ^{13}C, ^{19}F and ^{31}P NMR spectra of the IL were recorded in acetonitrile-d_3.

^1H NMR: δ: 0.91 (m, 12H), 1.32 (m, 20H), 1.44 (m, 16H), 2.06 (m, 8H).

^{13}C NMR: (TFSI peaks 125.87, 122.67, 119.48, 116.29 (q, $1J_{C-F}$ = 321.79 Hz)), 14.36 (P-$(CH_2)_5$-***CH_3***), 14.50 (P-$(CH_2)_7$-***CH_3***), 19.27 (d, $1J_{C-P}$ = 48.27 Hz) (P-***CH_2***-$(CH_2)_4$-CH_3 and P-***CH_2***-$(CH_2)_6$-CH_3), 21.99 (d, $2J_{C-P}$ = 5.03 Hz) (P-CH_2-***CH_2***-$(CH_2)_3$-CH_3 and P-CH_2-***CH_2***-$(CH_2)_5$-CH_3), 23.10 (P-$(CH_2)_4$-***CH_2***-CH_3), 23.43 (P-$(CH_2)_6$-***CH_2***-CH_3), 29.43 (P-$(CH_2)_3$-***CH_2***-$(CH_2)_3$-CH_3), 29.71 (P-$(CH_2)_4$-***CH_2***-$(CH_2)_2$-CH_3), 31.00 (d, $3J_{C-P}$ = 15.08 Hz) (P-$(CH_2)_2$-***CH_2***-$(CH_2)_2$-CH_3), 31.29 (d, $3J_{C-P}$ = 15.08 Hz) (P-$(CH_2)_2$-***CH_2***-$(CH_2)_4$-CH_3), 31.70 (P-$(CH_2)_3$-***CH_2***-CH_2-CH_3), 32.57 (P-$(CH_2)_5$-***CH_2***-CH_2-CH_3).

^{19}F NMR: δ: -79.84

$^{31}P\{^1H\}$ NMR: δ: 33.46

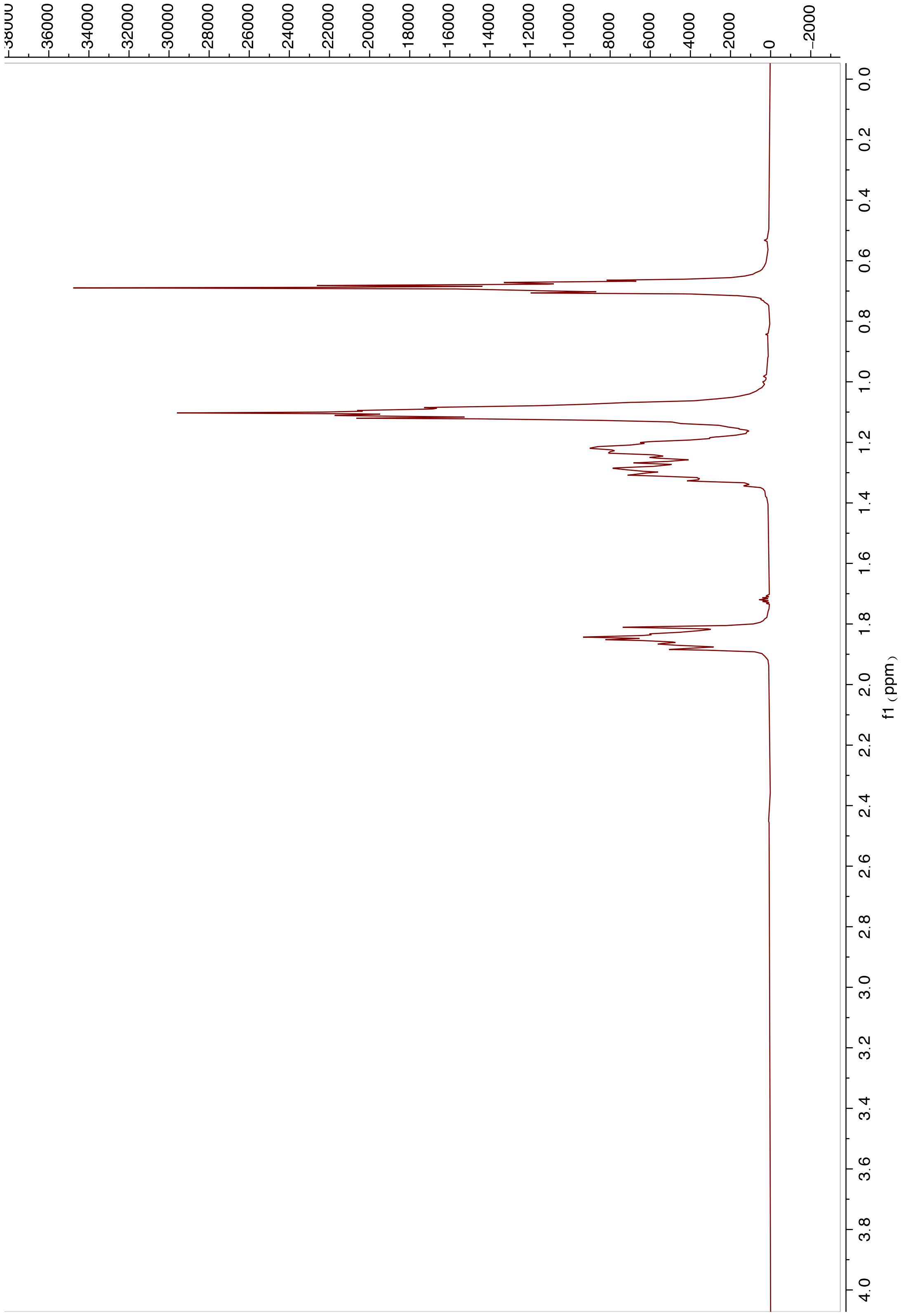

Figure S26. ^{1}H NMR spectrum (acetonitrile-d_3, 399.91 MHz, 300 K) of [$P_{666,8}$][TFSI].

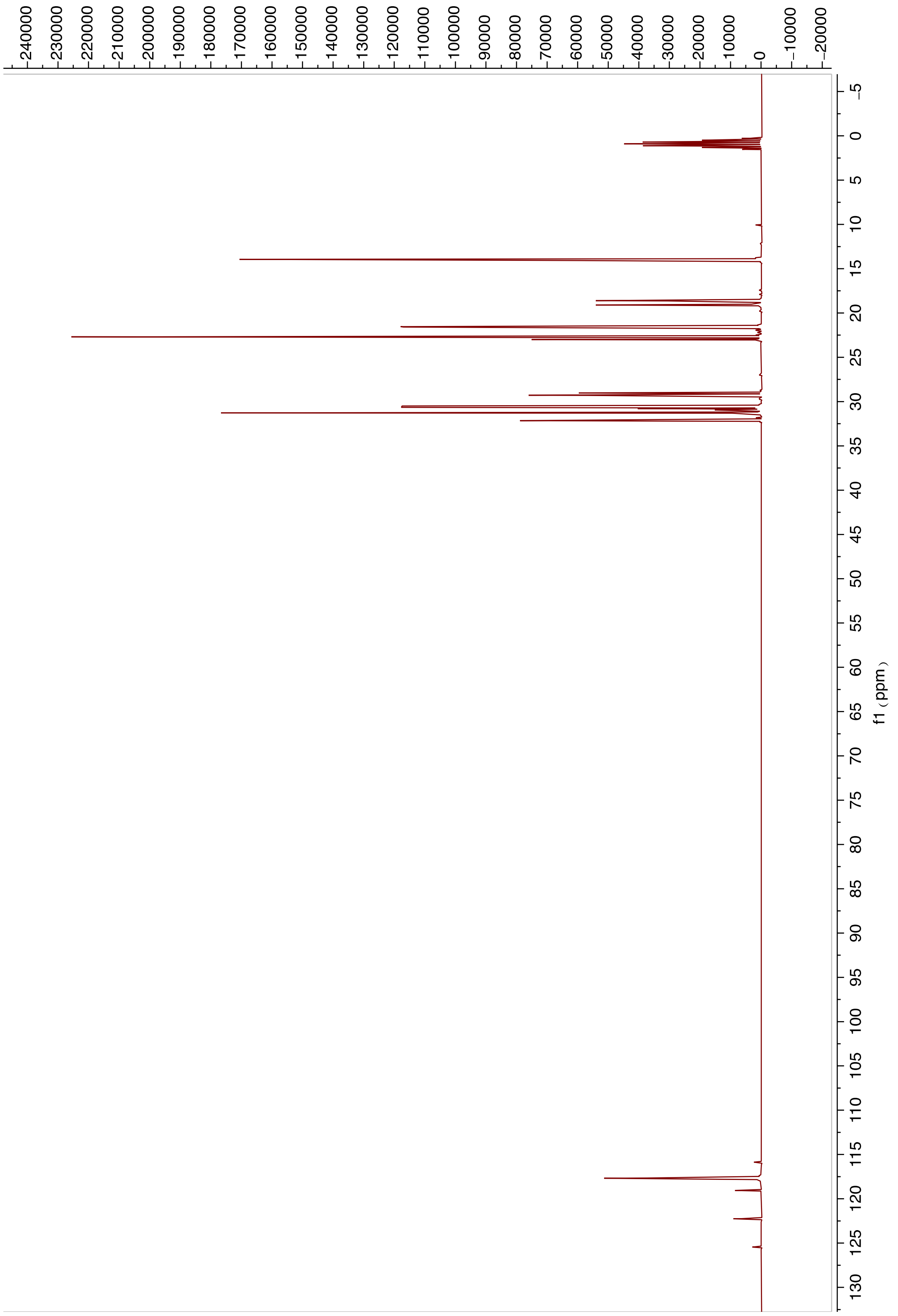

Figure S27. ^{13}C NMR spectrum (acetonitrile-d_3, 100.56 MHz, 301.1 K) of [$P_{666,8}$][TFSI].

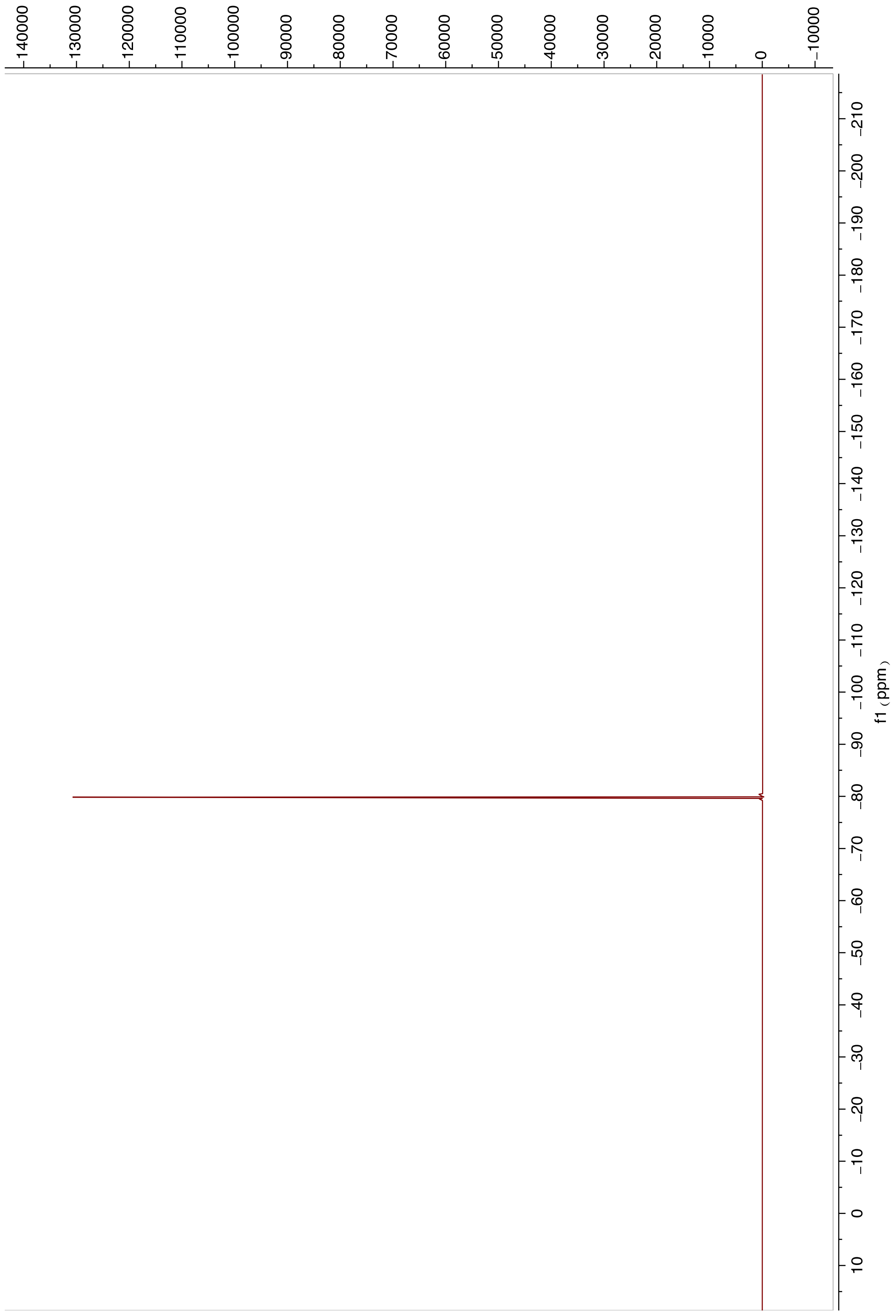

Figure S28. ^{19}F NMR spectrum (acetonitrile-d_3, 376.29 MHz, 300.2 K) of [$P_{666,8}$][TFSI].

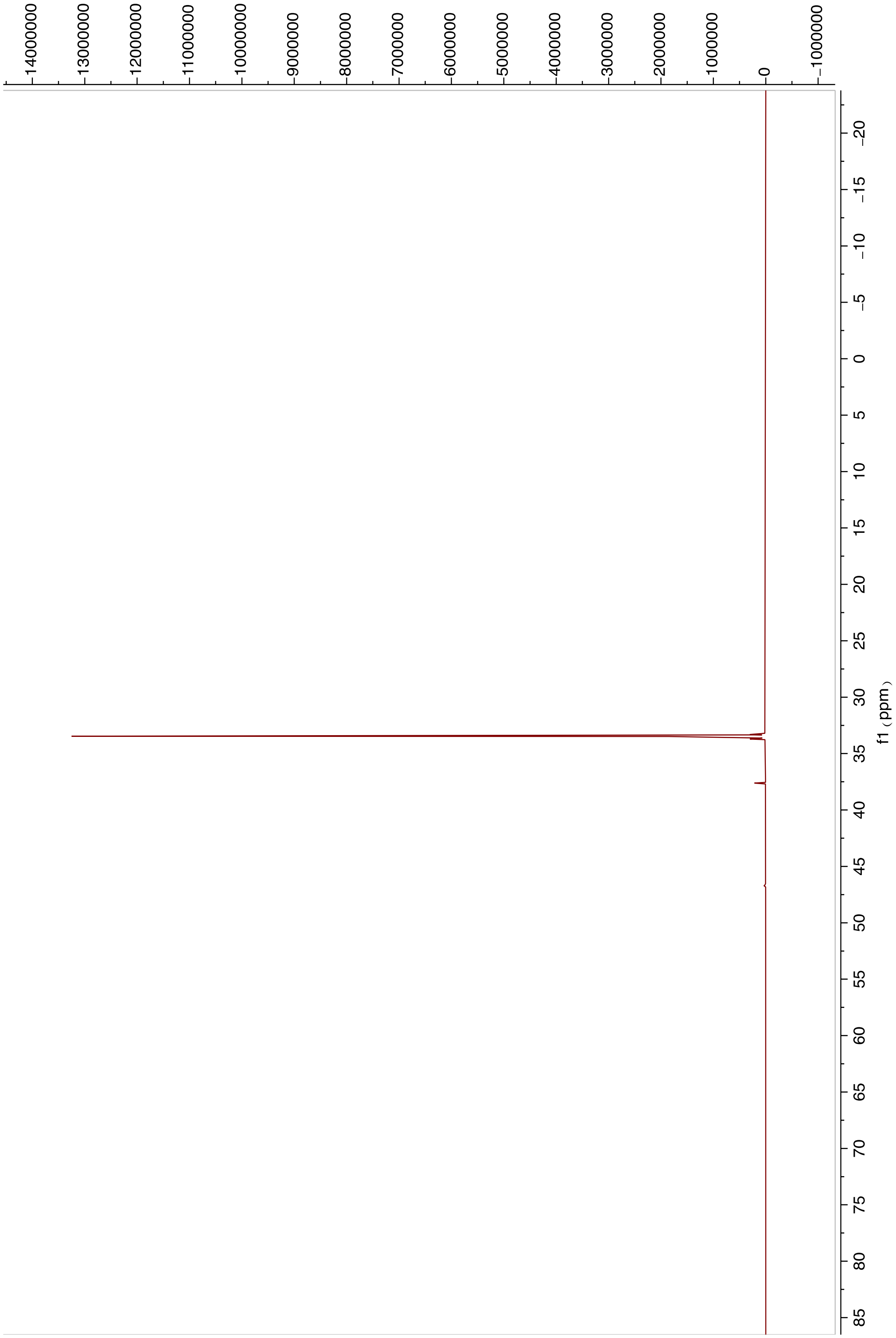

Figure S29 ^{31}P NMR spectrum (acetonitrile-d_3, 161.89 MHz, 300 K) of [$P_{666,8}$][TFSI].

[$P_{666,12}$]Cl. Trihexylphosphine (1.000 mol eq.) and 1-chlorododecane ($C_{12}H_{25}Cl$) (1.100 mol eq.) were placed in a round-bottomed flask (50 cm^3). The mixture was stirred and heated under a reflux condenser connected to a Schlenk line flushed with dinitrogen (16h, 418.15 K). In order to remove the excess of starting $C_{12}H_{25}Cl$, the purification step involved dissolution of the product in hexane, followed by extraction with water. The product was the dried at *in vacuo* (48 h, 338.15 K). ^{1}H, ^{13}C, and ^{31}P NMR spectra of the IL were recorded in acetonitrile-*d_3*.

^{1}H NMR: δ: 0.86 (m, 12H), 1.24 (m, 28H), 1.38 (m, 8H), 1.58 (m, 8H), 2.27 (m, 8H).

^{13}C NMR: δ: 14.25 (P-$(CH_2)_5$-***CH_3***), 14.36 (P-$(CH_2)_{11}$-***CH_3***), 19.34 (d, 1J_{C-P} = 47.26 Hz) (P-***CH_2***-$(CH_2)_4$-CH_3 and P-***CH_2***-$(CH_2)_{10}$-CH_3), 22.01 (d, 2J_{C-P} = 5.03 Hz) (P-CH_2-***CH_2***-$(CH_2)_3$-CH_3 and P-CH_2-***CH_2***-$(CH_2)_9$-CH_3), 22.95 (P-$(CH_2)_4$-***CH_2***-CH_3), 23.28 (P-$(CH_2)_{10}$-***CH_2***-CH_3), 29.40 (P-$(CH_2)_9$-***CH_2***-CH_2-CH_3), 29.90 (P-$(CH_2)_8$-***CH_2***-$(CH_2)_2$-CH_3), 29.97 (P-$(CH_2)_7$-***CH_2***-$(CH_2)_3$-CH_3), 30.18 (P-$(CH_2)_6$-***CH_2***-$(CH_2)_4$-CH_3), 30.24 (P-$(CH_2)_5$-***CH_2***-$(CH_2)_5$-CH_3), 30.27 (P-$(CH_2)_4$-***CH_2***-$(CH_2)_6$-CH_3), 30.98 (d, 3J_{C-P} = 15.08 Hz) (P-$(CH_2)_2$-***CH_2***-$(CH_2)_8$-CH_3), 31.26 (d, 3J_{C-P} = 15.08 Hz) (P-$(CH_2)_2$-***CH_2***-$(CH_2)_2$-CH_3), 31.61 (P-$(CH_2)_3$-***CH_2***-CH_2-CH_3), 32.53 (P-$(CH_2)_3$-***CH_2***-$(CH_2)_7$-CH_3).

$^{31}P\{^{1}H\}$ NMR: δ: 33.29

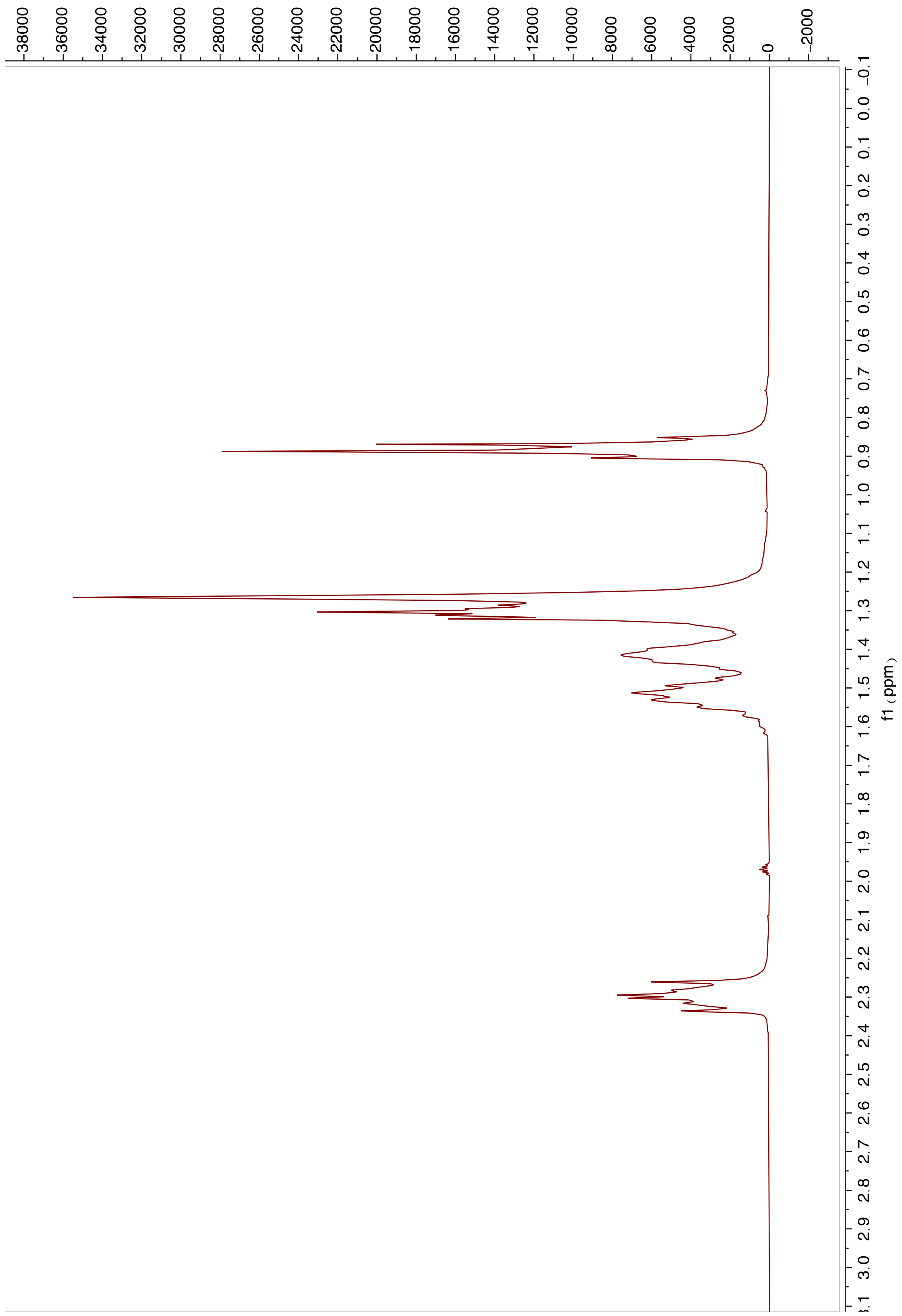

Figure S30. ^{1}H NMR spectrum (acetonitrile-d_3, 399.91 MHz, 299.9 K) of [$P_{666,12}$]Cl.

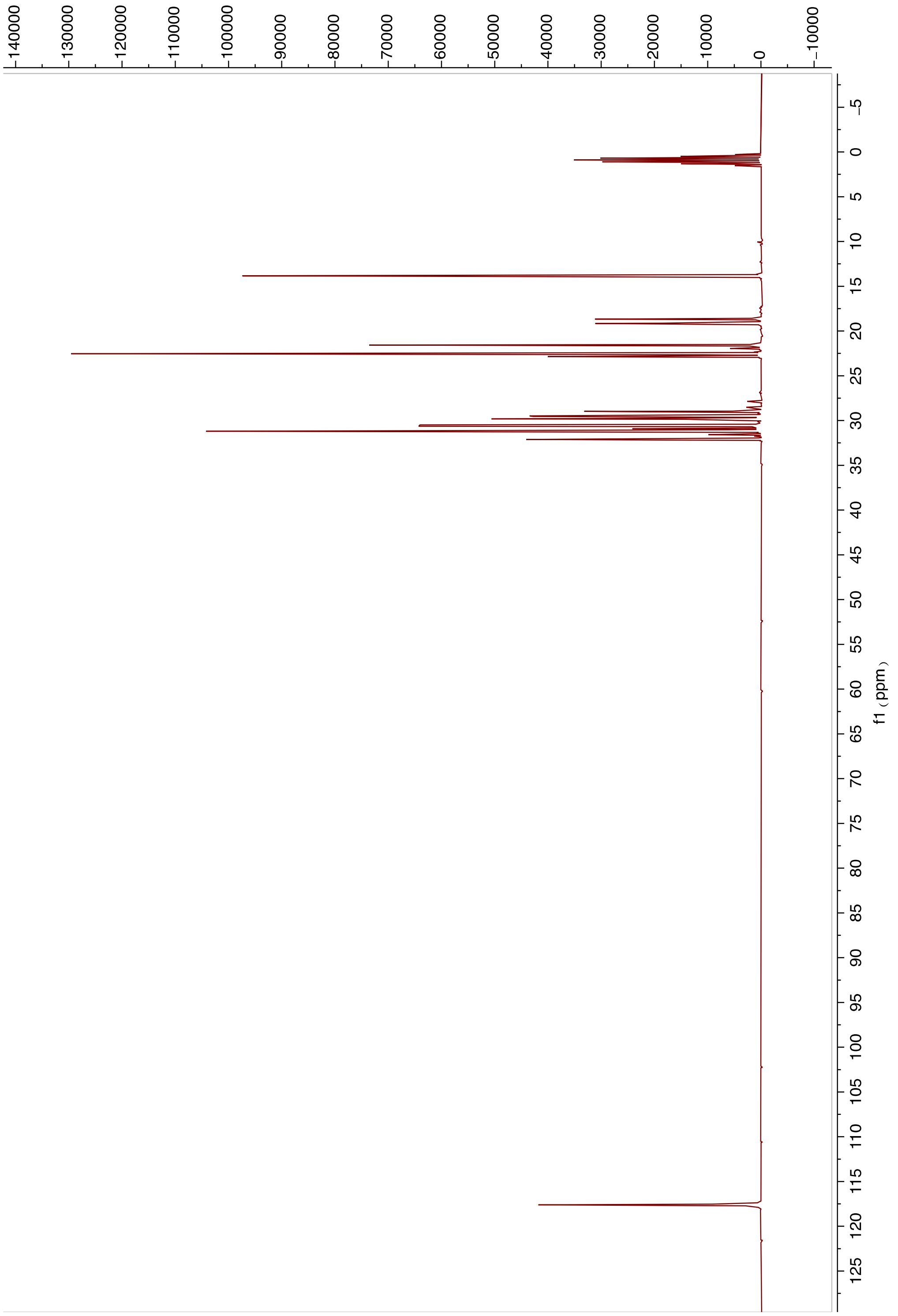

Figure S31. ^{13}C NMR spectrum (acetonitrile-d_3, 100.55 MHz, 301.2 K) of $[P_{666,12}]Cl$.

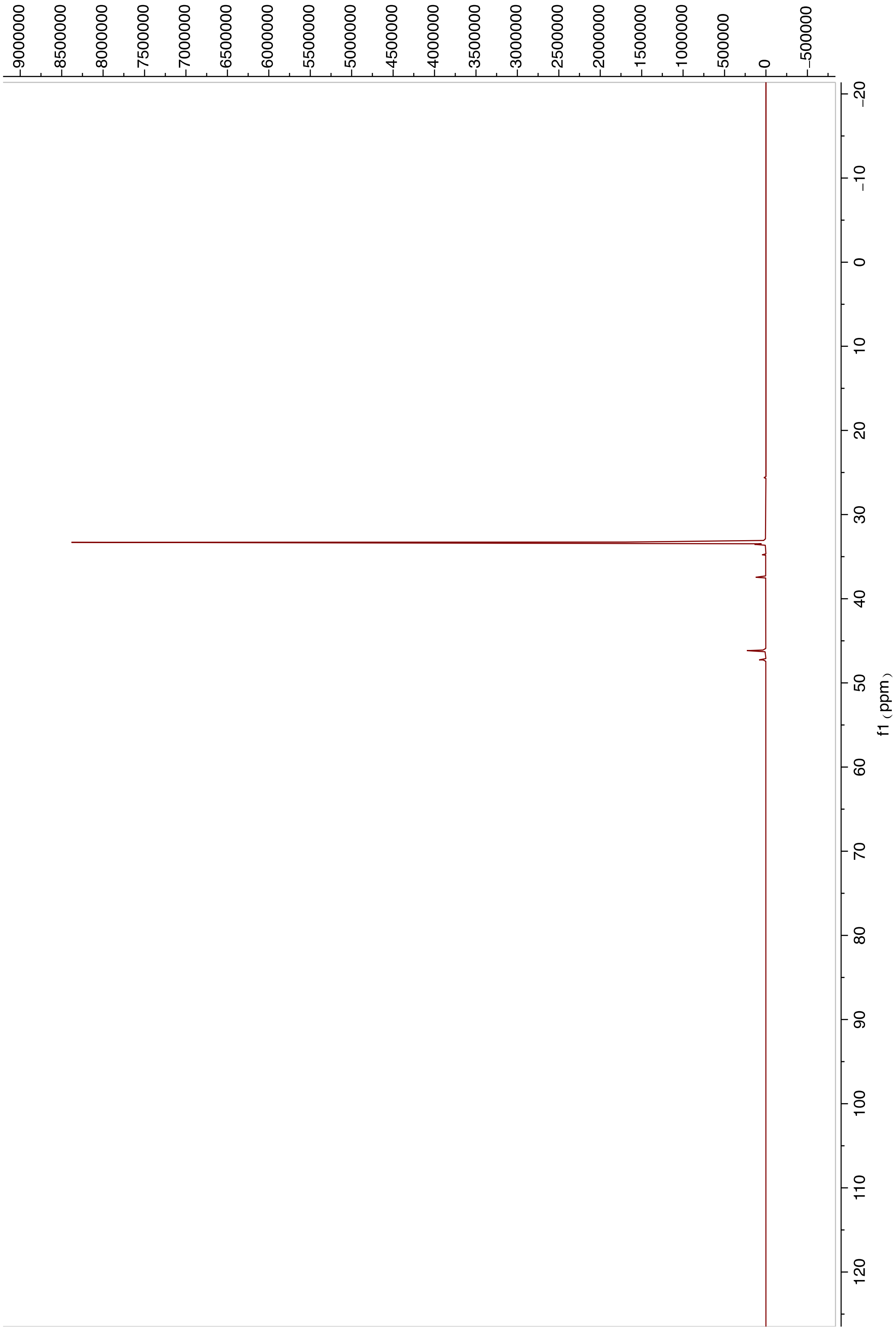

Figure S32. ^{31}P NMR spectrum (acetonitrile-d_3, 161.89 MHz, 300 K) of [$P_{666,12}$]Cl.

[$P_{666,12}$]TFSI]. Trihexyl(dodecyl)phosphonium chloride, [$P_{666,12}$]Cl (0.008 mol eq.) and lithium bis(trifluoromethanesulfon)imide Li[TFSI] (0.011 mol eq.) were separately dissolved in 25 cm^3 deionised water (18.2 MΩ.cm) (total 50 cm^3) and then combined in a round-bottomed flask (250 cm^3), resulting in the formation of a biphasic liquid system; the mixture was left to react (1 h, room temperature, 600 rpm). The aqueous layer was separated, and the organic layer was collected and washed, firstly with deionized water (18.2 MΩ.cm) (10 cm^3) and then dichloromethane, DCM (10 cm^3). Subsequent washes were performed with solution of Li[TFSI] in deionised water (18.2 MΩ.cm).12 Final three washes were performed with deionised water (18.2 MΩ.cm). The organic layer was washed 10 times in total. Subsequently, DCM was removed *via* rotary evaporation (30 min, 308.15 K) and the ionic liquid was dried under high vacuum (12h, 343.15 K, 10^{-2} mbar). XRF analysis confirmed chloride content was below the detectable limit. ^{1}H, ^{13}C, ^{19}F and ^{31}P NMR spectra of the IL were recorded in acetonitrile-d_3.

^{1}H NMR: δ: 0.91 (m, 12H), 1.29 (m, 28H), 1.43 (m, 16H), 2.05 (m, 8H).

^{13}C NMR: δ: (TFSI peaks 125.82, 122.63, 119.43, 116.24 (q, $1J_{C\text{-}F}$ = 320.79 Hz), 14.31 (P-$(CH_2)_5$-$\boldsymbol{CH_3}$), 14.48 (P-$(CH_2)_{11}$-$\boldsymbol{CH_3}$), 19.23 (d, $1J_{C\text{-}P}$ = 48.27 Hz) (P-$\boldsymbol{CH_2}$-$(CH_2)_4$-CH_3 and P-$\boldsymbol{CH_2}$-$(CH_2)_{10}$-CH_3), 21.94 (d, $2J_{C\text{-}P}$ = 4.02 Hz) (P-CH_2-$\boldsymbol{CH_2}$-$(CH_2)_3$-CH_3 and P-CH_2-$\boldsymbol{CH_2}$-$(CH_2)_9$-CH_3), 23.07 (P-$(CH_2)_4$-$\boldsymbol{CH_2}$-CH_3), 23.25 (P-$(CH_2)_{10}$-$\boldsymbol{CH_2}$-CH_3), 23.46 (P-$(CH_2)_9$-$\boldsymbol{CH_2}$-CH_2-CH_3), 29.43 (P-$(CH_2)_8$-$\boldsymbol{CH_2}$-$(CH_2)_2$-CH_3), 29.98 (P-$(CH_2)_7$-$\boldsymbol{CH_2}$-$(CH_2)_3$-CH_3), 30.15 (P-$(CH_2)_6$-$\boldsymbol{CH_2}$-$(CH_2)_4$-CH_3), 30.30 (P-$(CH_2)_5$-$\boldsymbol{CH_2}$-$(CH_2)_5$-CH_3), 30.41 (P-$(CH_2)_4$-$\boldsymbol{CH_2}$-$(CH_2)_6$-CH_3), 30.98 (d, $3J_{C\text{-}P}$ = 15.08 Hz) (P-$(CH_2)_2$-$\boldsymbol{CH_2}$-$(CH_2)_8$-CH_3), 31.23 (d, $3J_{C\text{-}P}$ = 15.08 Hz) (P-$(CH_2)_2$-$\boldsymbol{CH_2}$-$(CH_2)_2$-CH_3), 31.68 (P-$(CH_2)_3$-$\boldsymbol{CH_2}$-CH_2-CH_3), 32.72 (P-$(CH_2)_3$-$\boldsymbol{CH_2}$-$(CH_2)_7$-CH_3).

^{19}F NMR: δ: -79.98

^{31}P{^{1}H} NMR: δ: 33.52

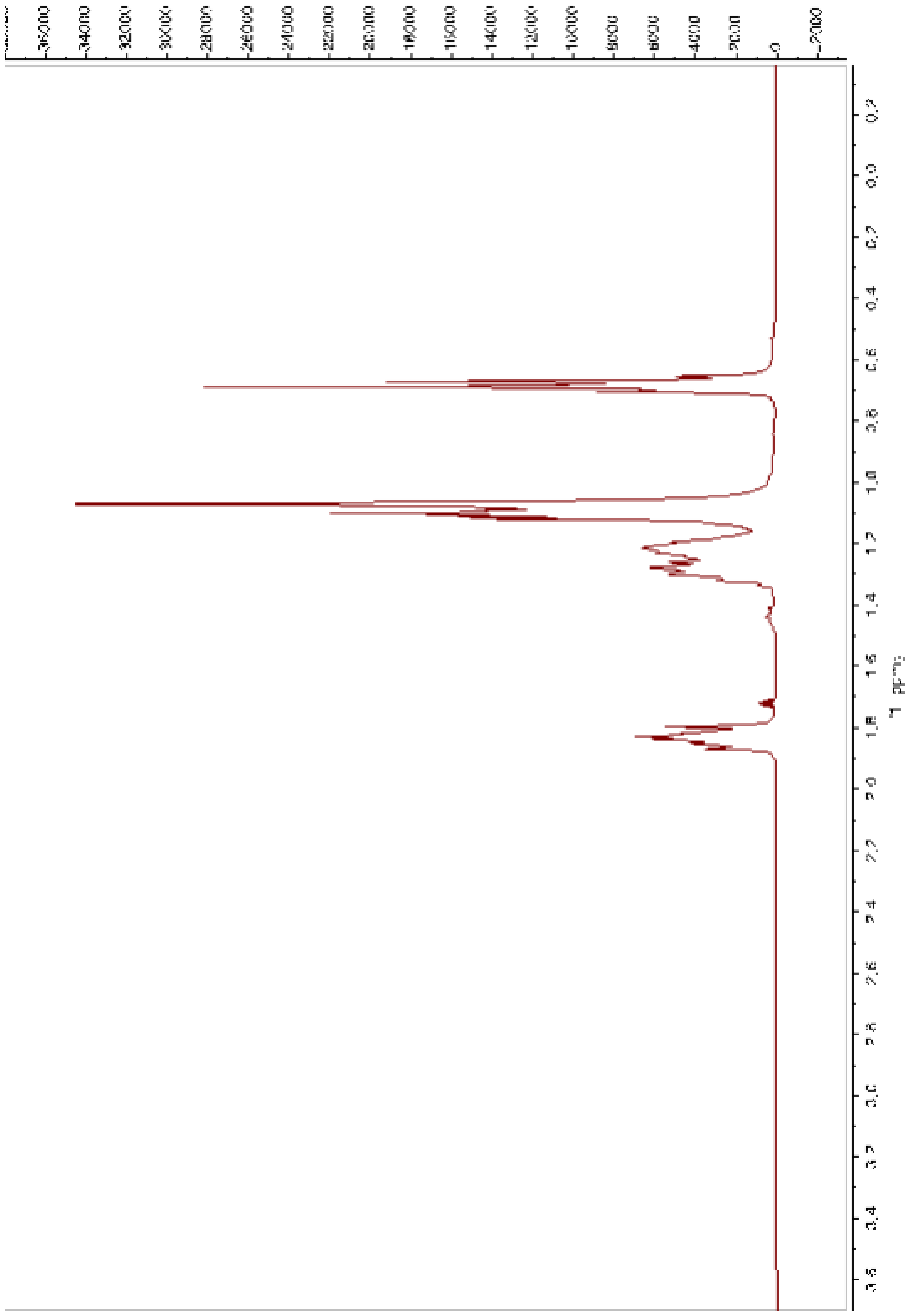

Figure S33. ^{1}H NMR spectrum (acetonitrile-d_3, 399.91 MHz, 300 K) of [$P_{666,12}$]TFSI].

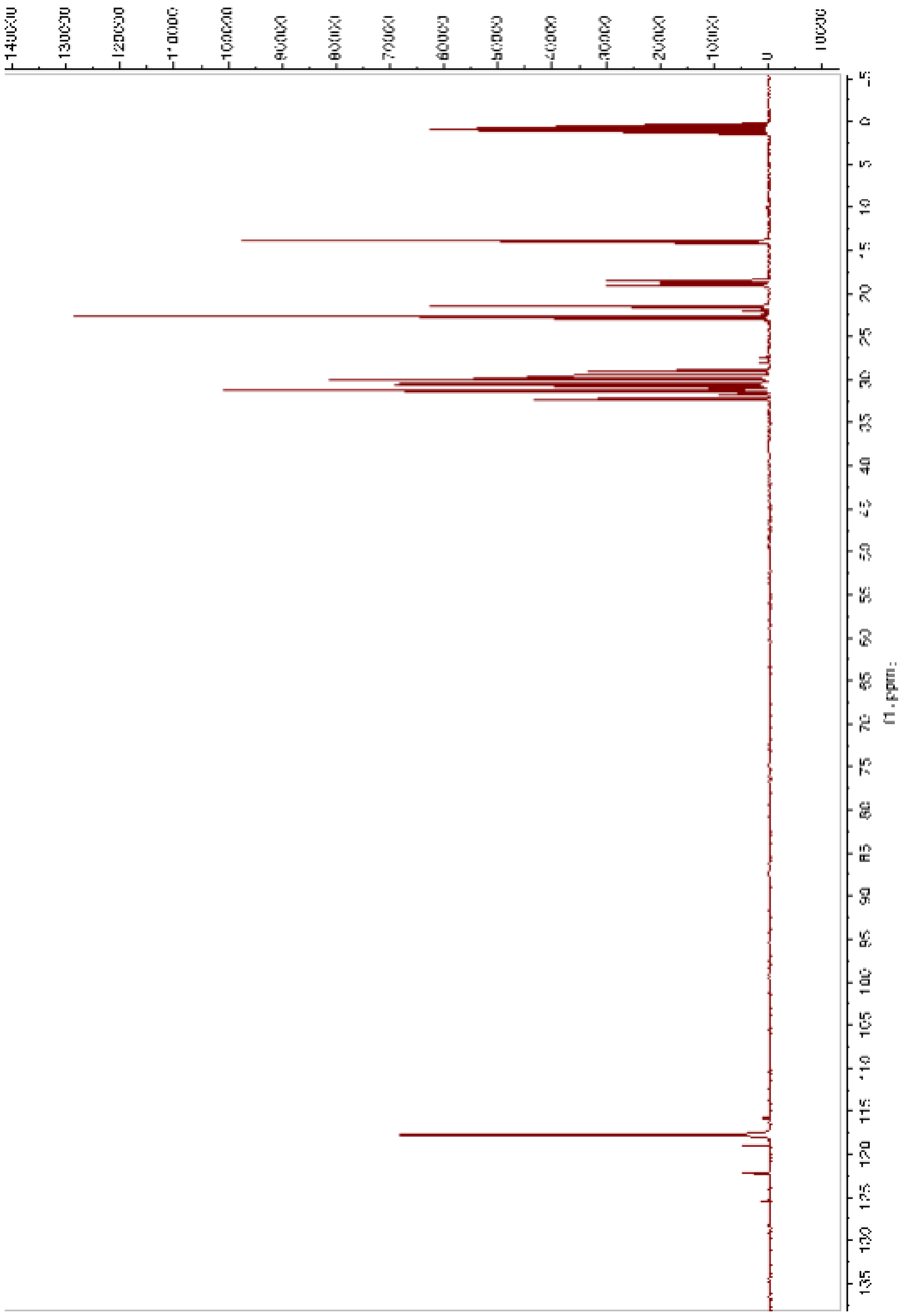

Figure S34. ^{13}C NMR spectrum (acetonitrile-d_3, 100.56 MHz, 300.4 K) of [$P_{666,12}$]TFSI].

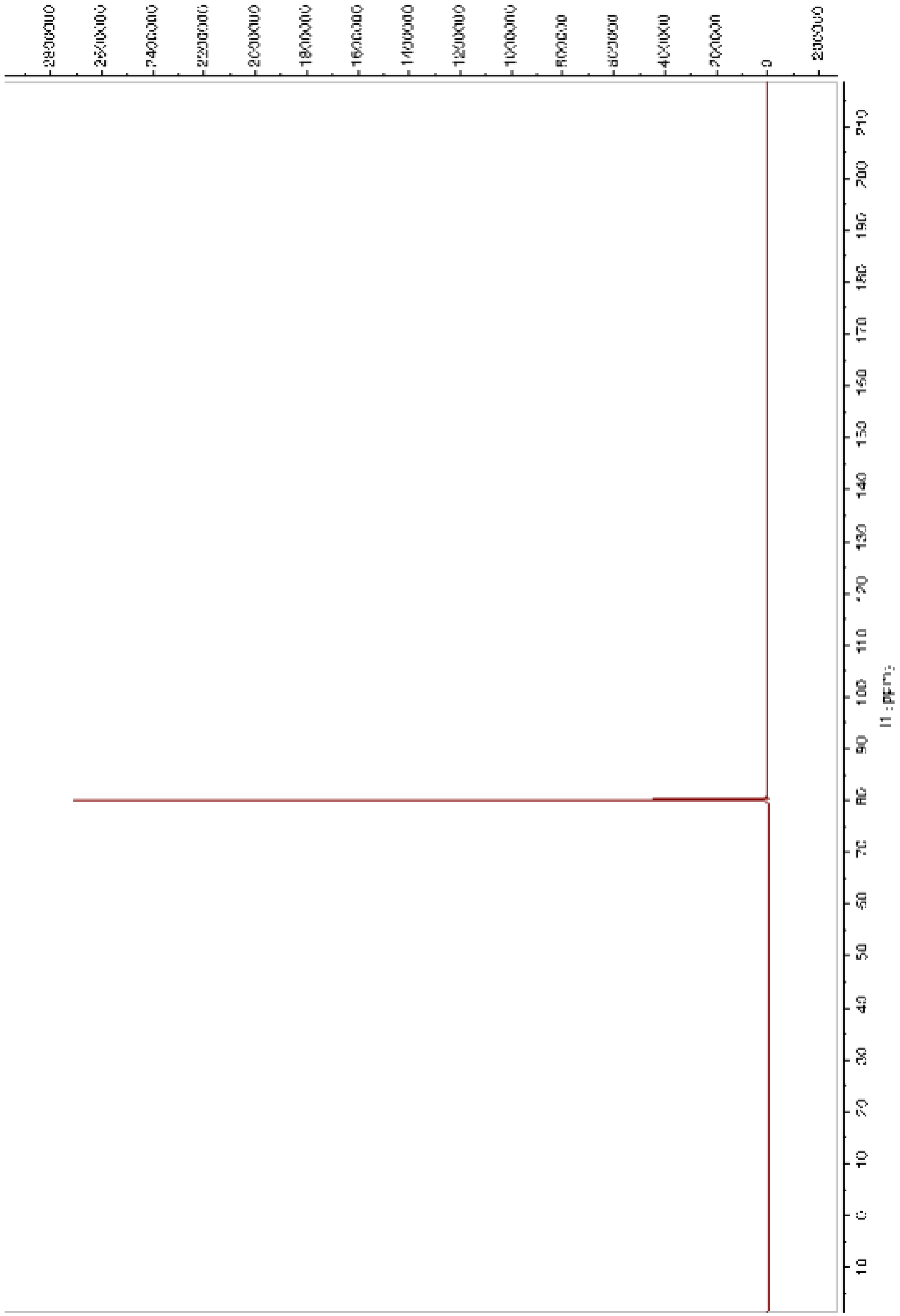

Figure S35. ^{19}F NMR spectrum (acetonitrile-d_3, 376.29 MHz, 299.7 K) of [$P_{666,12}$]TFSI].

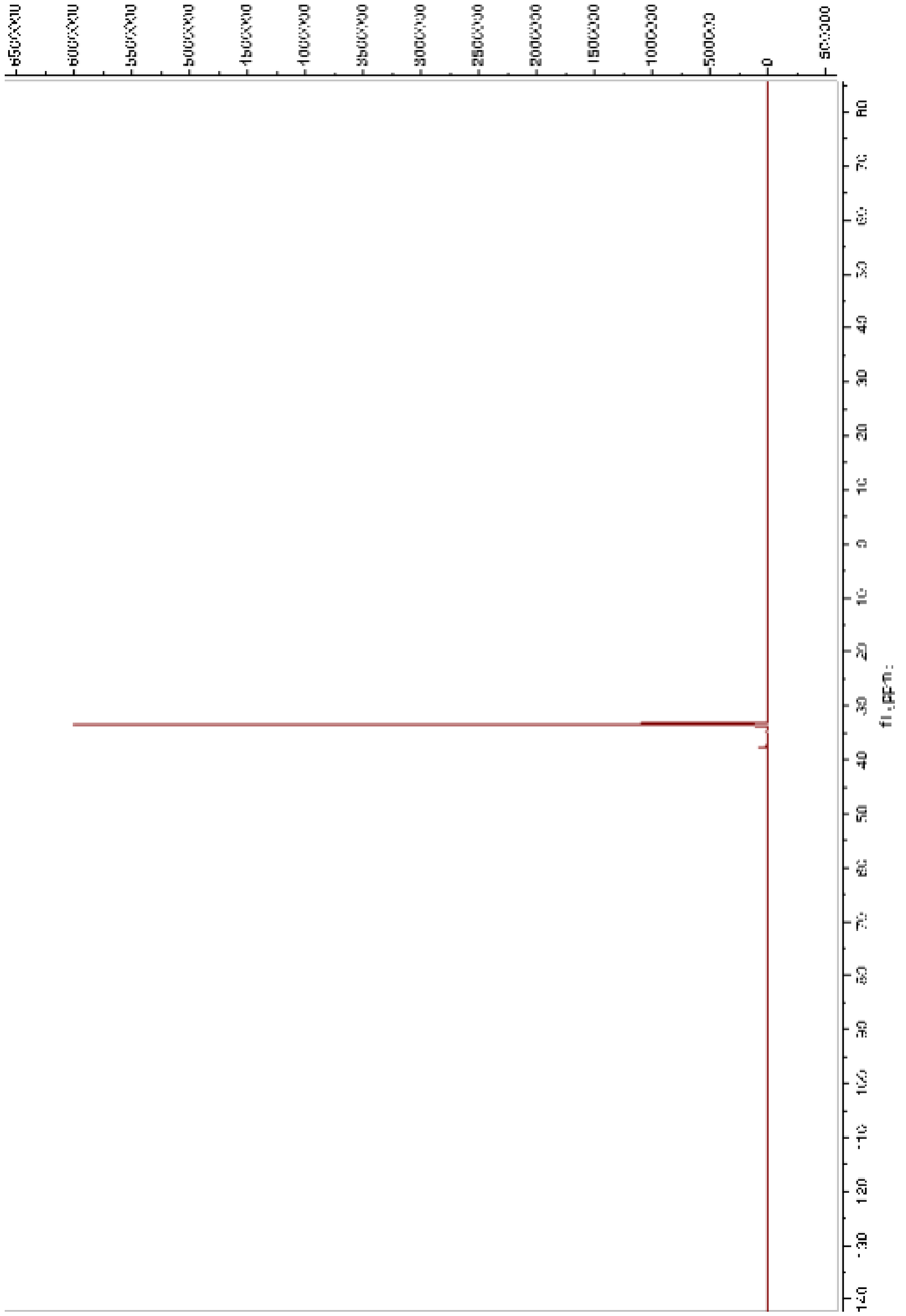

Figure S36. ^{31}P NMR spectrum (acetonitrile-d_3, 161.89 MHz, 302.4 K) of [$P_{666,12}$]TFSI].

Table S2. CHNS analysis of synthesized ionic liquids

	C / %		H / %		N / %		S / %	
	**calc.**	**exp.**	**calc.**	**exp.**	**calc.**	**exp.**	**calc.**	**exp.**
$[P_{444,14}][Cl]$	71.76	68.00	12.97	13.63	-	-	-	-
$[P_{444,14}][TFSI]$	49.47	51.36	8.30	9.25	2.06	1.82	9.43	9.94
$[P_{666,2}][TFSI]$	44.36	44.76	7.45	7.32	2.35	2.21	10.76	10.98
$[P_{666,6}][Cl]$	70.81	68.50	12.88	13.36	-	-	-	-
$[P_{666,6}][TFSI]$	47.91	48.66	8.04	8.89	2.19	1.89	9.84	10.09
$[P_{666,8}][TFSI]$	49.47	50.00	8.30	8.70	2.06	1.83	9.43	9.61
$[P_{666,12}][Cl]$	73.35	71.66	13.13	11.68	-	-	-	-
$[P_{666,12}][TFSI]$	52.22	52.91	8.76	9.80	1.90	1.53	8.71	8.64

Table S3. Identification of anions and cations by electrospray ionization mass spectrometry in both positive and negative modes.

	$\mathbf{ES^+_t}$	$\mathbf{ES^+_{exp}}$	$\mathbf{ES^-_t}$	$\mathbf{ES^-_{exp}}$
$[P_{444,14}][Cl]$	399.4115	399.3885	-	
$[P_{444,14}][TFSI]$	399.4115	400.3982	279.9178	279.9012
$[P_{666,2}][TFSI]$	315.3176	316.2943	279.9178	279.9164
$[P_{666,6}][Cl]$	371.3802	372.8089	-	-
$[P_{666,6}][TFSI]$	371.3802	371.3311	279.9178	279.9160
$[P_{666,8}][TFSI]$	399.4115	400.3738	279.9178	281.9225
$[P_{666,12}][Cl]$	455.4741	456.4358	-	-
$[P_{666,12}][TFSI]$	455.4741	456.4536	279.9178	281.9193
